# Supplementary material for: Do community-based active case-finding interventions have indirect impacts on wider TB case detection and determinants of subsequent TB testing behaviour? A systematic review
Source: PLOS Glob Public Health. 2021 Dec 8;1(12):e0000088. doi: 10.1371/journal.pgph.0000088 (PMC10021508; doi:10.1371/journal.pgph.0000088)
Supplement: S1 Checklist — (PDF) [file pgph.0000088.s001.pdf]

PRISMA 2009 Checklist

Section/topic	#	Checklist item	Reported on page #
**TITLE**			
Title	1	**Identify the report as a systematic review, meta-analysis, or both.**<br><br>Do community-based tuberculosis active case-finding interventions affect subsequent health-seeking behaviour? A systematic review	Yes (page 1)
**ABSTRACT**			
Structured summary	2	**Provide a structured summary including, as applicable: background; objectives; data sources; study eligibility criteria, participants, and interventions; study appraisal and synthesis methods; results; limitations; conclusions and implications of key findings; systematic review registration number.**<br><br>Done – see paper abstract	p. 3-4
**INTRODUCTION**			
Rationale	3	**Describe the rationale for the review in the context of what is already known.**<br><br>The effect of ACF on subsequent health-seeking behaviour has not previously been reviewed. We therefore aimed to systematically review the evidence of indirect effect of ACF on routine facility-based TB case notifications and proxy behavioural outcomes such as knowledge, attitudes and perceptions (KAP) that could inform the mechanisms of any effect on subsequent health-seeking behaviour.	6
Objectives	4	**Provide an explicit statement of questions being addressed with reference to participants, interventions, comparisons, outcomes, and study design (PICOS).**<br><br>Methods includes all this information (too long to usefully copy and paste excerpts).	
**METHODS**			
Protocol and registration	5	Indicate if a review protocol exists, if and where it can be accessed (e.g., Web address), and, if available, provide registration information including registration number.<br><br>No formal protocol exists, although concept notes were shared with WHO in the lead up to the commissioning of review.	

PRISMA 2009 Checklist

Eligibility criteria	6	**Specify study characteristics (e.g., PICOS, length of follow-up) and report characteristics (e.g., years considered, language, publication status) used as criteria for eligibility, giving rationale.**<br><br>See paragraph within methods entitled "Inclusion and exclusion criteria"	10
Information sources	7	**Describe all information sources (e.g., databases with dates of coverage, contact with study authors to identify additional studies) in the search and date last searched.**<br><br>"The literature search included all studies identified in a previous review by Kranzer et al in 2013 (6), covering the period 1 Jan 1980 to Oct 13 2010, and an additional search of PubMed, EMBASE, Scopus and the Cochrane Library for papers published between 1 Nov 2010 and 4 Feb 2020 (subsequently updated to 13 April 2020) (search strategy in Appendix 1)."<br><br>"Reference lists from eligible manuscripts were examined and expert opinion on other available papers was sought from members of the WHO TB Screening Guideline Development Group for this and the accompanying review on TB ACF effectiveness."	10-11
Search	8	**Present full electronic search strategy for at least one database, including any limits used, such that it could be repeated.**<br><br>In appendices 1 & 2	Appendices 1 & 2
Study selection	9	**State the process for selecting studies (i.e., screening, eligibility, included in systematic review, and, if applicable, included in the meta-analysis).**<br>"Studies identified through the updated search were title and abstract double screened for eligibility by FN, AES and LHC and then the full text of these and all studies from the Kranzer and colleagues review were reviewed by two of HRAF, RMB and MN. Inclusion decisions were resolved by consensus and discussion with ELC and PM."	10
Data collection process	10	**Describe method of data extraction from reports (e.g., piloted forms, independently, in duplicate) and any processes for obtaining and confirming data from investigators.**<br><br>"Data was extracted from studies independently by two of HRAF, RMB and MN and entered into a spreadsheet."	10
Data items	11	**List and define all variables for which data were sought (e.g., PICOS, funding sources) and any assumptions and simplifications made.**	8-9 & 11

		The outcomes were "routinely-diagnosed TB case notifications and proxy behavioural outcomes."<br><br>"To establish routinely diagnosed case notification rates, person-years of follow-up and notified TB cases diagnosed only through routine screening activities were extracted or calculated from available data using simple arithmetic. None of the studies presented case notification ratios for routine diagnosis; we calculated these from the available overall and ACF-specific case notification data."<br><br>"The proxy behavioural outcomes we examined were knowledge, attitudes and practices (KAP) within communities, ever-tested for TB, recent testing for TB, TB stigma and social norms."<br><br>"We classified studies according to level of healthcare access within the target population based on distance to and cost of care on a scale of 'Adequate' (routine free healthcare available within catchment area), 'Restricted' (access restricted due to distance and/or cost) or 'Hard to reach' (populations specifically selected as hard to reach)."	
Risk of bias in individual studies	12	**Describe methods used for assessing risk of bias of individual studies (including specification of whether this was done at the study or outcome level), and how this information is to be used in any data synthesis.**<br>"For randomised studies, the Cochrane Risk of Bias (ROB) tool was used to assess risk of bias. Non-randomised studies were assessed for risk of bias using ROBINS-I and qualitative studies were assessed through the Critical Appraisal Skills Programme (CASP) checklist."	12
Summary measures	13	**State the principal summary measures (e.g., risk ratio, difference in means).**<br><br>"For randomised and before-after studies we calculated the CNR ratio (intervention vs control groups or baseline vs post intervention populations) and for controlled before-after studies with a non-randomised comparison group the outcome measure was a comparison of the before to after TB CNR ratio in the two comparison groups: the ratio of the CNR ratios."	11-12
Synthesis of results	14	**Describe the methods of handling data and combining results of studies, if done, including measures of consistency (e.g., I^2) for each meta-analysis.**<br><br>"Where data was available confidence intervals were calculated using Stata. For studies affected by clustering, three possible values (0.01, 0.05 and 0.1) of the intra-cluster correlation coefficient (ICC) were estimated and used to calculated three possible confidence intervals using the Cochrane recommended method… Confidence intervals for KAP scores are presented as reported by the authors."	12

PRISMA 2009 Checklist

Section/topic	#	Checklist item	Reported on page #
Risk of bias across studies	15	**Specify any assessment of risk of bias that may affect the cumulative evidence (e.g., publication bias, selective reporting within studies).**<br><br>NA	N/A
Additional analyses	16	**Describe methods of additional analyses (e.g., sensitivity or subgroup analyses, meta-regression), if done, indicating which were pre-specified.**<br><br>We classified studies according to level of healthcare access within the target population based on distance to and cost of care on a scale of 'Adequate' (routine free healthcare available within catchment area), 'Restricted' (access restricted due to distance and/or cost) or 'Hard to reach' (populations specifically selected as hard to reach). Outcome measures did not appear to be associated with reported healthcare accessibility.	11
**RESULTS**			
Study selection	17	**Give numbers of studies screened, assessed for eligibility, and included in the review, with reasons for exclusions at each stage, ideally with a flow diagram.**<br><br>PRISMA diagram is figure 2	Fig 2
Study characteristics	18	**For each study, present characteristics for which data were extracted (e.g., study size, PICOS, follow-up period) and provide the citations.**<br>Table 1	Table 1
Risk of bias within studies	19	**Present data on risk of bias of each study and, if available, any outcome level assessment (see item 12).**<br>Figure 6	Fig 6
Results of individual studies	20	**For all outcomes considered (benefits or harms), present, for each study: (a) simple summary data for each intervention group (b) effect estimates and confidence intervals, ideally with a forest plot.**<br><br>Table 2 & Figures 4 & 5	Table 2<br>Figs 4 & 5
Synthesis of results	21	**Present results of each meta-analysis done, including confidence intervals and measures of consistency.**<br><br>No meta-analysis done; "The small number of published studies that specifically address this important issue were at risk of bias introduced by the design or completeness of evaluation, and critical differences in study design precluded meta-analysis	NA – no meta-analysis

Risk of bias across studies	22	**Present results of any assessment of risk of bias across studies (see Item 15).**<br><br>NA	NA
Additional analysis	23	**Give results of additional analyses, if done (e.g., sensitivity or subgroup analyses, meta-regression [see Item 16]).**<br><br>Table.1 specifies level of healthcare access for the population each study was conducted on.	Table 1
**DISCUSSION**			
Summary of evidence	24	**Summarize the main findings including the strength of evidence for each main outcome; consider their relevance to key groups (e.g., healthcare providers, users, and policy makers).**<br>Our main finding was the need for more evidence: we found mixed weak evidence that TB ACF may be effective at indirectly increasing routine TB case notification rates for non-bacteriologically confirmed TB, and insufficient evidence to conclude whether or not ACF impacts subsequent health-seeking behaviour.	25
Limitations	25	**Discuss limitations at study and outcome level (e.g., risk of bias), and at review-level (e.g., incomplete retrieval of identified research, reporting bias).**<br><br>Limited number of studies reporting on relevant outcomes with wide range of study designs and interventions meant meta-analysis was not appropriate. A high proportion of studies were at serious or critical risk of bias and there was limited availability to adjust for confounders as this data was not consistently reported.	28
Conclusions	26	**Provide a general interpretation of the results in the context of other evidence, and implications for future research.**<br><br>ACF has potential to impact subsequent health-seeking behaviour through an increase in TB knowledge, earlier care-seeking if TB symptoms are detected, or follow-up after a negative ACF test, but reporting of the impact on routinely diagnosed TB case-notifications is limited and only one trial addressed proxy behavioural outcomes. Evaluation of routine TB testing and other proxy behavioural outcomes in ACF and comparator communities should be included as standard in study designs.	29
**FUNDING**			
Funding	27	**Describe sources of funding for the systematic review and other support (e.g., supply of data); role of funders for the systematic review.**<br><br>This research was funded in part by WHO to inform their TB screening guideline development process. WHO facilitated discussions among authors at design stage, but had no role in conduct of review.	12

PRISMA 2009 Checklist

From: Moher D, Liberati A, Tetzlaff J, Altman DG, The PRISMA Group (2009). Preferred Reporting Items for Systematic Reviews and Meta-Analyses: The PRISMA Statement. PLoS Med 6(7): e1000097. doi:10.1371/journal.pmed1000097

For more information, visit: **www.prisma-statement.org**.
