# Supplementary material for: Do community-based active case-finding interventions have indirect impacts on wider TB case detection and determinants of subsequent TB testing behaviour? A systematic review
Source: PLOS Glob Public Health. 2021 Dec 8;1(12):e0000088. doi: 10.1371/journal.pgph.0000088 (PMC10021508; doi:10.1371/journal.pgph.0000088)
Supplement: S1 Text — (PDF) [file pgph.0000088.s002.pdf]

Appendix 1: Main Search Strategy

Search strategy
Databases
PubMed, EMBASE, Scopus, Cochrane Library

PubMed

#1	"tuberculosis"[MeSH Terms]
#2	"tuberculosis"[tw] OR "Pulmonary Consumption"[tw] OR "Consumption, Pulmonary"[tw] OR Phthisis[tw] OR "Tuberculoses"[tw] OR "MDR-TB"[tw] OR "XDR-TB"[tw] OR "MDR TB"[tw] OR "XDR TB"[tw]
#3	#1 OR #2
#4	"Mass Screening"[MeSH Terms] OR "Mass Chest X-Ray"[MeSH Terms] OR "contact tracing"[MeSH Terms] OR "health surveys"[MeSH Terms] OR "Cross-Sectional Studies"[MeSH Terms] OR "Epidemiologic Studies"[MeSH Terms]
#5	"Mass Chest X Ray"[tw] OR "Mass Chest X-Rays"[tw] OR "screenings"[tw] OR "screening"[tw] OR "cross-sectional"[tw] OR "case-detection"[tw] OR "case finding"[tw] OR "contact tracing"[tw] OR "health survey"[tw] OR "prevalence survey"[tw] OR "prevalence studies"[tw] OR "mass radiography"[tw] OR "contact examination"[tw]
#6	#4 OR #5
#7	#3 AND #6
#8	("animals"[MeSH Terms] NOT ("humans"[MeSH Terms] AND "animals"[MeSH Terms]))
#9	#7 NOT #8
#10	("2010/11/01"[EDAT] : "3000/12/31"[EDAT] OR "2010/11/01"[CRDT] : "3000/12/31"[CRDT]) OR ("2010/11/01"[PDAT] : "3000/11/31"[PDAT])
#11	#9 AND #10

Embase

#1	'tuberculosis'/exp OR 'lung tuberculosis'/exp
#2	('tuberculosis' OR 'Pulmonary Consumption' OR 'Consumption, Pulmonary' OR Phthisis OR 'Tuberculoses' OR "MDR-TB" OR "XDR-TB" OR "MDR TB" OR "XDR TB"):ab,ti,kw
#3	#1 OR #2
#4	'tuberculosis control'/exp OR 'case finding'/exp OR 'mass radiography'/exp OR 'mass screening'/exp OR 'contact examination'/exp OR 'screening'/exp
#5	('Mass Chest X Ray' OR 'Mass Chest X-Rays' OR 'Screenings' OR 'screening' OR 'Cross-Sectional Studies' OR 'Case-detection' OR 'case finding' OR 'contact tracing' OR 'mass radiography' OR 'contact examination' OR 'health survey' OR 'cross-sectional' OR 'prevalence survey' OR 'prevalence studies'):ab,ti,kw
#6	#4 OR #5
#7	#3 AND #6
#8	'animal'/exp NOT ('animal'/exp AND 'human'/exp)
#9	#7 NOT #8
#10	[1-11-2010]/sd
#11	#9 AND #10

Scopus

#1	TITLE-ABS-KEY (tuberculosis OR phthisis OR "pulmonary consumption" OR Tuberculoses OR “MDR-TB” OR “XDR-TB” OR “MDR TB” OR “XDR TB”)
#2	TITLE-ABS-KEY("mass chest x ray" OR "mass chest x-rays" OR screenings OR screening OR "health survey" OR "cross-sectional" OR "case-detection" OR "case finding" OR "contact tracing" OR "prevalence survey" OR "prevalence studies" OR "mass radiography" OR "contact examination")
#3	#1 AND #2
#4	PUBDATETXT (november 2010) OR PUBDATETXT (december 2010) OR PUBYEAR > 2010
#5	#3 AND #4

removed b/c redundant:
(mass screenings) OR (mass screening)
(cross-sectional studies)
(active case finding)
(intensified case-finding) OR (intensified case finding)
(contact screening)
(population screening)

Cochrane Library

#1 MeSH descriptor: [Tuberculosis] explode all trees
#2 "tuberculosis" OR (Pulmonary NEXT Consumption*) OR Phthisis OR Tuberculoses OR “MDR-TB” OR “XDR-TB” OR “MDR TB” OR “XDR TB”
#3 #1 OR #2
#4 MeSH descriptor: [Mass Screening] explode all trees
#5 MeSH descriptor: [Mass Chest X-Ray] explode all trees
#6 MeSH descriptor: [Contact Tracing] explode all trees
#7 MeSH descriptor: [Health Surveys] explode all trees
#8 MeSH descriptor: [Cross-Sectional Studies] explode all trees
#9 MeSH descriptor: [Epidemiologic Studies] explode all trees
#10 “Mass Chest X Ray” OR “Mass Chest X-Rays” OR “screenings” OR “screening” OR “cross-sectional” OR “case-detection” OR “case finding” OR “contact tracing” OR “health survey” OR "prevalence survey" OR “prevalence studies” OR "mass radiography" OR "contact examination"
#11 {OR #4-#10}
#12 #3 AND #11 with Cochrane Library publication date Between Nov 2010 and Mar 2019
