# Supplementary material for: Do community-based active case-finding interventions have indirect impacts on wider TB case detection and determinants of subsequent TB testing behaviour? A systematic review
Source: PLOS Glob Public Health. 2021 Dec 8;1(12):e0000088. doi: 10.1371/journal.pgph.0000088 (PMC10021508; doi:10.1371/journal.pgph.0000088)
Supplement: S2 Text — (PDF) [file pgph.0000088.s003.pdf]

Appendix 2: Accompanying qualitative and KAP studies search strategy

<u>Pubmed</u>

For each study in Appendix 4

#1	"any reported study name"<br>(e.g. "DETECTB")
#2	Paper first author [Author]<br>(e.g. Corbett EL[Author])
#3	Paper last author [Author]<br>(e.g. Hayes RJ [Author])
#4	#2 OR #3
#5	"Tuberculosis"
#6	"study location"<br>(e.g. "Zimbabwe")
#7	"year study completed/01/01"[Date - MeSH] : "3000"[Date - MeSH]<br>(e.g. "2009/01/01"[Date - MeSH] : "3000"[Date - MeSH])
#8	#4 AND #5 AND #6 AND #7
#9	#1 OR #8
