# Supplementary material for: Do community-based active case-finding interventions have indirect impacts on wider TB case detection and determinants of subsequent TB testing behaviour? A systematic review
Source: PLOS Glob Public Health. 2021 Dec 8;1(12):e0000088. doi: 10.1371/journal.pgph.0000088 (PMC10021508; doi:10.1371/journal.pgph.0000088)
Supplement: S1 Table — (PDF) [file pgph.0000088.s004.pdf]

Appendix 3

List of papers about TB ACF reviewed at full text.

Author	Year	Journal	Title	Decision	mainreason	Which review?
Abascal et. al	2020	Sci Rep	Screening of inmates transferred to Spain reveals a Peruvian prison as a reservoir of persistent Mycobacterium tuberculosis MDR strains and mixed infections	exclude	No comparison group	NA
Abbara et. al	2020	Int J Infect Dis	The challenges of tuberculosis control in protracted conflict: The case of Syria	exclude	No relevant data / not an ACF intervention	NA
Abdulkareem et. al	2020	Int J Infect Dis	First insight into latent tuberculosis infection among household contacts of tuberculosis patients in Duhok, Iraqi Kurdistan: using tuberculin skin test and QuantiFERON-TB Gold Plus test	exclude	No comparison group	NA
Abdurrahman et. al	2017	New microbes and new infections	Are patients with pulmonary tuberculosis who are identified through active case finding in the community different than those identified in healthcare facilities?	exclude	No comparison group	NA
Abebe et. al	2011	The international journal of tuberculosis and lung disease : the official journal of the International Union against Tuberculosis and Lung Disease	Prevalence of pulmonary tuberculosis and associated risk factors in Eastern Ethiopian prisons	exclude	No comparison group	NA
Abebe et. al	2012	BMC public health	Tuberculosis lymphadenitis in Southwest Ethiopia: a community based cross-sectional study	exclude	No comparison group	NA
Abera et. al	2018	The open microbiology journal	Pulmonary Tuberculosis and Associated Factors Among Diabetic Patients Attending Hawassa Adare Hospital, Southern Ethiopia	exclude	No comparison group	NA
Abseno et. al	2014	Ethiopian medical journal	Tuberculosis among Addis Ababa city bus drivers and cash collectors	exclude	No comparison group	NA
Abubakar et. al	2011	Eurosurveillance	Assessing the efect of foreign travel and protection by BCG vaccination on the spread of tuberculosis in a low incidence country, United Kingdom, October 2008 to December 2009	exclude	No comparison group	NA
Abuogi et. al	2013	The international journal of tuberculosis and lung disease : the official journal of the International Union against Tuberculosis and Lung Disease	Impact of expanded antiretroviral use on incidence and prevalence of tuberculosis in children with HIV in Kenya	exclude	Healthcare based screening	NA

Author	Year	Journal	Title	Decision	mainreason	Which review?
Accinelli et. al	2015	American journal of respiratory and critical care medicine	Sustained Benefit of Community-based Tuberculosis Interventions after 30 Years	exclude	NA	NA
Ackermann et. al	2018	Euro surveillance : bulletin Europeen sur les maladies transmissibles = European communicable disease bulletin	Screening for infectious diseases among newly arrived asylum seekers, Bavaria, Germany, 2015	exclude	No comparison group	NA
Adams et. al	2017	The international journal of tuberculosis and lung disease : the official journal of the International Union against Tuberculosis and Lung Disease	High completion rates of isoniazid preventive therapy among persons living with HIV in Swaziland	exclude	No comparison group	NA
Adams et. al	2014	The Pediatric infectious disease journal	Diagnosis and treatment of tuberculosis among children at an HIV care program in Dar es Salaam, Tanzania	exclude	No comparison group	NA
Adane et. al	2020	Tuberc Res Treat	Prevalence and Associated Factors of Tuberculosis among Adult Household Contacts of Smear Positive Pulmonary Tuberculosis Patients Treated in Public Health Facilities of Haramaya District, Oromia Region, Eastern Ethiopia	exclude	No comparison group	NA
Adane et. al	2016	PloS one	Half of Pulmonary Tuberculosis Cases Were Left Undiagnosed in Prisons of the Tigray Region of Ethiopia: Implications for Tuberculosis Control	exclude	No comparison group	NA
Adane et. al	2019	Lancet Glob Health	Tuberculosis case detection by trained inmate peer educators in a resource-limited prison setting in Ethiopia: a cluster-randomised trial	include	NA	CNR review
Addis et. al	2015	Asian Pacific journal of tropical medicine	Prevalence of smear positive pulmonary tuberculosis in Gondar prisoners, North West Ethiopia	exclude	No comparison group	NA
Adelman et. al	2015	The international journal of tuberculosis and lung disease : the official journal of the International Union against Tuberculosis and Lung Disease	Intensified tuberculosis case finding among HIV-infected persons using a WHO symptom screen and Xpert((R)) MTB/RIF	exclude	No comparison group	NA
Adesokan et. al	2014	African journal of medicine and medical sciences	Prevalence of previously undetected tuberculosis and underlying risk factors for transmission in a prison setting in Ibadan, south-western Nigeria	exclude	No comparison group	NA

Author	Year	Journal	Title	Decision	mainreason	Which review?
Adesokan et. al	2012	The international journal of tuberculosis and lung disease : the official journal of the International Union against Tuberculosis and Lung Disease	Mycobacterium bovis infection in livestock workers in Ibadan, Nigeria: evidence of occupational exposure	exclude	No comparison group	NA
Adetifa et. al	2016	Bulletin of the World Health Organization	A tuberculosis nationwide prevalence survey in Gambia, 2012	exclude	No comparison group	NA
Adetifa et. al	2017	American Journal of Tropical Medicine and Hygiene	Mycobacterium tuberculosis infection in close childhood contacts of adults with pulmonary tuberculosis is increased by secondhand exposure to tobacco	exclude	No comparison group	NA
Adetunji et. al	2019	J Immunoassay Immunochem	Rifampicin-resistant tuberculosis among known HIV-infected patients in Oyo State, Nigeria	exclude	No comparison group	NA
Adinarayanan et. al	2017	The international journal of tuberculosis and lung disease : the official journal of the International Union against Tuberculosis and Lung Disease	Role of bacille Calmette-Guerin in preventing tuberculous infection	exclude	No comparison group	NA
Adjobimey et. al	2016	International Journal of Tuberculosis and Lung Disease	Implementation of isoniazid preventive therapy in children aged under 5 years exposed to tuberculosis in Benin	exclude	No comparison group	NA
Adler-Shohet et. al	2014	The Pediatric infectious disease journal	Management of latent tuberculosis infection in child contacts of multidrug-resistant tuberculosis	exclude	No comparison group	NA
Adler et. al	2017	The international journal of tuberculosis and lung disease : the official journal of the International Union against Tuberculosis and Lung Disease	Tuberculosis in HIV-infected South African children with complicated severe acute malnutrition	exclude	No comparison group	NA
Agarwal et. al	2018	Gastroenterology	VERY HIGH RATE OF TUBERCULOSIS COMPLICATING INFLIXIMAB THERAPY FOR INFLAMMATORY BOWEL DISEASE DESPITE TUBERCULOSIS SCREENING IN INDIA	exclude	No comparison group	NA
Agaya et. al	2015	Tropical medicine & international health : TM & IH	Tuberculosis and latent tuberculosis infection among healthcare workers in Kisumu, Kenya	exclude	No comparison group	NA
Aggarwal et. al	2015	PLoS ONE	Prevalence of pulmonary tuberculosis among adults in a north Indian district	exclude	No comparison group	NA
Agizew T.B. et. al	2010	International Journal of Tuberculosis and Lung Disease	Tuberculosis in asymptomatic HIV-infected adults with abnormal chest radiographs screened for tuberculosis prevention	exclude	No comparison group	NA

Author	Year	Journal	Title	Decision	mainreason	Which review?
Agizew et. al	2017	PloS one	Higher-than-expected prevalence of non-tuberculous mycobacteria in HIV setting in Botswana: Implications for diagnostic algorithms using Xpert MTB/RIF assay	exclude	No comparison group	NA
Agizew et. al	2019	BMC Infect Dis	Tuberculosis treatment outcomes among people living with HIV diagnosed using Xpert MTB/RIF versus sputum-smear microscopy in Botswana: a stepped-wedge cluster randomised trial	exclude	Healthcare based screening	NA
Aguilera et. al	2016	The international journal of tuberculosis and lung disease : the official journal of the International Union against Tuberculosis and Lung Disease	Tuberculosis in prisoners and their contacts in Chile: estimating incidence and latent infection	exclude	No comparison group	NA
Aguirre et. al	2017	Mem. Inst. Oswaldo Cruz	Prevalence of tuberculosis respiratory symptoms and associated factors in the indigenous populations of Paraguay (2012)	exclude	No comparison group	NA
Ahmad Khan et. al	2014	AIDS (London, England)	Performance of symptom-based tuberculosis screening among people living with HIV: not as great as hoped	exclude	No comparison group	NA
Ahmed et. al	2020	Pediatrics	Interferon-gamma Release Assays in Children <15 Years of Age	exclude	No comparison group	NA
Ahmed et. al	2020	Pediatrics	Interferon-γ Release Assays in Children <15 Years of Age	exclude	No comparison group	NA
Ahmed et. al	2019	Indian J. Public Health Res. Dev.	Epidemiology character of tuberculosis among internally displaced persons in Tikrit City	exclude	No comparison group	NA
Ahmed et. al	2017	International journal of mycobacteriology	Association between pulmonary tuberculosis and Type 2 diabetes in Sudanese patients	exclude	No comparison group	NA
Ahn et. al	2015	American Journal of Infection Control	Nosocomial exposure to active pulmonary tuberculosis in a neonatal intensive care unit	exclude	No comparison group	NA
Aia et. al	2018	Western Pacific surveillance and response journal : WPSAR	Epidemiology of tuberculosis in Papua New Guinea: analysis of case notification and treatment-outcome data, 2008-2016	exclude	No comparison group	NA
Aibana et. al	2016	PloS one	Nutritional Status and Tuberculosis Risk in Adult and Pediatric Household Contacts	exclude	No comparison group	NA
Aibana et. al	2018	The Journal of nutrition	Vitamin E Status Is Inversely Associated with Risk of Incident Tuberculosis Disease among Household Contacts	exclude	No comparison group	NA

Author	Year	Journal	Title	Decision	mainreason	Which review?
Aibana et. al	2017	Clinical infectious diseases : an official publication of the Infectious Diseases Society of America	Impact of Vitamin A and Carotenoids on the Risk of Tuberculosis Progression	exclude	No comparison group	NA
Akanbi et. al	2017	BMC pulmonary medicine	Evaluation of gene xpert for routine diagnosis of HIV-associated tuberculosis in Nigeria: A prospective cohort study	exclude	No comparison group	NA
Akanbi et. al	2013	AIDS research and human retroviruses	Tuberculosis after one year of combination antiretroviral therapy in Nigeria: a retrospective cohort study	exclude	No comparison group	NA
Akkerman et. al	2016	The European respiratory journal	Implementing tuberculosis entry screening for asylum seekers: the Groningen experience	exclude	No comparison group	NA
Aksenova et. al	2020	Int J Infect Dis	Latent tuberculosis infection in children and adolescents in Russia	exclude	ACF in children only	NA
Aksenova et. al	2018	European Respiratory Journal	TB detection in children in Moscow (Russia) as in low TB incidence region	exclude	ACF in children only	NA
Al Hajoj et. al	2016	PloS one	Interferon Gamma Release Assay versus Tuberculin Skin Testing among Healthcare Workers of Highly Diverse Origin in a Moderate Tuberculosis Burden Country	exclude	No comparison group	NA
Al Hosani et. al	2013	Journal of epidemiology and global health	Prevalence of pulmonary tuberculosis among expatriates subjected to medical visa screening in Abu Dhabi, United Arab Emirates	exclude	No comparison group	NA
Al Wakeel et. al	2014	Nephrology Dialysis Transplantation	The use of quantiferon TB gold in-tube test in screening latent and active tuberculosis among saudi dialysis patients	exclude	No comparison group	NA
Al-Darraji et. al	2013	PloS one	The diagnostic performance of a single GeneXpert MTB/RIF assay in an intensified tuberculosis case finding survey among HIV-infected prisoners in Malaysia	exclude	No comparison group	NA
Al-Darraji et. al	2016	Tropical medicine & international health : TM & IH	Undiagnosed pulmonary tuberculosis among prisoners in Malaysia: an overlooked risk for tuberculosis in the community	exclude	No comparison group	NA
Al-Darraji et. al	2015	Respirology	Factors limiting the scale up of isoniazid preventive therapy among hiv-infected prisoners in Malaysia	exclude	No comparison group	NA
Alamo et. al	2012	Tropical medicine & international health : TM & IH	Performance of the new WHO diagnostic algorithm for smear-negative pulmonary tuberculosis in HIV prevalent settings: a multisite study in Uganda	exclude	Healthcare based screening	NA

Author	Year	Journal	Title	Decision	mainreason	Which review?
Alawdah et. al	2017	Open Forum Infectious Diseases	Improving patient and employee safety through implementation of an infection risk screening process for international patients at boston children's hospital-the airship protocol	exclude	No comparison group	NA
Aldridge et. al	2015	BMJ open	Effectiveness of peer educators on the uptake of mobile X-ray tuberculosis screening at homeless hostels: a cluster randomised controlled trial	exclude	NA	NA
Aldridge et. al	2016	The Lancet. Infectious diseases	Prevalence of and risk factors for active tuberculosis in migrants screened before entry to the UK: a population-based cross-sectional study	exclude	No comparison group	NA
Aldridge et. al	2016	Lancet (London, England)	Tuberculosis in migrants moving from high-incidence to low-incidence countries: a population-based cohort study of 519 955 migrants screened before entry to England, Wales, and Northern Ireland	exclude	No comparison group	NA
Alekseev et. al	2018	European Respiratory Journal	The efficacy of screening for tuberculosis infection in paediatric population in the Republic of Tatarstan	exclude	ACF in children only	NA
Alelign et. al	2019	PLoS One	Smear positive tuberculosis and genetic diversity of M. tuberculosis isolates in individuals visiting health facilities in South Gondar Zone, northwest Ethiopia	exclude	No comparison group	NA
Alelign et. al	2019	Tuberc Res Treat	Tuberculosis at Farmer-Cattle Interface in the Rural Villages of South Gondar Zone of Northwest Ethiopia	exclude	No comparison group	NA
Alemayehu et. al	2014	International journal of mycobacteriology	Active tuberculosis case finding and detection of drug resistance among HIV-infected patients: A cross-sectional study in a TB endemic area, Gondar, Northwest Ethiopia	exclude	No comparison group	NA
Alemu et. al	2016	PloS one	High Incidence of Tuberculosis in the Absence of Isoniazid and Cotrimoxazole Preventive Therapy in Children Living with HIV in Northern Ethiopia: A Retrospective Follow-Up Study	exclude	No comparison group	NA
Ali et. al	2019	Transactions of the Royal Society of Tropical Medicine and Hygiene	A descriptive analysis of screening and treatment of tuberculosis in pregnant women in urban tertiary care hospitals in Pakistan	exclude	No comparison group	NA
Ali et. al	2015	PloS one	Prevalence of Pulmonary Tuberculosis among Prison Inmates in Ethiopia, a Cross-Sectional Study	exclude	No comparison group	NA

Author	Year	Journal	Title	Decision	mainreason	Which review?
Almufty et. al	2019	Trop Med Infect Dis	Latent Tuberculosis Infection among Healthcare Workers in Duhok Province: From Screening to Prophylactic Treatment	exclude	No comparison group	NA
Alsayed Hasanain et. al	2019	Trop Med Int Health	Predictors of therapeutic failure among patients with acute brucellosis treated by dual therapy with doxycycline-rifampin	exclude	No relevant data / not an ACF intervention	NA
Alsharif et. al	2020	Ann Thorac Med	Incidence of latent tuberculosis infection among health science students during clinical training	exclude	No comparison group	NA
Alshukairi et. al	2020	J Infect Public Health	Family cluster of multi-drug resistant tuberculosis in Kingdom of Saudi Arabia	exclude	No comparison group	NA
Altet et. al	2015	Annals of the American Thoracic Society	Predicting the Development of Tuberculosis with the Tuberculin Skin Test and QuantiFERON Testing	exclude	No comparison group	NA
Alvarez-Alvarez et. al	2013	Anales de pediatria (Barcelona, Spain : 2003)	[Description of tuberculosis outbreak and usefulness of mediastinal ultrasound]	exclude	No comparison group	NA
Alvarez et. al	2014	PloS one	Taima (stop) TB: the impact of a multifaceted TB awareness and door-to-door campaign in residential areas of high risk for TB in Iqaluit, Nunavut	exclude	Fewer than 1000 people screened	NA
Amanullah et. al	2014	The international journal of tuberculosis and lung disease : the official journal of the International Union against Tuberculosis and Lung Disease	High tuberculosis prevalence in children exposed at home to drug-resistant tuberculosis	exclude	No comparison group	NA
Amare D. et. al	2010	Int J Tuberc Lung Dis	Prevalence of pulmonary tb and hiv among tb suspects in rural community in southwest ethiopia. 41st world conference on lung health of the international union against tuberculosis and lung disease, berlin, germany, 11-15 november 2010	exclude	No comparison group	NA
Aminzadeh et. al	2011	International journal of preventive medicine	A six months follow-up on children less than 6 years old in contact with smear positive tuberculosis patients, varamin city, tehran, iran	exclude	No comparison group	NA
Amiri et. al	2014	PloS one	Vulnerability of homeless people in Tehran, Iran, to HIV, tuberculosis and viral hepatitis	exclude	No comparison group	NA
An and et. al	2018	Cureus	Improving Screening for Latent Tuberculosis Infection in a Student-run Free Clinic	exclude	No comparison group	NA

Author	Year	Journal	Title	Decision	mainreason	Which review?
An der Heiden et. al	2017	Euro surveillance : bulletin Europeen sur les maladies transmissibles = European communicable disease bulletin	Contact investigation after a fatal case of extensively drug-resistant tuberculosis (XDR-TB) in an aircraft, Germany, July 2013	exclude	No comparison group	NA
Anaraki et. al	2018	Epidemiology and infection	Expected background rates of latent TB infection in London inner city schools: lessons from a TB contact investigation exercise in a secondary school	exclude	No comparison group	NA
Andama et. al	2020	Diagn. Microbiol. Infect. Dis.	Accuracy and incremental yield of urine Xpert MTB/RIF Ultra versus Determine TB-LAM for diagnosis of pulmonary tuberculosis	exclude	Healthcare based screening	NA
Andre et. al	2018	Bulletin of the World Health Organization	Patient-led active tuberculosis case-finding in the Democratic Republic of the Congo	exclude	No comparison group	NA
Andrews et. al	2015	American journal of respiratory and critical care medicine	The dynamics of QuantiFERON-TB gold in-tube conversion and reversion in a cohort of South African adolescents	exclude	No comparison group	NA
Andrews et. al	2017	The Lancet. Respiratory medicine	Serial QuantiFERON testing and tuberculosis disease risk among young children: an observational cohort study	exclude	No comparison group	NA
Aneja K.S. et. al	1984	Indian Journal of Tuberculosis	Active case finding in tuberculosis as a component of primary health care	exclude	NA	NA
Anger et. al	2012	Clinical infectious diseases : an official publication of the Infectious Diseases Society of America	Active case finding and prevention of tuberculosis among a cohort of contacts exposed to infectious tuberculosis cases in New York City	exclude	No comparison group	NA
Anigilaje et. al	2016	PloS one	Tuberculosis, before and after Antiretroviral Therapy among HIV-Infected Children in Nigeria: What Are the Risk Factors?	exclude	No comparison group	NA
Anih et. al	2019	Journal of Acquired Immune Deficiency Syndromes	Implementation of tuberculosis service integration into ANC and PMTCT programs in northern nigeria	exclude	No comparison group	NA
Aquino et. al	2015	Cadernos de saude publica	Factors associated with treatment for latent tuberculosis in persons living with HIV/AIDS	exclude	No comparison group	NA
Ar-Karachaiphong et. al	2019	Journal of the Medical Association of Thailand	Agreement of tuberculin skin test and quantiFERON®-TB gold-in-tube for screening Mycobacterium tuberculosis infection in healthcare workers in a university hospital	exclude	Fewer than 1000 people screened	NA
Araujo et. al	2020	Int J Infect Dis	Determinants of losses in the latent tuberculosis cascade of care in Brazil: A retrospective cohort study	exclude	No comparison group	NA

Author	Year	Journal	Title	Decision	mainreason	Which review?
Armstrong-Hough et. al	2017	PloS one	Drop-out from the tuberculosis contact investigation cascade in a routine public health setting in urban Uganda: A prospective, multi-center study	exclude	No comparison group	NA
Arnedo-Pena et. al	2020	Int J Tuberc Lung Dis	Vitamin D status and latent tuberculosis infection: conversion in nursing homes, Spain	exclude	No comparison group	NA
Arnold et. al	2016	The Journal of infection	XDR-TB transmission in London: Case management and contact tracing investigation assisted by early whole genome sequencing	exclude	Fewer than 1000 people screened	NA
Arroyave et. al	2017	Epidemiology and infection	Negative latent tuberculosis at time of incarceration: identifying a very high-risk group for infection	exclude	No comparison group	NA
Arroyave et. al	2017	Epidemiology and infection	Negative latent tuberculosis at time of incarceration: identifying a very high-risk group for infection	exclude	No comparison group	NA
Arroyave et. al	2019	J Immigr Minor Health	Guards in Prisons: A Risk Group for Latent Tuberculosis Infection	exclude	No comparison group	NA
Arscott-Mills et. al	2014	Journal of tropical pediatrics	Yield of screening for TB and HIV among children failing to thrive in Botswana	exclude	Healthcare based screening	NA
Asemahagn et. al	2017	Tuberculosis research and treatment	Are Shopkeepers Suffering from Pulmonary Tuberculosis in Bahir Dar City, Northwest Ethiopia: A Cross-Sectional Survey	exclude	No comparison group	NA
Assefa et. al	2019	BMC Infectious Diseases	Missed pulmonary tuberculosis: A cross sectional study in the general medical inpatient wards of a large referral hospital in Ethiopia 11 Medical and Health Sciences 1117 Public Health and Health Services Julian Tang	exclude	No comparison group	NA
Attah et. al	2018	Alexandria Journal of Medicine	Risk factors associated with paediatric tuberculosis in an endemic setting	exclude	No comparison group	NA
Auld et. al	2020	BMC Med	Effect of tuberculosis screening and retention interventions on early antiretroviral therapy mortality in Botswana: a stepped-wedge cluster randomized trial	exclude	Healthcare based screening	NA
Auld et. al	2016	PloS one	Wide Variations in Compliance with Tuberculosis Screening Guidelines and Tuberculosis Incidence between Antiretroviral Therapy Facilities - Cote d'Ivoire	exclude	No comparison group	NA
Aunsborg et. al	2020	Int J Infect Dis	A clinical score has utility in tuberculosis case-finding among patients with HIV: A feasibility study from Bissau	exclude	No relevant data / not an ACF intervention	NA

Author	Year	Journal	Title	Decision	mainreason	Which review?
Aye et. al	2018	International journal of infectious diseases : IJID : official publication of the International Society for Infectious Diseases	Evaluation of a tuberculosis active case finding project in peri-urban areas, Myanmar: 2014-2016	include	NA	CNR review
Aye et. al	2018	International journal of infectious diseases : IJID : official publication of the International Society for Infectious Diseases	Evaluation of a tuberculosis active case finding project in peri-urban areas, Myanmar: 2014-2016	include but duplicate	NA	NA
Ayles H. et. al	2012	19th Conference of Retroviruses and Opportunistic Infections	A household-based hiv and tb intervention increases hiv testing in households and reduces prevalence of tb at the community level: The zamstar community randomized trial	include but duplicate	NA	NA
Ayles et. al	2013	Lancet (London, England)	Effect of household and community interventions on the burden of tuberculosis in southern Africa: the ZAMSTAR community-randomised trial	include	NA	Prevelance review
Azit et. al	2019	BMC Public Health	Factors associated with tuberculosis disease among children who are household contacts of tuberculosis cases in an urban setting in Malaysia	exclude	No comparison group	NA
Bacha et. al	2019	Journal of the International AIDS Society	Actively contributing to a cascade of change: Analysis of the TB treatment cascade among children and adolescents living with HIV in six high TB/HIV burden countries	exclude	No comparison group	NA
Baghaei et. al	2018	The clinical respiratory journal	Diagnosing active and latent tuberculosis among Iranian HIV-infected patients	exclude	No comparison group	NA
Baghaie et. al	2012	Eastern Mediterranean health journal = La revue de sante de la Mediterranee orientale = al-Majallah al-sihhiyah li-sharq al-mutawassit	Contact tracing of a 15-year-old girl with smear-negative pulmonary tuberculosis in Tehran	exclude	No comparison group	NA
Bah et. al	2012	Revue de Medecine Legale	Prevalence of tuberculosis in the prison population of Conakry, Guinea Republic	exclude	No comparison group	NA
Bailey et. al	2016	BMC infectious diseases	The association of hyperglycaemia with prevalent tuberculosis: a population-based cross-sectional study	exclude	No comparison group	NA
Bajema et. al	2019	BMC infectious diseases	Subclinical tuberculosis among adults with HIV: clinical features and outcomes in a South African cohort	exclude	No comparison group	NA

Author	Year	Journal	Title	Decision	mainreason	Which review?
Bakeera-Kitaka et. al	2011	The international journal of tuberculosis and lung disease : the official journal of the International Union against Tuberculosis and Lung Disease	Tuberculosis in human immunodeficiency virus infected Ugandan children starting on antiretroviral therapy	exclude	ACF in children only	NA
Bakeera-Kitaka et. al	2011	The international journal of tuberculosis and lung disease : the official journal of the International Union against Tuberculosis and Lung Disease	Tuberculosis in human immunodeficiency virus infected Ugandan children starting on antiretroviral therapy	exclude	Healthcare based screening	NA
Balasubramanian R. et. al	2004	International Journal of Tuberculosis and Lung Disease	Gender disparities in tuberculosis: Report from a rural DOTS programme in south India	exclude	No comparison group	NA
Balcells et. al	2016	The international journal of tuberculosis and lung disease : the official journal of the International Union against Tuberculosis and Lung Disease	M. tuberculosis DNA detection in nasopharyngeal mucosa can precede tuberculosis development in contacts	exclude	No comparison group	NA
Balcells et. al	2016	The international journal of tuberculosis and lung disease : the official journal of the International Union against Tuberculosis and Lung Disease	M. tuberculosis DNA detection in nasopharyngeal mucosa can precede tuberculosis development in contacts	exclude	No comparison group	NA
Balcha et. al	2015	Global health action	Outcome of tuberculosis treatment in HIV-positive adults diagnosed through active versus passive case-finding	exclude	Fewer than 1000 people screened	NA
Balcha et. al	2014	PloS one	Intensified tuberculosis case-finding in HIV-positive adults managed at Ethiopian health centers: diagnostic yield of Xpert MTB/RIF compared with smear microscopy and liquid culture	exclude	No comparison group	NA
Baldassari et. al	2019	Mult Scler J Exp Transl Clin	Tuberculosis screening in multiple sclerosis: effect of disease-modifying therapies and lymphopenia on the prevalence of indeterminate TB screening results in the clinical setting	exclude	No comparison group	NA
Baliashvili et. al	2018	Public health action	A population-based tuberculosis contact investigation in the country of Georgia	exclude	No comparison group	NA
Balmelli et. al	2014	Swiss medical weekly	Contact tracing investigation after professional exposure to tuberculosis in a Swiss hospital using both tuberculin skin test and IGRA	exclude	No comparison group	NA

Author	Year	Journal	Title	Decision	mainreason	Which review?
Banfield et. al	2012	PloS one	Factors associated with the performance of a blood-based interferon-gamma release assay in diagnosing tuberculosis	exclude	No comparison group	NA
Banfield et. al	2012	PLoS ONE	Factors associated with the performance of a blood-based interferon-?_ release assay in diagnosing tuberculosis	exclude	No comparison group	NA
Banjara et. al	2015	Transactions of the Royal Society of Tropical Medicine and Hygiene	Feasibility of a combined camp approach for vector control together with active case detection of visceral leishmaniasis, post kala-azar dermal leishmaniasis, tuberculosis, leprosy and malaria in Bangladesh, India and Nepal: an exploratory study	exclude	No comparison group	NA
Banu et. al	2013	PloS one	Epidemiology of tuberculosis in an urban slum of Dhaka City, Bangladesh	exclude	No comparison group	NA
Banu et. al	2015	PloS one	Effect of active case finding on prevalence and transmission of pulmonary tuberculosis in Dhaka Central Jail, Bangladesh	exclude	No comparison group	NA
Barcellini et. al	2019	PLoS One	App-based symptoms screening with Xpert MTB/RIF Ultra assay used for active tuberculosis detection in migrants at point of arrivals in Italy: The E-DETECT TB intervention analysis	exclude	NA	NA
Basham et. al	2019	Can J Public Health	Tuberculosis among northern Manitoba First Nations, 2008-2012: program performance on- and off-reserve	exclude	No comparison group	NA
Basir et. al	2019	BMC Health Serv Res	Operationalization of bi-directional screening for tuberculosis and diabetes in private sector healthcare clinics in Karachi, Pakistan	exclude	Healthcare based screening	NA
Bassett et. al	2019	BMC infectious diseases	Test and Treat TB: a pilot trial of GeneXpert MTB/RIF screening on a mobile HIV testing unit in South Africa	exclude	No comparison group	NA
Bates et. al	2012	PloS one	Evaluation of the burden of unsuspected pulmonary tuberculosis and co-morbidity with non-communicable diseases in sputum producing adult inpatients	exclude	No comparison group	NA
Batra et. al	2012	PloS one	Childhood tuberculosis in household contacts of newly diagnosed TB patients	exclude	No comparison group	NA
Becerra et. al	2011	Lancet (London, England)	Tuberculosis burden in households of patients with multidrug-resistant and extensively drug-resistant tuberculosis: a retrospective cohort study	exclude	No comparison group	NA

Author	Year	Journal	Title	Decision	mainreason	Which review?
Becerra et. al	2013	The Pediatric infectious disease journal	Tuberculosis in children exposed at home to multidrug-resistant tuberculosis	exclude	No comparison group	NA
Becerra et. al	2019	Bmj	Transmissibility and potential for disease progression of drug resistant Mycobacterium tuberculosis: prospective cohort study	exclude	No comparison group	NA
Bedell et. al	2012	PloS one	High prevalence of tuberculosis and serious bloodstream infections in ambulatory individuals presenting for antiretroviral therapy in Malawi	exclude	No comparison group	NA
Bedoya et. al	2015	Iatreia	Study and clinical management of child household contacts of tuberculosis patients, medellin 2010-2011	exclude	No comparison group	NA
Bekken et. al	2020	BMC Infect Dis	Identification of subclinical tuberculosis in household contacts using exposure scores and contact investigations	exclude	No comparison group	NA
Bekker et. al	2012	The international journal of tuberculosis and lung disease : the official journal of the International Union against Tuberculosis and Lung Disease	High tuberculosis exposure among neonates in a high tuberculosis and human immunodeficiency virus burden setting	exclude	No comparison group	NA
Belizario et. al	2014	Pathogens and global health	Integrated surveillance of pulmonary tuberculosis and paragonimiasis in Zamboanga del Norte, the Philippines	exclude	No comparison group	NA
Benjamin et. al	2019	PLoS One	Accuracy of Determine TB-LAM Ag to detect TB in HIV infected patients associated with diagnostic methods used in Brazilian public health units	exclude	No comparison group	NA
Benjumea-Bedoya et. al	2019	Front Public Health	Integrated Care for Latent Tuberculosis Infection (LTBI) at a Primary Health Care Facility for Refugees in Winnipeg, Canada: A Mixed-Methods Evaluation	exclude	No comparison group	NA
Bennet et. al	2017	Infectious diseases (London, England)	Tuberculosis infection and disease in the 2015 cohort of unaccompanied minors seeking asylum in Northern Stockholm, Sweden	exclude	No comparison group	NA
Bennet et. al	2019	Pediatr Infect Dis J	Effective Tuberculosis Contact Investigation Using Interferon-Gamma Release Assays	exclude	No comparison group	NA
Berhane et. al	2019	Clin Lab	The Role of Neutrophil to Lymphocyte Count Ratio in the Differential Diagnosis of Pulmonary Tuberculosis and Bacterial Community-Acquired Pneumonia: a Cross-Sectional Study at Ayder and Mekelle Hospitals, Ethiopia	exclude	No comparison group	NA

Author	Year	Journal	Title	Decision	mainreason	Which review?
Berhe et. al	2013	BMC infectious diseases	Population-based prevalence survey of tuberculosis in the Tigray region of Ethiopia	exclude	No comparison group	NA
Berju et. al	2019	Int J Microbiol	Smear-Positive Tuberculosis Prevalence and Associated Factors among Pregnant Women Attending Antinatal Care in North Gondar Zone Hospitals, Ethiopia	exclude	No comparison group	NA
Berkowitz et. al	2018	Diabetes research and clinical practice	The prevalence and determinants of active tuberculosis among diabetes patients in Cape Town, South Africa, a high HIV/TB burden setting	exclude	No comparison group	NA
Berraies et. al	2016	Revue de pneumologie clinique	[Results of tuberculosis screening in children with household contact]	exclude	No comparison group	NA
Bettelli et. al	2019	Haematologica	Latent tuberculosis infection in adults with acute leukemia and aplastic anemia: A retrospective single center experience	exclude	No comparison group	NA
Beyanga et. al	2018	BMC infectious diseases	Investigation of household contacts of pulmonary tuberculosis patients increases case detection in Mwanza City, Tanzania	exclude	No comparison group	NA
Bharara et. al	2019	Sexually Transmitted Infections	Integration of HIV testing with tuberculosis and sexually transmitted infections at a tertiary care hospital in Delhi	exclude	No comparison group	NA
Bhat et. al	2013	PloS one	Intensified tuberculosis case finding among malnourished children in nutritional rehabilitation centres of Karnataka, India: missed opportunities	exclude	No comparison group	NA
Bhatnagar et. al	2019	PLoS One	Intensified tuberculosis and HIV surveillance in a prison in Northeast India: Implementation research	exclude	Fewer than 1000 people screened	NA
Bhatti et. al	2014	Medical Channel	Predisposing factors of HIV and its co-infection with Tuberculosis in the metropolitan city of Karachi	exclude	Fewer than 1000 people screened	NA
Bhatti et. al	2014	Medical Channel	Predisposing factors of HIV and its co-infection with Tuberculosis in the metropolitan city of Karachi	exclude	No comparison group	NA
Bigogo et. al	2018	BMC infectious diseases	Tuberculosis case finding using population-based disease surveillance platforms in urban and rural Kenya	exclude	No comparison group	NA
Binepal et. al	2015	Public health action	Screening difficult-to-reach populations for tuberculosis using a mobile medical unit, Punjab India	exclude	No comparison group	NA

Author	Year	Journal	Title	Decision	mainreason	Which review?
Bjerregaard-Andersen M. et. al	2010	BMC Infectious Diseases	Tuberculosis burden in an urban population: A cross sectional tuberculosis survey from Guinea Bissau	exclude	No comparison group	NA
Bjerrum et. al	2015	BMC infectious diseases	Diagnostic accuracy of the rapid urine lipoarabinomannan test for pulmonary tuberculosis among HIV-infected adults in Ghana-findings from the DETECT HIV-TB study	exclude	No comparison group	NA
Bjerrum et. al	2016	Tropical medicine & international health : TM & IH	Tuberculosis and non-tuberculous mycobacteria among HIV-infected individuals in Ghana	exclude	No comparison group	NA
Bloss et. al	2012	The international journal of tuberculosis and lung disease : the official journal of the International Union against Tuberculosis and Lung Disease	Lessons learned during tuberculosis screening in public medical clinics in Francistown, Botswana	exclude	No comparison group	NA
Blount et. al	2016	BMC public health	Tuberculosis progression rates in U.S. Immigrants following screening with interferon-gamma release assays	exclude	No comparison group	NA
Bobbio et. al	2019	BMJ Open	Focused ultrasound to diagnose HIV-associated tuberculosis (FASH) in the extremely resource-limited setting of South Sudan: a cross-sectional study	exclude	No comparison group	NA
Bodena et. al	2019	Risk Manag Healthc Policy	Trend Analysis And Seasonality Of Tuberculosis Among Patients At The Hiwot Fana Specialized University Hospital, Eastern Ethiopia: A Retrospective Study	exclude	No comparison group	NA
Bogorodskaya et. al	2018	European Respiratory Journal	Results of TB prophylactics and early detection in HIV-positive people in Moscow, Russia	exclude	Healthcare based screening	NA
Bonnet et. al	2017	The international journal of tuberculosis and lung disease : the official journal of the International Union against Tuberculosis and Lung Disease	Prospective cohort study of the feasibility and yield of household child tuberculosis contact screening in Uganda	exclude	No comparison group	NA
Bonsu et. al	2020	Int J Tuberc Lung Dis	National population-based tuberculosis prevalence survey in Ghana, 2013	exclude	No comparison group	NA
Bonvicini et. al	2018	International journal of environmental research and public health	Compliance with Tuberculosis Screening in Irregular Immigrants	exclude	No comparison group	NA
Bonvicini et. al	2019	International Journal of Environmental Research and Public Health	Compliance with tuberculosis screening in irregular immigrants	exclude	No comparison group	NA

Author	Year	Journal	Title	Decision	mainreason	Which review?
Borgdorff M.W. et. al	2004	Emerging Infectious Diseases	New measurable indicator for tuberculosis case detection	exclude	No comparison group	NA
Borraccino et. al	2014	The Journal of infection	Yield of tuberculosis contact investigation in a low-incidence country	exclude	No comparison group	NA
Borroto Guti?rrez et. al	2015	Rev. Cuba. Med. Trop.	Tuberculosis risk in the staff of three clinical surgical hospitals at Havana city	exclude	No comparison group	NA
Borroto et. al	2019	Transactions of the Royal Society of Tropical Medicine and Hygiene	Latent tuberculosis infection in health care workers of Cuban health facilities: Risk assessing and results of an intervention	exclude	No comparison group	NA
Bosa et. al	2017	Mediterranean journal of hematology and infectious diseases	Feasibility and Effectiveness of Tuberculosis Active Case-Finding among Children Living with Tuberculosis Relatives: a Cross-Sectional Study in Guinea-Bissau	exclude	No comparison group	NA
Bourgarit et. al	2015	Annals of the American Thoracic Society	Latent Tuberculosis Infection Screening and 2-Year Outcome in Antiretroviral-Naive HIV-Infected Patients in a Low-Prevalence Country	exclude	No comparison group	NA
Bua et. al	2016	Journal of public health (Oxford, England)	Tuberculosis screening among asylum seekers in Sardinia	exclude	No comparison group	NA
Bunyasi et. al	2019	Int J Tuberc Lung Dis	Temporal trends in the prevalence of Mycobacterium tuberculosis infection in South African adolescents	exclude	No comparison group	NA
Buonsenso et. al	2020	J Clin Microbiol	Accuracy of QuantiFERON-TB Gold-PLUS Test for the Diagnosis of Mycobacterium tuberculosis infection in Children	exclude	No comparison group	NA
Busatto et. al	2017	Revista brasileira de enfermagem	Tuberculosis among prison staff in Rio Grande do Sul	exclude	No comparison group	NA
Butt et. al	2013	Journal of Pakistan Association of Dermatologists	Frequency of pulmonary tuberculosis in patients with skin diseases requiring high dose long-term systemic steroid therapy	exclude	No comparison group	NA
Bwana et. al	2011	Tanzania journal of health research	Smear positive pulmonary tuberculosis among HIV patients receiving highly active antiretroviral therapy in Dar es Salaam, Tanzania	exclude	No comparison group	NA
Byashalira et. al	2019	Int J Mycobacteriol	Clinical outcomes of new algorithm for diagnosis and treatment of Tuberculosis sepsis in HIV patients	exclude	Healthcare based screening	NA
Cadmus et. al	2018	Journal of preventive medicine and hygiene	Isolation of Mycobacterium tuberculosis from livestock workers and implications for zooanthroponotic transmission in Ibadan, South-western Nigeria	exclude	No comparison group	NA

Author	Year	Journal	Title	Decision	mainreason	Which review?
Calligaro et. al	2017	The Lancet. Infectious diseases	Effect of new tuberculosis diagnostic technologies on community-based intensified case finding: a multicentre randomised controlled trial	exclude	NA	NA
Camargos et. al	2019	Pediatric Pulmonology	Agreement between tuberculin skin test and interferon-gamma release assay for the diagnosis of latent tb infection among under fifteen-year-olds	exclude	No comparison group	NA
Camelique et. al	2019	Int J Tuberc Lung Dis	Mobile community-based active case-finding for tuberculosis among older populations in rural Cambodia	exclude	No comparison group	NA
Campbell et. al	2019	American journal of kidney diseases : the official journal of the National Kidney Foundation	Screening for Latent Tuberculosis Infection in Migrants With CKD: A Cost-effectiveness Analysis	exclude	No comparison group	NA
Cao et. al	2019	BMC Infect Dis	The association between tuberculin skin test result and active tuberculosis risk of college students in Beijing, China: a retrospective cohort study	exclude	No comparison group	NA
Capewell S. et. al	1986	Tubercle	The diagnosis and management of tuberculosis in common hostel dwellers	exclude	NA	NA
Capewell S. et. al	1984	British Journal of Diseases of the Chest	The value of contact procedures for tuberculosis in Edinburgh	exclude	No comparison group	NA
Carbone Ada et. al	2015	BMC infectious diseases	Active and latent tuberculosis in Brazilian correctional facilities: a cross-sectional study	exclude	No comparison group	NA
Carrizales-Luna et. al	2019	Annals of the Rheumatic Diseases	Quantiferon gold-plus and tuberculin skin test reactivity predictors in patients with rheumatoid arthritis	exclude	No comparison group	NA
Casas et. al	2011	Tropical medicine & international health : TM & IH	Burden and outcome of HIV infection and other morbidities in health care workers attending an Occupational Health Program at the Provincial Hospital of Tete, Mozambique	exclude	No comparison group	NA
Cassels A. et. al	1982	Tubercle	Tuberculosis case-finding in Eastern Nepal	exclude	No comparison group	NA
Castells Carrillo et. al	2019	Enferm Infecc Microbiol Clin	Diagnostic delay as main contributing factor to a large outbreak of tuberculosis in a university	exclude	No comparison group	NA
Cates et. al	2016	Journal of public health management and practice : JPHMP	Contact Investigations Around Mycobacterium tuberculosis Patients Without Positive Respiratory Culture	exclude	Fewer than 1000 people screened	NA
Cavalcante S.C. et. al	2010	International Journal of Tuberculosis and Lung Disease	Community-randomized trial of enhanced DOTS for tuberculosis control in Rio de Janeiro, Brazil	exclude	Contact tracing	NA

Author	Year	Journal	Title	Decision	mainreason	Which review?
Cegielski et. al	2013	Revista panamericana de salud publica = Pan American journal of public health	[Eliminating tuberculosis one neighborhood at a time]	include	NA	CNR review
Ch and ra et. al	2018	Indian Journal of Tuberculosis	Tuberculosis and other chronic morbidity profile of sewage workers of Delhi	exclude	No comparison group	NA
Chadha et. al	2019	PLoS One	Sub-national TB prevalence surveys in India, 2006-2012: Results of uniformly conducted data analysis	exclude	No comparison group	NA
Chadha et. al	2019	Indian J Tuberc	Sensitivity and specificity of screening tools and smear microscopy in active tuberculosis case finding	exclude	No relevant data / not an ACF intervention	NA
Chadha et. al	2012	PloS one	Prevalence of pulmonary tuberculosis among adults in a rural sub-district of South India	exclude	No comparison group	NA
Chan et. al	2019	Southeast Asian J. Trop. Med. Public Health	A cross sectional survey of pulmonary tuberculosis among elderly diabetics attending primary care clinics in Penang, Malaysia	exclude	No comparison group	NA
Chancellor et. al	2019	N Z Med J	Infectious pulmonary tuberculosis in a New Zealand cancer centre	exclude	No comparison group	NA
Chandra et. al	2019	Indian J Tuberc	Tuberculosis and other chronic morbidity profile of sewage workers of Delhi	exclude	No comparison group	NA
Chandrasekaran et. al	2016	Open Forum Infectious Diseases	Household contact tracing of adult pulmonary tuberculosis (TB) patients in India: Prevalence of TB disease and infection	exclude	No comparison group	NA
Charles et. al	2016	PLoS ONE	Implementation of tuberculosis intensive case finding, isoniazid preventive therapy, and infection control ("Three I's") and HIV-tuberculosis service integration in lower income countries	exclude	Healthcare based screening	NA
Charoensook et. al	2018	Journal of Infection in Developing Countries	Pulmonary tuberculosis screening and quality of life among migrant workers, Northern Thailand	exclude	No comparison group	NA
Chatla et. al	2018	The Indian journal of tuberculosis	Active case finding of rifampicin sensitive and resistant TB among household contacts of drug resistant TB patients in Andhra Pradesh and Telangana states of India - A systematic screening intervention	exclude	No comparison group	NA
Chatterjee et. al	2014	PloS one	Incidence of active pulmonary tuberculosis in patients with coincident filarial and/or intestinal helminth infections followed longitudinally in South India	include	No comparison group	Prevelance review

Author	Year	Journal	Title	Decision	mainreason	Which review?
Chauhan et. al	2013	Indian journal of pediatrics	Tuberculin Skin Test, chest radiography and contact screening in children </=5 y: relevance in Revised National Tuberculosis Control Programme (RNTCP)	exclude	No comparison group	NA
Chemeda et. al	2019	J. Clin. Tuberc. Other Microbact. Dis.	Utility of urine as a clinical specimen for the diagnosis of pulmonary tuberculosis in people living with HIV in Addis Ababa, Ethiopia	exclude	No comparison group	NA
Chen et. al	2017	The international journal of tuberculosis and lung disease : the official journal of the International Union against Tuberculosis and Lung Disease	Community-based active case finding for tuberculosis in rural western China: a cross-sectional study	exclude	NA	NA
Chen et. al	2019	Infect Dis Poverty	Role of community-based active case finding in screening tuberculosis in Yunnan province of China	include	NA	CNR review
Chen et. al	2012	The Journal of international medical research	A tuberculosis outbreak among senior high school students in China in 2011	exclude	No comparison group	NA
Cheng et. al	2020	Infect Dis Poverty	Incidence and risk factors of tuberculosis among the elderly population in China: a prospective cohort study	exclude	No comparison group	NA
Cheng et. al	2018	Journal of occupational medicine and toxicology (London, England)	Evaluating a framework for tuberculosis screening among healthcare workers in clinical settings, Inner Mongolia, China	exclude	No comparison group	NA
Cheong et. al	2017	International Journal of Antimicrobial Agents	Prevalence and epidemiologic characteristics of latent tuberculosis infection among healthcare workers at a hospital in Seoul, South Korea	exclude	No comparison group	NA
Chheng et. al	2015	Clinical epidemiology	Tuberculosis case finding in first-degree relative contacts not living with index tuberculosis cases in Kampala, Uganda	exclude	No comparison group	NA
Chiappini et. al	2018	Acta paediatrica (Oslo, Norway : 1992)	Italian multicentre study found infectious and vaccine-preventable diseases in children adopted from Africa and recommends prompt medical screening	exclude	No comparison group	NA
Chinnakali et. al	2016	Annals of Tropical Medicine and Public Health	Active screening for tuberculosis among slum dwellers in selected urban slums of Puducherry, South India	exclude	No comparison group	NA
Chisti et. al	2014	PloS one	A prospective study of the prevalence of tuberculosis and bacteraemia in Bangladeshi children with severe malnutrition and pneumonia including an evaluation of Xpert MTB/RIF assay	exclude	No comparison group	NA

Author	Year	Journal	Title	Decision	mainreason	Which review?
Chopra et. al	2019	Indian Journal of Tuberculosis	Cough of more than two weeks – Time to think beyond pulmonary TB	exclude	No comparison group	NA
Choun et. al	2019	Glob Health Action	Performance of algorithms for tuberculosis active case finding in underserved high-prevalence settings in Cambodia: a cross-sectional study	exclude	No comparison group	NA
Churchyard G.J. et. al	2000	International Journal of Tuberculosis and Lung Disease	Factors associated with an increased case-fatality rate in HIV-infected and non-infected South African gold miners with pulmonary tuberculosis	exclude	No relevant data / not an ACF intervention	NA
Churchyard et. al	2011	Thorax	Twelve-monthly versus six-monthly radiological screening for active case-finding of tuberculosis: a randomised controlled trial	include	NA	CNR review
Churchyard et. al	2010	AIDS (London, England)	Symptom and chest radiographic screening for infectious tuberculosis prior to starting isoniazid preventive therapy: yield and proportion missed at screening	exclude	No relevant data / not an ACF intervention	NA
Coffman et. al	2017	BMC public health	Tuberculosis among older adults in Zambia: burden and characteristics among a neglected group	exclude	No comparison group	NA
Coit et. al	2019	International Journal of Tuberculosis and Lung Disease	Performance of a household tuberculosis exposure survey among children in a Latin American setting	exclude	No comparison group	NA
Colgan et. al	2019	J Paediatr Child Health	Latent tuberculosis may be missed by current screening practices: Analysis of interferon-gamma release assay results from a paediatric refugee clinic	exclude	No comparison group	NA
Collins et. al	2019	Am J Public Health	QuantiFERON-TB Gold Versus Tuberculin Screening and Care Retention Among Persons Experiencing Homelessness: Georgia, 2015-2017	exclude	NA	NA
Coppeta et. al	2019	Open Respir Med J	Prevalence and Risk Factors for Latent Tuberculosis Infection among Healthcare Workers in a Low Incidence Country	exclude	No comparison group	NA
Corbett E.L. et. al	2009	International Journal of Tuberculosis and Lung Disease	Prevalent infectious tuberculosis in Harare, Zimbabwe: Burden, risk factors and implications for control	exclude	No comparison group	NA
Corbett et. al	2010	The Lancet	Comparison of two active case-finding strategies for community-based diagnosis of symptomatic smear-positive tuberculosis and control of infectious tuberculosis in Harare, Zimbabwe (DETECTB): A cluster-randomised trial	include	NA	Both CNR and prevelance

Author	Year	Journal	Title	Decision	mainreason	Which review?
Costa et. al	2011	Jornal brasileiro de pneumologia : publicacao oficial da Sociedade Brasileira de Pneumologia e Tisilogia	Active tuberculosis among health care workers in Portugal	exclude	No comparison group	NA
Costa et. al	2010	Revista Portuguesa de Pneumologia	Comparison of interferon-Î³ release assay and tuberculin test for screening in healthcare workers	exclude	No comparison group	NA
Costa et. al	2019	European Respiratory Journal	Tuberculosis screening at social solidarity institutions	exclude	No comparison group	NA
Costenaro et. al	2016	Journal of acquired immune deficiency syndromes (1999)	Implementation and Operational Research: Implementation of the WHO 2011 Recommendations for Isoniazid Preventive Therapy (IPT) in Children Living With HIV/AIDS: A Ugandan Experience	exclude	No comparison group	NA
Cowger et. al	2017	Journal of acquired immune deficiency syndromes (1999)	Programmatic Evaluation of an Algorithm for Intensified Tuberculosis Case Finding and Isoniazid Preventive Therapy for People Living With HIV in Thailand and Vietnam	exclude	No comparison group	NA
Crampin et. al	2011	Tropical medicine & international health : TM & IH	Married to M. tuberculosis: risk of infection and disease in spouses of smear-positive tuberculosis patients	exclude	No comparison group	NA
Cranmer et. al	2017	The international journal of tuberculosis and lung disease : the official journal of the International Union against Tuberculosis and Lung Disease	Integrating tuberculosis screening in Kenyan Prevention of Mother-To-Child Transmission programs	exclude	No comparison group	NA
Crawshaw et. al	2018	BMC medicine	Infectious disease testing of UK-bound refugees: a population-based, cross-sectional study	exclude	No comparison group	NA
Crepet et. al	2016	International health	Lessons learnt from TB screening in closed immigration centres in Italy	exclude	No comparison group	NA
Creswell et. al	2014	PloS one	An evaluation of systematic tuberculosis screening at private facilities in Karachi, Pakistan	exclude	No comparison group	NA
Cuomo et. al	2018	Journal of infection and public health	Migration and health: A retrospective study about the prevalence of HBV, HIV, HCV, tuberculosis and syphilis infections amongst newly arrived migrants screened at the Infectious Diseases Unit of Modena, Italy	exclude	No comparison group	NA
Cuomo et. al	2019	Journal of Infection and Public Health	Migration and health: A retrospective study about the prevalence of HBV, HIV, HCV, tuberculosis and syphilis infections amongst newly arrived migrants screened at the Infectious Diseases Unit of Modena, Italy	exclude	No comparison group	NA

Author	Year	Journal	Title	Decision	mainreason	Which review?
Da Costa et. al	2010	Revista Portuguesa de Pneumologia	Tuberculosis - Risk of continued transmission in healthcare workers	exclude	No comparison group	NA
Dahiwale et. al	2011	Indian pediatrics	Significance of family survey of index case for detection of tuberculosis	exclude	No comparison group	NA
Datiko D.G. et. al	2009	PLoS ONE	Health extension workers improve tuberculosis case detection and treatment success in southern Ethiopia: A community randomized trial	include	NA	CNR review
Datiko et. al	2017	BMJ global health	Health extension workers improve tuberculosis case finding and treatment outcome in Ethiopia: a large-scale implementation study	include	NA	CNR review
Datiko et. al	2017	The international journal of tuberculosis and lung disease : the official journal of the International Union against Tuberculosis and Lung Disease	A community-based isoniazid preventive therapy for the prevention of childhood tuberculosis in Ethiopia	exclude	No comparison group	NA
Davarpanah et. al	2015	Galen Medical Journal	Incidence of active tuberculosis among human immunodeficiency virus (HIV)-positive patients and evaluation of their responses to usual anti-tuberculosis medications in shiraz, south west of Iran	exclude	No comparison group	NA
Davis et. al	2019	American Journal of Respiratory and Critical Care Medicine	Diagnostic accuracy of c-reactive protein for active TB in adults without HIV: A cross-sectional study	exclude	No comparison group	NA
Davis et. al	2019	ERJ open research	Home-based tuberculosis contact investigation in uganda: a household randomised trial	exclude	Fewer than 1000 people screened	NA
De Francisco et. al	2019	Journal of Crohn's and Colitis	Risk of tuberculosis in patients with inflammatory bowel disease receiving biologics using two interferon-γ release assays as monitoring	exclude	No comparison group	NA
de Glanville et. al	2019	Sci Rep	Household socio-economic position and individual infectious disease risk in rural Kenya	exclude	No comparison group	NA
De Vries G. et. al	2007	American Journal of Respiratory and Critical Care Medicine	Impact of mobile radiographic screening on tuberculosis among drug users and homeless persons	include	NA	CNR review
Debulpaep et. al	2019	Front Pediatr	Contribution of QuantiFERON-TB Gold-in-Tube to the Diagnosis of Mycobacterium tuberculosis Infection in Young Children in a Low TB Prevalence Country	exclude	No comparison group	NA
Debulpaep et. al	2020	Front Pediatr	Tuberculosis Transmission in a Primary School and a Private Language School. An Estimation of Infectivity	exclude	No comparison group	NA

Author	Year	Journal	Title	Decision	mainreason	Which review?
Deery et. al	2014	The international journal of tuberculosis and lung disease : the official journal of the International Union against Tuberculosis and Lung Disease	A home tracing program for contacts of people with tuberculosis or HIV and patients lost to care	exclude	No comparison group	NA
Degner et. al	2016	Journal of correctional health care : the official journal of the National Commission on Correctional Health Care	Comparison of Digital Chest Radiography to Purified Protein Derivative for Screening of Tuberculosis in Newly Admitted Inmates	include	NA	CNR review
Degner et. al	2016	Journal of correctional health care : the official journal of the National Commission on Correctional Health Care	Comparison of Digital Chest Radiography to Purified Protein Derivative for Screening of Tuberculosis in Newly Admitted Inmates	include but duplicate	NA	NA
Del Portillo-Mustieles et. al	2013	Tuberculosis research and treatment	Active Case Finding of Pulmonary Tuberculosis through Screening of Respiratory Symptomatics Using Sputum Microscopy: Is It Time to Change the Paradigm?	exclude	No comparison group	NA
Delva et. al	2016	Tuberculosis research and treatment	Active Tuberculosis Case Finding in Port-au-Prince, Haiti: Experiences, Results, and Implications for Tuberculosis Control Programs	include	NA	CNR review
Den boon S. et. al	2008	Epidemiology and Infection	Comparison of symptoms and treatment outcomes between actively and passively detected tuberculosis cases: The additional value of active case finding	exclude	No comparison group	NA
Deng et. al	2019	Epidemiol Infect	Isolation measures and protection awareness are significant for latent tuberculosis infection: a cross-sectional study based on T-SPOT.TB among health care workers in China	exclude	No comparison group	NA
Deribew et. al	2011	PloS one	Investigation outcomes of tuberculosis suspects in the health centers of Addis Ababa, Ethiopia	exclude	No comparison group	NA
Derseh et. al	2017	BMC infectious diseases	Smear positive pulmonary tuberculosis and associated risk factors among tuberculosis suspects attending spiritual holy water sites in Northwest Ethiopia	exclude	No comparison group	NA
Desilva et. al	2018	Open Forum Infectious Diseases	An outbreak of multidrug-resistant tuberculosis, Minnesota 2016-2017	exclude	No comparison group	NA
Dey A et. al	2019	Trop Med Infect Dis	Active Case Finding for Tuberculosis through TOUCH Agents in Selected High TB Burden Wards of Kolkata, India: A Mixed Methods Study on Outcomes and Implementation Challenges	exclude	No comparison group	NA

Author	Year	Journal	Title	Decision	mainreason	Which review?
Dey et. al	2019	Trop Med Infect Dis	Active Case Finding for Tuberculosis through TOUCH Agents in Selected High TB Burden Wards of Kolkata, India: A Mixed Methods Study on Outcomes and Implementation Challenges	exclude	No comparison group	NA
Dhanaraj et. al	2015	PloS one	Prevalence and risk factors for adult pulmonary tuberculosis in a metropolitan city of South India	exclude	No comparison group	NA
Dhungana et. al	2013	Nepal Medical College journal : NMCJ	Surveillance of tuberculosis among HIV infected persons in three different regions of Nepal	exclude	No comparison group	NA
Di Naso et. al	2018	Biochimica Clinica	The molecular laboratory in the infectious disease emergency: The model "diagnosi in banchina"	exclude	No comparison group	NA
Diendere et. al	2011	Medecine tropicale : revue du Corps de sante colonial	[Prevalence and risk factors associated with infection by human immunodeficiency virus, hepatitis B virus, syphilis and bacillary pulmonary tuberculosis in prisons in Burkina Faso]	exclude	Fewer than 1000 people screened	NA
Dierberg et. al	2016	Emerging infectious diseases	Improved Detection of Tuberculosis and Multidrug-Resistant Tuberculosis among Tibetan Refugees, India	exclude	No comparison group	NA
Dion et. al	2018	Can Commun Dis Rep	Results of a population screening intervention for tuberculosis in a Nunavik village, Quebec, 2015-2016	exclude	No comparison group	NA
Dolla et. al	2017	Transactions of the Royal Society of Tropical Medicine and Hygiene	Tuberculosis among the homeless in Chennai city, South India	exclude	No comparison group	NA
Dolla et. al	2018	Indian Journal of Tuberculosis	Burden of pulmonary tuberculosis in modern prison: A cross sectional prevalence survey from south India	exclude	No comparison group	NA
Dolla et. al	2019	Indian J Tuberc	Burden of pulmonary tuberculosis in modern prison: A cross sectional prevalence survey from south India	exclude	No comparison group	NA
Dolla et. al	2019	Transactions of the Royal Society of Tropical Medicine and Hygiene	Age-specific prevalence of TB infection among household contacts of pulmonary TB: Is it time for TB preventive therapy?	exclude	No comparison group	NA
Dorjee et. al	2018	Clinical infectious diseases : an official publication of the Infectious Diseases Society of America	High Prevalence of Active and Latent Tuberculosis in Children and Adolescents in Tibetan Schools in India: The Zero TB Kids Initiative in Tibetan Refugee Children	exclude	No comparison group	NA
Dorjee et. al	2019	Clin Infect Dis	High Prevalence of Active and Latent Tuberculosis in Children and Adolescents in Tibetan Schools in India: The Zero TB Kids Initiative in Tibetan Refugee Children	exclude	No comparison group	NA

Author	Year	Journal	Title	Decision	mainreason	Which review?
Drain et. al	2016	BMC pulmonary medicine	Rapid urine lipoarabinomannan assay as a clinic-based screening test for active tuberculosis at HIV diagnosis	exclude	No comparison group	NA
Drain et. al	2017	Open forum infectious diseases	Clinic-Based Urinary Lipoarabinomannan as a Biomarker of Clinical Disease Severity and Mortality Among Antiretroviral Therapy-Naive Human Immunodeficiency Virus-Infected Adults in South Africa	exclude	No comparison group	NA
Drain et. al	2014	BMC infectious diseases	Diagnostic accuracy of a point-of-care urine test for tuberculosis screening among newly-diagnosed HIV-infected adults: a prospective, clinic-based study	exclude	No comparison group	NA
Dravid et. al	2019	BMC Infect Dis	Incidence of tuberculosis among HIV infected individuals on long term antiretroviral therapy in private healthcare sector in Pune, Western India	exclude	No comparison group	NA
Drevno et. al	2020	Gastroenterology	IMPROVING TUBERCULOSIS RE SCREENING IN INFLAMMATORY BOWEL DISEASE PATIENTS RECEIVING BIOLOGIC THERAPY: A SINGLE CENTER QUALITY IMPROVEMENT INITIATIVE	exclude	No comparison group	NA
Du et. al	2017	Journal of occupational health	Prevalence of tuberculosis among health care workers in tuberculosis specialized hospitals in China	exclude	No comparison group	NA
Duarte et. al	2018	European Respiratory Journal	Tuberculosis contact investigation-5-year experience of a Portuguese ambulatory center	exclude	No comparison group	NA
Duarte et. al	2018	Multiple Sclerosis Journal	Tuberculosis screening in patients with multiple sclerosis who are candidates for natalizumab and fingolimod in a Portuguese tertiary centre	exclude	No comparison group	NA
Dur and o et. al	2016	BMJ open	Prevalence and predictors of latent tuberculosis infection among Italian State Policemen engaged in assistance to migrants: a national cross-sectional study	exclude	No comparison group	NA
Durovni et. al	2013	The Lancet. Infectious diseases	Effect of improved tuberculosis screening and isoniazid preventive therapy on incidence of tuberculosis and death in patients with HIV in clinics in Rio de Janeiro, Brazil: a stepped wedge, cluster-randomised trial	exclude	Healthcare based screening	NA
Dutta et. al	2018	PLoS ONE	Impact of involvement of non-formal health providers on TB case notification among migrant slum-dwelling populations in Odisha, India	exclude	Healthcare based screening	NA

Author	Year	Journal	Title	Decision	mainreason	Which review?
Eang et. al	2012	BMC public health	Early detection of tuberculosis through community-based active case finding in Cambodia	exclude	Contact tracing	NA
Ebrahimi et. al	2013	Iranian journal of psychiatry	Frequency of latent and smear positive tuberculosis in chronic psychotic disorders	exclude	No comparison group	NA
Egere et. al	2017	The international journal of tuberculosis and lung disease : the official journal of the International Union against Tuberculosis and Lung Disease	Identifying children with tuberculosis among household contacts in The Gambia	exclude	No comparison group	NA
Ekeke et. al	2020	Adv Respir Med	Screening diabetes mellitus patients for tuberculosis in Southern Nigeria: A pilot study	exclude	No comparison group	NA
El Jihad et. al	2019	Turkish Journal of Gastroenterology	Screening for latent and patent tuberculosis in patients with cirrhosis	exclude	No comparison group	NA
Elden et. al	2011	BMC health services research	Integrating intensified case finding of tuberculosis into HIV care: an evaluation from rural Swaziland	exclude	No comparison group	NA
Endo et. al	2019	Epidemiology and Infection	A tuberculosis outbreak at an insecure, temporary housing facility, manga café, Tokyo, Japan, 2016–2017	exclude	No comparison group	NA
Enos et. al	2018	PloS one	Kenya tuberculosis prevalence survey 2016: Challenges and opportunities of ending TB in Kenya	exclude	No relevant data / not an ACF intervention	NA
Epstein et. al	2019	Int J Tuberc Lung Dis	QuantiFERON((R))-TB Gold In-Tube reliability for immigrants with parasitic infections in Boston, USA	exclude	No comparison group	NA
Epstein et. al	2019	International Journal of Tuberculosis and Lung Disease	QuantiFERON®-TB Gold In-Tube reliability for immigrants with parasitic infections in Boston, USA	exclude	No comparison group	NA
Erawati et. al	2020	J Multidiscip Healthc	The Prevalence and Demographic Risk Factors for Latent Tuberculosis Infection (LTBI) Among Healthcare Workers in Semarang, Indonesia	exclude	No comparison group	NA
Erme et. al	2017	Open Forum Infectious Diseases	A collaborative response by public health and Local Hospitals to a NICU tuberculosis exposure	exclude	No comparison group	NA
Estevan et. al	2013	Revista da Sociedade Brasileira de Medicina Tropical	Active and latent tuberculosis in prisoners in the Central-West Region of Brazil	exclude	No comparison group	NA
Faccini et. al	2013	Emerging infectious diseases	Tuberculosis outbreak in a primary school, Milan, Italy	exclude	No comparison group	NA
Fang et. al	2018	The Lancet. Public health	Travel-related infections in mainland China, 2014-16: an active surveillance study	exclude	No comparison group	NA

Author	Year	Journal	Title	Decision	mainreason	Which review?
Fang et. al	2013	Journal of epidemiology	Outbreak of pulmonary tuberculosis in a Chinese high school, 2009-2010	exclude	No comparison group	NA
Fang et. al	2013	Journal of epidemiology	Outbreak of pulmonary tuberculosis in a Chinese high school, 2009-2010	exclude	No comparison group	NA
Farazi et. al	2015	The Pan African medical journal	Silico-tuberculosis and associated risk factors in central province of Iran	exclude	No comparison group	NA
Farhoudi et. al	2019	Infectious Disorders - Drug Targets	Prevalence of tuberculosis in a prison in tehran by active case finding	exclude	No comparison group	NA
Farhoudi et. al	2018	Infectious disorders drug targets	Prevalence of Tuberculosis in a Prison in Tehran by Active Case Finding	exclude	NA	NA
Fatima et. al	2014	The international journal of tuberculosis and lung disease : the official journal of the International Union against Tuberculosis and Lung Disease	Success of active tuberculosis case detection among high-risk groups in urban slums in Pakistan	include	NA	CNR review
Fatima et. al	2016	PloS one	Extending 'Contact Tracing' into the Community within a 50-Metre Radius of an Index Tuberculosis Patient Using Xpert MTB/RIF in Urban, Pakistan: Did It Increase Case Detection?	include	NA	CNR review
Feasey et. al	2013	Journal of clinical microbiology	Evaluation of Xpert MTB/RIF for detection of tuberculosis from blood samples of HIV-infected adults confirms Mycobacterium tuberculosis bacteremia as an indicator of poor prognosis	exclude	No comparison group	NA
Feasey et. al	2013	Journal of clinical microbiology	Evaluation of Xpert MTB/RIF for detection of tuberculosis from blood samples of HIV-infected adults confirms Mycobacterium tuberculosis bacteremia as an indicator of poor prognosis	exclude	No comparison group	NA
Firanescu et. al	2019	Romanian Journal of Diabetes, Nutrition and Metabolic Diseases	Pulmonary Tuberculosis Screening in Patients with Diabetes Mellitus	exclude	No comparison group	NA
Floridia et. al	2017	Clinical infectious diseases : an official publication of the Infectious Diseases Society of America	Tuberculosis Case Finding With Combined Rapid Point-of-Care Assays (Xpert MTB/RIF and Determine TB LAM) in HIV-Positive Individuals Starting Antiretroviral Therapy in Mozambique	exclude	No comparison group	NA
Ford et. al	2019	Indian J Tuberc	Fifth year of a public-private partnership to improve the case detection of tuberculosis in India: A role model for future action?	include	NA	CNR review

Author	Year	Journal	Title	Decision	mainreason	Which review?
Fortunato et. al	2011	The international journal of tuberculosis and lung disease : the official journal of the International Union against Tuberculosis and Lung Disease	Screening and follow-up of children exposed to tuberculosis cases, Luanda, Angola	exclude	No comparison group	NA
Fox et. al	2017	The international journal of tuberculosis and lung disease : the official journal of the International Union against Tuberculosis and Lung Disease	Latent tuberculous infection in household contacts of multidrug-resistant and newly diagnosed tuberculosis	exclude	No comparison group	NA
Fox et. al	2019	The Lancet Infectious Diseases	Household contact investigation to improve tuberculosis control	exclude	Contact tracing	NA
Fox et. al	2012	PloS one	Contact investigation in households of patients with tuberculosis in Hanoi, Vietnam: a prospective cohort study	exclude	No comparison group	NA
Freeman et. al	2012	The New Zealand medical journal	Screening for Mycobacterium tuberculosis infection among healthcare workers in New Zealand: prospective comparison between the tuberculin skin test and the QuantiFERON-TB Gold In-Tube assay	exclude	No comparison group	NA
Fröberg et. al	2020	European Respiratory Journal	Screening and treatment of tuberculosis among pregnant women in Stockholm, Sweden, 2016–2017	exclude	No comparison group	NA
Fuge et. al	2016	BMC research notes	Prevalence of smear positive pulmonary tuberculosis and associated risk factors among prisoners in Hadiya Zone prison, Southern Ethiopia	exclude	No comparison group	NA
Gadallah et. al	2019	J Prev Med Hyg	Multicenter screening of diabetic patients for detecting new cases of tuberculosis: an approach to intensify the case detection rate of tuberculosis in developing countries with high prevalence of diabetes	exclude	No comparison group	NA
Ganmaa et. al	2019	BMC Infect Dis	Risk factors for active tuberculosis in 938 QuantiFERON-positive schoolchildren in Mongolia: a community-based cross-sectional study	exclude	No comparison group	NA
Ganmaa et. al	2019	Clin Infect Dis	Prevalence and Determinants of QuantiFERON-Diagnosed Tuberculosis Infection in 9810 Mongolian Schoolchildren	exclude	No comparison group	NA
Gao et. al	2017	The Lancet. Infectious diseases	Incidence of active tuberculosis in individuals with latent tuberculosis infection in rural China: follow-up results of a population-based, multicentre, prospective cohort study	exclude	No comparison group	NA

Author	Year	Journal	Title	Decision	mainreason	Which review?
Gao et. al	2017	The Lancet. Infectious diseases	Incidence of active tuberculosis in individuals with latent tuberculosis infection in rural China: follow-up results of a population-based, multicentre, prospective cohort study	exclude	No comparison group	NA
GarcÃa-GarcÃa Ma.D.L. et. al	2000	International Journal of Tuberculosis and Lung Disease	The role of core groups in transmitting Mycobacterium tuberculosis in a high prevalence community in Southern Mexico	exclude	No comparison group	NA
Garrido et. al	2012	Pediatric pulmonology	Usefulness of thoracic CT to diagnose tuberculosis disease in patients younger than 4 years of age	exclude	No comparison group	NA
Gashu et. al	2016	PloS one	The Yield of Community-Based "Retrospective" Tuberculosis Contact Investigation in a High Burden Setting in Ethiopia	exclude	No comparison group	NA
Gebrecherkos et. al	2019	Transactions of the Royal Society of Tropical Medicine and Hygiene	Prevalence, HIV co-infection and multi-drug resistance of smear positive pulmonary tuberculosis in prison settings of Northwest Ethiopia	exclude	No comparison group	NA
Gebrecherkos et. al	2016	BMC public health	Smear positive pulmonary tuberculosis and HIV co-infection in prison settings of North Gondar Zone, Northwest Ethiopia	exclude	No comparison group	NA
Gebrecherkos et. al	2016	BMC public health	Smear positive pulmonary tuberculosis and HIV co-infection in prison settings of North Gondar Zone, Northwest Ethiopia	exclude	No comparison group	NA
Gebreegziabiher et. al	2017	International journal of mycobacteriology	A survey on undiagnosed active pulmonary tuberculosis among pregnant mothers in mekelle and surrounding Districts in Tigray, Ethiopia	exclude	No comparison group	NA
Gedfew et. al	2020	Diabetes Metab Syndr Obes	Incidence and Predictors of Tuberculosis among Adult Diabetic Patients, Debre Markos Referral Hospital, Northwest Ethiopia, 2018: A Retrospective Cohort Study	exclude	No comparison group	NA
Gijón et. al	2016	Open Forum Infectious Diseases	Tuberculosis outbreak in a nursery school: A process developed during three years	exclude	No comparison group	NA
Gizachew Beza et. al	2017	International journal of bacteriology	Prevalence and Associated Factors of Tuberculosis in Prisons Settings of East Gojjam Zone, Northwest Ethiopia	exclude	No comparison group	NA
Gizachew Beza et. al	2017	International journal of bacteriology	Prevalence and Associated Factors of Tuberculosis in Prisons Settings of East Gojjam Zone, Northwest Ethiopia	exclude	No comparison group	NA
Glynn J.R. et. al	1998	Bulletin of the World Health Organization	Measurement and determinants of tuberculosis outcome in Karonga District, Malawi	exclude	No relevant data / not an ACF intervention	NA

Author	Year	Journal	Title	Decision	mainreason	Which review?
Godfrey-Faussett et. al	1995	Transactions of the royal society of tropical medicine and hygiene	Recruitment to a trial of tuberculosis preventive therapy from a voluntary HIV testing centre in Lusaka: relevance to implementation	exclude	No comparison group	NA
Goletti et. al	2020	Int J Infect Dis	Latent tuberculosis infection screening in persons newly-diagnosed with HIV infection in Italy: A multicentre study promoted by the Italian Society of Infectious and Tropical Diseases	exclude	No comparison group	NA
Golla et. al	2017	BMC infectious diseases	The impact of drug resistance on the risk of tuberculosis infection and disease in child household contacts: a cross sectional study	exclude	No comparison group	NA
GonzÃ¡lez-Ochoa E. et. al	2009	Tropical Medicine and International Health	Pulmonary tuberculosis case detection through fortuitous cough screening during home visits	exclude	NA	NA
Gopi P.G. et. al	2005	Indian J Tuberc	Failure to initiate treatment for tuberculosis patients diagnosed in a community survey and at health facilities under a DOTS programme in a district of South India	exclude	No comparison group	NA
Gopi P.G. et. al	2006	International Journal of Tuberculosis and Lung Disease	Yield of pulmonary tuberculosis cases by employing two screening methods in a community survey	exclude	No comparison group	NA
Gounder et. al	2011	Journal of acquired immune deficiency syndromes (1999)	Diagnostic accuracy of a urine lipoarabinomannan enzyme-linked immunosorbent assay for screening ambulatory HIV-infected persons for tuberculosis	exclude	No comparison group	NA
Gounder et. al	2011	Journal of acquired immune deficiency syndromes (1999)	Active tuberculosis case-finding among pregnant women presenting to antenatal clinics in Soweto, South Africa	exclude	No comparison group	NA
Govindasamy et. al	2013	PloS one	Linkage to HIV, TB and non-communicable disease care from a mobile testing unit in Cape Town, South Africa	exclude	No comparison group	NA
Gr and jean et. al	2011	The international journal of tuberculosis and lung disease : the official journal of the International Union against Tuberculosis and Lung Disease	Tuberculosis in household contacts of multidrug-resistant tuberculosis patients	exclude	No comparison group	NA
Graves et. al	2019	BMC Infect Dis	Tuberculosis infection risk, preventive therapy care cascade and incidence of tuberculosis disease in healthcare workers at Maputo Central Hospital	exclude	No comparison group	NA
Gray et. al	2020	J Public Health (Oxf)	Investigating the prevalence of latent Tuberculosis infection in a UK remand prison	exclude	No comparison group	NA

Author	Year	Journal	Title	Decision	mainreason	Which review?
Guerra et. al	2019	PloS one	Active and latent tuberculosis among inmates in La Esperanza prison in Guaduas, Colombia	exclude	No comparison group	NA
Guillen et. al	2019	European Respiratory Journal	TB status in a dynamic cohort of patients with inflammatory bowel disease receiving immunosuppression treatment, with up to 8 years of follow-up	exclude	No comparison group	NA
Gunasekera et. al	2020	Int J Tuberc Lung Dis	Smoking and HIV associated with subclinical tuberculosis: analysis of a population-based prevalence survey	exclude	No comparison group	NA
Guo et. al	2019	BMC Infect Dis	High incidence and low case detection rate among contacts of tuberculosis cases in Shanghai, China	exclude	No comparison group	NA
Guo et. al	2020	Epidemiology and Infection	An office building outbreak: The changing epidemiology of tuberculosis in Shenzhen, China	exclude	No comparison group	NA
Gupta-Wright et. al	2018	Lancet (London, England)	Rapid urine-based screening for tuberculosis in HIV-positive patients admitted to hospital in Africa (STAMP): a pragmatic, multicentre, parallel-group, double-blind, randomised controlled trial	exclude	No comparison group	NA
Gupta et. al	2011	Clinical infectious diseases : an official publication of the Infectious Diseases Society of America	Symptom screening among HIV-infected pregnant women is acceptable and has high negative predictive value for active tuberculosis	exclude	No comparison group	NA
Gupta et. al	2014	The international journal of tuberculosis and lung disease : the official journal of the International Union against Tuberculosis and Lung Disease	Active case finding for tuberculosis among people who inject drugs on methadone treatment in Dar es Salaam, Tanzania	exclude	No comparison group	NA
Gupta et. al	2019	N Engl J Med	Isoniazid Preventive Therapy in HIV-Infected Pregnant and Postpartum Women	exclude	Fewer than 1000 people screened	NA
Gupta et. al	2020	Clinical Infectious Diseases	Feasibility of identifying household contacts of rifampinand multidrug-resistant tuberculosis cases at high risk of progression to tuberculosis disease	exclude	No comparison group	NA
Gupta et. al	2016	Lung India : official organ of Indian Chest Society	Household symptomatic contact screening of newly diagnosed sputum smears positive tuberculosis patients - An effective case detection tool	exclude	No comparison group	NA
Gupta et. al	2015	Journal of human reproductive sciences	Should men with idiopathic obstructive azoospermia be screened for genitourinary tuberculosis?	exclude	No comparison group	NA

Author	Year	Journal	Title	Decision	mainreason	Which review?
Gupta et. al	2018	Thorax	Yield and cost of screening for active and latent tuberculosis among high-risk groups attending London emergency departments	exclude	No comparison group	NA
Gupta et. al	2013	JK Practitioner	Prevalence of tuberculosis in a rural population aged 15 years and above in R.S. pura block of district JAMMU	exclude	No comparison group	NA
Gurjav et. al	2019	Int. J. Tuberc. Lung Dis.	Vitamin D deficiency is associated with tuberculosis infection among household contacts in Ulaanbaatar, Mongolia	exclude	No comparison group	NA
Gurung et. al	2019	Infect Dis Poverty	The role of active case finding in reducing patient incurred catastrophic costs for tuberculosis in Nepal	exclude	Fewer than 1000 people screened	NA
Guwatudde D. et. al	2003	Bulletin of the World Health Organization	Burden of tuberculosis in Kampala, Uganda	exclude	No comparison group	NA
Gyawali et. al	2012	Nepal Medical College journal : NMCJ	Prevalence of tuberculosis in household contacts of sputum smears positive cases and associated demographic risk factors	exclude	No comparison group	NA
Gyawali et. al	2013	Nepal Medical College journal : NMCJ	Tobacco and alcohol: the relation to pulmonary tuberculosis in household contacts	exclude	Contact tracing	NA
Habib et. al	2019	European Respiratory Journal	Utilizing chest X-ray based active case finding approach for early tuberculosis case detection in Pakistan	exclude	No comparison group	NA
Habte et. al	2016	International journal of infectious diseases : IJID : official publication of the International Society for Infectious Diseases	The additional yield of GeneXpert MTB/RIF test in the diagnosis of pulmonary tuberculosis among household contacts of smear positive TB cases	exclude	No comparison group	NA
Hamdi et. al	2019	European Respiratory Journal	How to conduct a screening of tuberculosis in children with a household contact	exclude	No comparison group	NA
Hamusse et. al	2017	BMC infectious diseases	Prevalence and Incidence of Smear-Positive Pulmonary Tuberculosis in the Hetosa District of Arsi Zone, Oromia Regional State of Central Ethiopia	exclude	NA	NA
Han et. al	2019	BMC infectious diseases	Epidemiology survey of infectious diseases in North Korean travelers, 2015-2017	exclude	No comparison group	NA
Han et. al	2019	BMC infectious diseases	Epidemiology survey of infectious diseases in North Korean travelers, 2015-2017	exclude	No comparison group	NA
Han et. al	2019	PLoS One	Evaluation and treatment of latent tuberculosis infection among healthcare workers in Korea: A multicentre cohort analysis	exclude	No comparison group	NA

Author	Year	Journal	Title	Decision	mainreason	Which review?
Hanifa et. al	2012	The international journal of tuberculosis and lung disease : the official journal of the International Union against Tuberculosis and Lung Disease	Tuberculosis among adults starting antiretroviral therapy in South Africa: the need for routine case finding	exclude	No comparison group	NA
Hanifa et. al	2016	PloS one	Diagnostic Accuracy of Lateral Flow Urine LAM Assay for TB Screening of Adults with Advanced Immunosuppression Attending Routine HIV Care in South Africa	exclude	No comparison group	NA
Hanifa et. al	2015	PloS one	The diagnostic accuracy of urine lipoarabinomannan test for tuberculosis screening in a South African correctional facility	exclude	No comparison group	NA
Hanrahan et. al	2019	Paediatr Int Child Health	Diagnostic strategies for childhood tuberculosis in the context of primary care in a high burden setting: the value of alternative sampling methods	exclude	No comparison group	NA
Hanrahan et. al	2019	PLoS Med	Contact tracing versus facility-based screening for active TB case finding in rural South Africa: A pragmatic cluster-randomized trial (Kharitode TB)	exclude	Healthcare based screening	NA
Hansen et. al	2019	European Respiratory Journal	Benefits of a municipality tuberculosis screening program among socially vulnerable citizens	exclude	No comparison group	NA
Hargreaves et. al	2020	Travel Med Infect Dis	Delivering multi-disease screening to migrants for latent TB and blood-borne viruses in an emergency department setting: A feasibility study	exclude	No comparison group	NA
Harper I. et. al	1996	Tubercle and Lung Disease	Tuberculosis case finding in remote mountainous areas - Are microscopy camps of any value? Experience from Nepal	exclude	No comparison group	NA
Harries A.D. et. al	2004	International Journal of Tuberculosis and Lung Disease	Tuberculosis control in Malawian prisons: From research to policy and practice	exclude	NA	NA
Harstad et. al	2010	BMC public health	The role of entry screening in case finding of tuberculosis among asylum seekers in Norway	exclude	No comparison group	NA
Haynie et. al	2017	Open Forum Infectious Diseases	Interdisciplinary public health intervention in a multigenerational tuberculosis (TB) outbreak in harris county, Texas: A case study with implications for disease control process improvement and transmission cycle interruption	exclude	No comparison group	NA
Hazard et. al	2016	Infection control and hospital epidemiology	Hidden Reservoir: An Outbreak of Tuberculosis in Hospital Employees with No Patient Contact	exclude	No comparison group	NA

Author	Year	Journal	Title	Decision	mainreason	Which review?
Hazra et. al	2019	Journal of Pure and Applied Microbiology	Same day sputum microscopy for screening of pulmonary tuberculosis: Its accuracy and usefulness in comparison with conventional method	exclude	Healthcare based screening	NA
He et. al	2010	BMC Infectious Diseases	Infection control and the burden of tuberculosis infection and disease in health care workers in china: A cross-sectional study	exclude	No comparison group	NA
He et. al	2017	Infectious diseases of poverty	Use of low-dose computed tomography to assess pulmonary tuberculosis among healthcare workers in a tuberculosis hospital	exclude	No comparison group	NA
Henostroza et. al	2016	The international journal of tuberculosis and lung disease : the official journal of the International Union against Tuberculosis and Lung Disease	High prevalence of tuberculosis in newly enrolled HIV patients in Zambia: need for enhanced screening approach	exclude	No comparison group	NA
Henostroza et. al	2013	PloS one	The high burden of tuberculosis (TB) and human immunodeficiency virus (HIV) in a large Zambian prison: a public health alert	exclude	No comparison group	NA
Herchline et. al	2018	Open Forum Infectious Diseases	Treatment of latent tuberculosis infection in a refugee population	exclude	No comparison group	NA
Hermans et. al	2012	BMC public health	Implementation and effect of intensified case finding on diagnosis of tuberculosis in a large urban HIV clinic in Uganda: a retrospective cohort study	exclude	Healthcare based screening	NA
Hern and ez Sarmiento et. al	2013	Journal of immigrant and minority health	Tuberculosis among homeless population from Medellin, Colombia: associated mental disorders and socio-demographic characteristics	exclude	No comparison group	NA
Hern and ez Sarmiento et. al	2013	Journal of immigrant and minority health	Tuberculosis in indigenous communities of Antioquia, Colombia: epidemiology and beliefs	exclude	No comparison group	NA
Hern and ez-Leon et. al	2012	Salud publica de Mexico	[Active tuberculosis in a cohort of HIV-infected inmates in a prison in Mexico City: clinical and epidemiological characteristics]	exclude	Fewer than 1000 people screened	NA
Hernan Garcia et. al	2016	Archivos de bronconeumologia	Outbreak of isoniazid-resistant tuberculosis in an immigrant community in Spain	exclude	No comparison group	NA
Hiruy et. al	2018	International journal of infectious diseases : IJID : official publication of the International Society for Infectious Diseases	Comparison of the yield of tuberculosis among contacts of multidrug-resistant and drug-sensitive tuberculosis patients in Ethiopia using GeneXpert as a primary diagnostic test	exclude	Fewer than 1000 people screened	NA

Author	Year	Journal	Title	Decision	mainreason	Which review?
Hladun et. al	2014	Journal of travel medicine	Results from screening immigrants of low-income countries: data from a public primary health care	exclude	No comparison group	NA
Ho et. al	2016	The international journal of tuberculosis and lung disease : the official journal of the International Union against Tuberculosis and Lung Disease	The role of macroscopic sputum quality assessments to optimise sputum testing for tuberculosis	exclude	No comparison group	NA
Ho et. al	2016	The Lancet. Infectious diseases	Reassessment of the positive predictive value and specificity of Xpert MTB/RIF: a diagnostic accuracy study in the context of community-wide screening for tuberculosis	exclude	No relevant data / not an ACF intervention	NA
Hoa N.B. et. al	2010	Bulletin of the World Health Organization	National survey of tuberculosis prevalence in Viet Nam [EnquÃªte nationale sur la prÃ©valence de la tuberculose au Viet Nam]	exclude	No comparison group	NA
Hoang et. al	2019	BMC Public Health	Active contact tracing beyond the household in multidrug resistant tuberculosis in Vietnam: a cohort study	exclude	Fewer than 1000 people screened	NA
Hoffmann et. al	2013	PloS one	High prevalence of pulmonary tuberculosis but low sensitivity of symptom screening among HIV-infected pregnant women in South Africa	exclude	No comparison group	NA
Hom et. al	2012	PloS one	Drug-resistant tuberculosis among HIV-infected patients starting antiretroviral therapy in Durban, South Africa	exclude	No comparison group	NA
Honarvar et. al	2013	Journal of addiction medicine	Pulmonary and latent tuberculosis screening in opiate drug users: an essential and neglected approach for harm-reduction facilities	exclude	No comparison group	NA
Honarvar et. al	2014	The international journal of tuberculosis and lung disease : the official journal of the International Union against Tuberculosis and Lung Disease	Pulmonary tuberculosis in migratory nomadic populations: the missing link in Iran's National Tuberculosis Programme	exclude	No comparison group	NA
Hong Y.P. et. al	2000	International Journal of Tuberculosis and Lung Disease	Twenty-year trend of chronic excretors of tubercle bacilli based on the nationwide tuberculosis prevalence surveys in Korea, 1975-1995	exclude	No relevant data / not an ACF intervention	NA
Hong Y.P. et. al	1993	Tubercle and Lung Disease	The sixth Nationwide Tuberculosis Prevalence Survey in Korea, 1990	exclude	No relevant data / not an ACF intervention	NA

Author	Year	Journal	Title	Decision	mainreason	Which review?
Honjepari et. al	2019	Public Health Action	Implementation of screening and management of household contacts of tuberculosis cases in Daru, Papua New Guinea	exclude	No comparison group	NA
Hopkins et. al	2019	BMJ Open	Demographics and health profile on precursors of non-communicable diseases in adults testing for HIV in Soweto, South Africa: a cross-sectional study	exclude	No comparison group	NA
Horton et. al	2019	Open Forum Infectious Diseases	Public health at the United States/Mexico Border: evaluation of the county of San Diego health and human services agency's health screening assessment of asylum-seeking families at The San Diego rapid response network shelter	exclude	No comparison group	NA
Hoseinpoor et. al	2017	Int. J. Pediatr.	Evaluation of active case finding (ACF) of tuberculosis in slums population in North of Iran	exclude	No comparison group	NA
Hosten et. al	2018	Conflict and health	Tuberculosis contact-tracing among Syrian refugee populations: lessons from Jordan	exclude	No comparison group	NA
Hou et. al	2020	Am J Trop Med Hyg	Outbreak of Mycobacterium tuberculosis Beijing Strain in a High School in Yunnan, China	exclude	No comparison group	NA
Hsieh et. al	2019	Respir Care	The Risk of Latent Tuberculosis Infection in Respiratory Therapists in a Country with Intermediate Incidence	exclude	No comparison group	NA
Htet et. al	2018	BMC infectious diseases	Improving detection of tuberculosis among household contacts of index tuberculosis patients by an integrated approach in Myanmar: a cross-sectional study	exclude	No comparison group	NA
Huang et. al	2019	J Infect Dev Ctries	Enhanced directly-observed treatment short-course for tuberculosis control program in mountain areas of Taiwan	exclude	Contact tracing	NA
Huerga et. al	2019	Arch Dis Child	High prevalence of infection and low incidence of disease in child contacts of patients with drug-resistant tuberculosis: a prospective cohort study	exclude	No comparison group	NA
Humphreys et. al	2018	Thorax	Screening contacts of patients with extrapulmonary TB for latent TB infection	exclude	No comparison group	NA
Hussain et. al	2019	Indian Journal of Public Health Research and Development	Associated factors of latent tuberculosis among diabetics in Urban Health Clinics	exclude	No comparison group	NA
Hussain et. al	2020	PLoS One	Prevalence, risk factors and health seeking behaviour of pulmonary tuberculosis in four tribal dominated districts of Odisha: Comparison with studies in other regions of India	exclude	No comparison group	NA

Author	Year	Journal	Title	Decision	mainreason	Which review?
Hwang et. al	2019	Pediatric Infection and Vaccine	Childhood tuberculosis contact investigation and treatment of latent tuberculosis infection: A single center study, 2014–2017	exclude	No comparison group	NA
Igari et. al	2019	J Infect Chemother	Positivity rate of interferon-gamma release assays for estimating the prevalence of latent tuberculosis infection in renal transplant recipients in Japan	exclude	No comparison group	NA
Igari et. al	2019	Journal of Infection and Chemotherapy	Positivity rate of interferon-γ release assays for estimating the prevalence of latent tuberculosis infection in renal transplant recipients in Japan	exclude	No comparison group	NA
Imsanguan et. al	2020	Bull World Health Organ	Contact tracing for tuberculosis, Thailand	exclude	No comparison group	NA
Ines et. al	2018	European Respiratory Journal	Evaluation of Tuberculin skin test size and risk of tuberculosis in children household contact	exclude	No comparison group	NA
Iqbal et. al	2019	J. Pak. Med. Assoc.	Mycobacterium tuberculosis infection and resistance to rifampicin with GeneXpert®MTB/RIF: A single-center experience on bronchoalveolar lavage samples in renal failure patients	exclude	No comparison group	NA
Iqbal et. al	2019	Journal of Postgraduate Medical Institute	Causes and outcome of pleural effusion in children in a tertiary care hospital of Peshawar, Pakistan	exclude	No comparison group	NA
Iqbal et. al	2019	European Respiratory Journal	Validity of Pleural Fluid Protein in differentiating Tuberculous from Malignant Pleural Effusion	exclude	Healthcare based screening	NA
Iroezindu et. al	2016	Annals of medical and health sciences research	Factors Associated with Prevalent Tuberculosis Among Patients Receiving Highly Active Antiretroviral Therapy in a Nigerian Tertiary Hospital	exclude	No comparison group	NA
Isaakidis et. al	2014	PloS one	Alarming levels of drug-resistant tuberculosis in HIV-infected patients in metropolitan Mumbai, India	exclude	No comparison group	NA
Izumi et. al	2017	The international journal of tuberculosis and lung disease : the official journal of the International Union against Tuberculosis and Lung Disease	Evaluation of tuberculosis contact investigations in Japan	exclude	No comparison group	NA
Jacob et. al	2013	PloS one	Mycobacterium tuberculosis bacteremia in a cohort of hiv-infected patients hospitalized with severe sepsis in uganda-high frequency, low clinical suspicion [corrected] and derivation of a clinical prediction score	exclude	No comparison group	NA

Author	Year	Journal	Title	Decision	mainreason	Which review?
Jada et. al	2015	Research Journal of Pharmaceutical, Biological and Chemical Sciences	A comparison of clinical, laboratory and radiological imaging in assessing prevalence of pulmonary tuberculosis among adults in rural Kancheepuram, Tamil Nadu, India	exclude	No comparison group	NA
Jaganath et. al	2013	Clinical infectious diseases : an official publication of the Infectious Diseases Society of America	Contact investigation for active tuberculosis among child contacts in Uganda	exclude	No comparison group	NA
Jain et. al	2015	Asian Pacific Journal of Tropical Disease	Surveillance of tuberculosis co-infection among HIV infected patients and their CD4+ cell count profile	exclude	No comparison group	NA
Jamal et. al	2019	European Respiratory Journal	Active case finding for tuberculosis among prisoners in Karachi, Pakistan	exclude	No comparison group	NA
Jana et. al	2019	American Journal of Respiratory and Critical Care Medicine	Role of loop mediated isothermal amplification assay in detecting mycobacterium tuberculosis	exclude	No comparison group	NA
Janagond et. al	2017	International journal of mycobacteriology	Screening of health-care workers for latent tuberculosis infection in a Tertiary Care Hospital	exclude	No comparison group	NA
Janda et. al	2020	PLoS Med	Comprehensive infectious disease screening in a cohort of unaccompanied refugee minors in Germany from 2016 to 2017: A cross-sectional study	exclude	No comparison group	NA
Janssens et. al	2017	BMC infectious diseases	Screening for tuberculosis in an urban shelter for homeless in Switzerland: a prospective study	exclude	No comparison group	NA
Jasper et. al	2020	J Postgrad Med	Is routine pre-entry chest radiograph necessary in a high tuberculosis prevalence country?	exclude	No comparison group	NA
Javaid et. al	2016	Asian Pacific journal of tropical medicine	Screening outcomes of household contacts of multidrug-resistant tuberculosis patients in Peshawar, Pakistan	exclude	No comparison group	NA
Jayasooriya et. al	2019	Transactions of the Royal Society of Tropical Medicine and Hygiene	The hidden burden of chronic respiratory disease in patients attending tb clinics in the Gambia	exclude	No comparison group	NA
Jensen et. al	2016	The international journal of tuberculosis and lung disease : the official journal of the International Union against Tuberculosis and Lung Disease	Impact of contact investigation and tuberculosis screening among high-risk groups in Denmark	exclude	No comparison group	NA
Jensen et. al	2015	Thorax	Screening for TB by sputum culture in high-risk groups in Copenhagen, Denmark: a novel and promising approach	exclude	No comparison group	NA

Author	Year	Journal	Title	Decision	mainreason	Which review?
Jenum et. al	2018	BMJ open respiratory research	Incidence of tuberculosis and the influence of surveillance strategy on tuberculosis case-finding and all-cause mortality: a cluster randomised trial in Indian neonates vaccinated with BCG	exclude	ACF in children only	NA
Jereb J. et. al	2003	International Journal of Tuberculosis and Lung Disease	Tuberculosis contact investigations: Outcomes in selected areas of the United States, 1999	exclude	No comparison group	NA
Jerene et. al	2017	The international journal of tuberculosis and lung disease : the official journal of the International Union against Tuberculosis and Lung Disease	Tuberculosis along the continuum of HIV care in a cohort of adolescents living with HIV in Ethiopia	exclude	No comparison group	NA
Jerene et. al	2017	International health	The yield and feasibility of integrated screening for TB, diabetes and HIV in four public hospitals in Ethiopia	exclude	No comparison group	NA
Jerene et. al	2015	The international journal of tuberculosis and lung disease : the official journal of the International Union against Tuberculosis and Lung Disease	The yield of a tuberculosis household contact investigation in two regions of Ethiopia	exclude	No comparison group	NA
Ji et. al	2020	Int J Infect Dis	Screening for pulmonary tuberculosis in high-risk groups of diabetic patients	exclude	No comparison group	NA
Jia et. al	2014	BMC infectious diseases	Tuberculosis burden in China: a high prevalence of pulmonary tuberculosis in household contacts with and without symptoms	exclude	No comparison group	NA
Jimenez-Fuentes et. al	2014	The international journal of tuberculosis and lung disease : the official journal of the International Union against Tuberculosis and Lung Disease	Screening for active tuberculosis in high-risk groups	exclude	No comparison group	NA
John et. al	2015	The international journal of tuberculosis and lung disease : the official journal of the International Union against Tuberculosis and Lung Disease	Tuberculosis among nomads in Adamawa, Nigeria: outcomes from two years of active case finding	include	NA	CNR review
Jordan et. al	2019	Int J Tuberc Lung Dis	Prevalence and risk factors of tuberculosis disease in South African correctional facilities in 2015	exclude	No comparison group	NA
Josaphat et. al	2014	Revista portuguesa de pneumologia	Tuberculosis: which patients do not identify their contacts?	exclude	No comparison group	NA

Author	Year	Journal	Title	Decision	mainreason	Which review?
Joshi et. al	2015	Public health action	Impact of intensified case-finding strategies on childhood TB case registration in Nepal	exclude	ACF in children only	NA
Joshi et. al	2017	Bulletin of the World Health Organization	Peer-led active tuberculosis case-finding among people living with HIV: lessons from Nepal	exclude	No comparison group	NA
Kaiser et. al	2015	MMWR. Morbidity and mortality weekly report	Rapid large-scale deployment of tuberculosis testing in a high school - Riverside County, California, 2013-2014	exclude	No comparison group	NA
Kakar et. al	2018	Pak. J. Zool.	Study on accuracy and efficiency of molecular diagnostic techniques used for tuberculosis and analysis of associated risk factors for tuberculosis in jail inmates of Quetta, Pakistan	exclude	No comparison group	NA
Kakinda et. al	2016	BMC public health	A comparision of the yield of three tuberculosis screening modalities among people living with HIV: a retrospective quasi-experiemental study	exclude	Healthcare based screening	NA
Kall et. al	2012	BMC infectious diseases	Latent and subclinical tuberculosis in HIV infected patients: a cross-sectional study	exclude	No comparison group	NA
Kalonji et. al	2016	Tropical medicine and health	Prevalence of tuberculosis and associated risk factors in the Central Prison of Mbuji-Mayi, Democratic Republic of Congo	exclude	No comparison group	NA
Kambali et. al	2015	Journal of community health	A workplace tuberculosis case investigation in the presence of immigrant contacts from high prevalence countries	exclude	No comparison group	NA
Kamenska et. al	2019	J Infect Dev Ctries	Strategies for active detection of tuberculosis in Ukraine: Comparative effectiveness amongst key populations (2014-2018)	exclude	NA	NA
Kan et. al	2012	Public health action	Mobilising elementary and secondary school students for tuberculosis case finding in Anhui, China	include	NA	CNR review
Kancheya et. al	2014	The international journal of tuberculosis and lung disease : the official journal of the International Union against Tuberculosis and Lung Disease	Integrating active tuberculosis case finding in antenatal services in Zambia	exclude	No comparison group	NA
Kapadiya et. al	2018	Indian journal of community medicine : official publication of Indian Association of Preventive & Social Medicine	Assessment of Tuberculosis Prevalence in Newly Diagnosed Human Immunodeficiency Virus-Infected Adults Attending Care and Treatment Center in Gujarat, India	exclude	No comparison group	NA

Author	Year	Journal	Title	Decision	mainreason	Which review?
Kapata et. al	2016	PloS one	The Prevalence of Tuberculosis in Zambia: Results from the First National TB Prevalence Survey, 2013-2014	exclude	No comparison group	NA
Karamagi et. al	2018	BMC health services research	Improving TB case notification in northern Uganda: evidence of a quality improvement-guided active case finding intervention	include	NA	CNR review
Karki et. al	2017	Asia-Pacific journal of public health	Active Community-Based Case Finding for Tuberculosis With Limited Resources	exclude	No comparison group	NA
Kaswa et. al	2015	Tropical Medicine and International Health	Outbreak investigation of tuberculosis and multidrugresistant tuberculosis in the central prison of Mbuji-Mayi the diamond capital of the Democratic Republic of Congo	exclude	No comparison group	NA
Katelaris et. al	2020	J Infect Dis	Effectiveness of BCG Vaccination Against Mycobacterium tuberculosis Infection in Adults: A Cross-sectional Analysis of a UK-Based Cohort	exclude	No comparison group	NA
Kato-Maeda et. al	2019	Int J Tuberc Lung Dis	Magnitude of Mycobacterium tuberculosis transmission among household and non-household contacts of TB patients	exclude	No comparison group	NA
Keane V.P. et. al	1995	The Southeast Asian journal of tropical medicine and public health	Prevalence of tuberculosis in Vietnamese migrants: the experience of the Orderly Departure Program.	exclude	No comparison group	NA
Kebede et. al	2014	The international journal of tuberculosis and lung disease : the official journal of the International Union against Tuberculosis and Lung Disease	The first population-based national tuberculosis prevalence survey in Ethiopia, 2010-2011	exclude	No comparison group	NA
Kempker et. al	2012	Journal of immigrant and minority health	Quality improvement of tuberculosis screening in foreign-born patients	exclude	No comparison group	NA
Kempker et. al	2019	Open Forum Infect Dis	High Yield of Active Tuberculosis Case Finding Among HIV-Infected Patients Using Xpert MTB/RIF Testing	exclude	No comparison group	NA
Kerkhoff et. al	2020	N Engl J Med	Community-wide Screening for Tuberculosis	exclude	No relevant data / not an ACF intervention	NA
Kerkhoff et. al	2013	PLoS ONE	Blood Neutrophil Counts in HIV-Infected Patients with Pulmonary Tuberculosis: Association with Sputum Mycobacterial Load	exclude	No comparison group	NA
Khan A.J. et. al	2012	The Lancet Infectious Diseases	Engaging the private sector to increase tuberculosis case detection: An impact evaluation study	exclude	Healthcare based screening	NA

Author	Year	Journal	Title	Decision	mainreason	Which review?
Khan et. al	2016	Annals of global health	Active Case Finding of Tuberculosis: Randomized Evaluation of Simple and Infotainment Chest Camps	exclude	No relevant data / not an ACF intervention	NA
Khanal et. al	2016	Public health action	Yield of intensified tuberculosis case-finding activities using Xpert((R)) MTB/RIF among risk groups in Nepal	exclude	No comparison group	NA
Khaparde et. al	2015	Tuberculosis research and treatment	Evaluation of TB Case Finding through Systematic Contact Investigation, Chhattisgarh, India	exclude	No comparison group	NA
Khatana et. al	2019	Journal of Clinical Tuberculosis and Other Mycobacterial Diseases	Effectiveness, acceptance and feasibility of home-based intervention model for tuberculosis contact tracing in Kashmir	exclude	No comparison group	NA
Khatana et. al	2017	The Indian journal of tuberculosis	Factors affecting applicability of "home-based interventional model" for active case finding among household contacts of index cases of pulmonary tuberculosis in Kashmir	exclude	Fewer than 1000 people screened	NA
Khonelidze et. al	2019	Journal of Hepatology	Piloting of integrated HCV, TB and HIV screening model at primary care level in Georgia	exclude	No comparison group	NA
Kiertiburanakul et. al	2012	The Journal of hospital infection	Five-year prospective study of tuberculin skin testing among new healthcare personnel at a university hospital in Thailand	exclude	No comparison group	NA
Kigozi et. al	2019	BMC Public Health	Yield of systematic household contact investigation for tuberculosis in a high-burden metropolitan district of South Africa	exclude	No comparison group	NA
Kim et. al	2019	Tuberc Respir Dis (Seoul)	Experiences of Latent Tuberculosis Infection Treatment for the North Korean Refugees	exclude	No comparison group	NA
Kim et. al	2019	Tuberc Respir Dis (Seoul)	A Pilot Project of Systematic Tuberculosis Screening in the Elderly in a South Korean Province	exclude	No comparison group	NA
Kim et. al	2020	Tuberc Respir Dis (Seoul)	One Step toward a Low Tuberculosis-Burden Country: Screening for Tuberculosis Infection among the Immigrants and Refugees	exclude	No relevant data / not an ACF intervention	NA
Kim et. al	2020	Int J Tuberc Lung Dis	Symptom and digital chest X-ray TB screening in South African prisons: yield and cost-effectiveness	exclude	No comparison group	NA
Kim et. al	2012	Journal of Acquired Immune Deficiency Syndromes	Symptom screen for identification of highly infectious tuberculosis in people living with HIV in Southeast Asia	exclude	No comparison group	NA
Kim et. al	2020	Korean Journal of Internal Medicine	Quantiferon-tb gold plus versus quantifer-on-tb gold in-tube test for diagnosing tuberculosis infection	exclude	Fewer than 1000 people screened	NA

Author	Year	Journal	Title	Decision	mainreason	Which review?
Kim et. al	2017	Archives of environmental & occupational health	In-hospital contact investigation among health care workers after exposure to pulmonary tuberculosis in an intermediate tuberculosis prevalence area: A prospective study	exclude	No comparison group	NA
Kinikar et. al	2019	PLoS One	High risk for latent tuberculosis infection among medical residents and nursing students in India	exclude	No comparison group	NA
Kirkpatrick et. al	2006	Euro surveillance : bulletin Europeen sur les maladies transmissibles = European communicable disease bulletin	Investigation of a tuberculosis cluster at a job centre in Manchester, United Kingdom	exclude	No comparison group	NA
Kisa et. al	2016	Tuberkuloz ve toraks	Tuberculosis screening and efficacy of prophylaxis in contacts of patients with pulmonary tuberculosis	exclude	No comparison group	NA
Kliner et. al	2013	Public health action	Development and testing of models of tuberculosis contact tracing in rural southern Africa	exclude	No comparison group	NA
Koenig et. al	2015	Bulletin of the World Health Organization	Tuberculosis in the aftermath of the 2010 earthquake in Haiti	exclude	No comparison group	NA
Koenig et. al	2015	Bulletin of the World Health Organization	Tuberculosis in the aftermath of the 2010 earthquake in Haiti	exclude	No comparison group	NA
Koesoemadinata et. al	2018	The international journal of tuberculosis and lung disease : the official journal of the International Union against Tuberculosis and Lung Disease	Computer-assisted chest radiography reading for tuberculosis screening in people living with diabetes mellitus	exclude	No comparison group	NA
Koffi N. et. al	1997	International Journal of Tuberculosis and Lung Disease	Smear positive pulmonary tuberculosis in a prison setting: Experience in the penal camp of BouakÃ©, Ivory Coast	exclude	No comparison group	NA
Kolappan et. al	2013	The Indian journal of tuberculosis	Trends in the prevalence of pulmonary tuberculosis over a period of seven and half years in a rural community in south India with DOTS	include but duplicate	NA	Prevelance review
Kortas et. al	2017	Public health	Screening for infectious diseases among asylum seekers newly arrived in Germany in 2015: a systematic single-centre analysis	exclude	No comparison group	NA
Kosgei et. al	2011	Public health action	Symptom screen: diagnostic usefulness in detecting pulmonary tuberculosis in HIV-infected pregnant women in Kenya	exclude	No comparison group	NA
Kosgei et. al	2013	Public health action	Screening for tuberculosis in pregnancy: do we need more than a symptom screen? Experience from western Kenya	exclude	No comparison group	NA

Author	Year	Journal	Title	Decision	mainreason	Which review?
Krain et. al	2019	Journal of Investigative Dermatology	571 Comparing the performance of two interferon-gamma release assays in autoimmune skin disease patients: A prospective study	exclude	Healthcare based screening	NA
Kranzer et. al	2012	PLoS medicine	Feasibility, yield, and cost of active tuberculosis case finding linked to a mobile HIV service in Cape Town, South Africa: a cross-sectional study	exclude	No comparison group	NA
Kristensen et. al	2019	European Respiratory Journal	Long-term incidence of tuberculosis among migrants according to migrant status: A cohort study	exclude	No comparison group	NA
Krivinka R. et. al	1974	Bulletin of the World Health Organization	Epidemiological and clinical study of tuberculosis in the district of Kolin, Czechoslovakia. Second report (1965-1972)	exclude	NA	NA
Kuan et. al	2014	The international journal of tuberculosis and lung disease : the official journal of the International Union against Tuberculosis and Lung Disease	Tuberculosis among newly arrived foreign spouses before obtaining citizenship, Taiwan, 2006-2011	exclude	No comparison group	NA
Kubiak et. al	2018	International Journal of Tuberculosis and Lung Disease	Urinary LAM grade, culture positivity, and mortality among HIV-infected South African out-patients	exclude	No comparison group	NA
Kuehne et. al	2018	Eurosurveillance	Screening and prevention of infectious diseases in newly arrived migrants. Find and treat or find and lose? Tuberculosis treatment outcomes among screened newly arrived asylum seekers in Germany 2002 to 2014	exclude	No relevant data / not an ACF intervention	NA
Kuehne et. al	2018	Eurosurveillance	Screening and prevention of infectious diseases in newly arrived migrants. Find and treat or find and lose? Tuberculosis treatment outcomes among screened newly arrived asylum seekers in Germany 2002 to 2014	exclude	No comparison group	NA
Kufa et. al	2012	Journal of acquired immune deficiency syndromes (1999)	Undiagnosed tuberculosis among HIV clinic attendees: association with antiretroviral therapy and implications for intensified case finding, isoniazid preventive therapy, and infection control	exclude	No comparison group	NA
Kumar et. al	2020	PLoS Med	Health of Special Immigrant Visa holders from Iraq and Afghanistan after arrival into the United States using Domestic Medical Examination data, 2014-2016: A cross-sectional analysis	exclude	No comparison group	NA
Kumpatla et. al	2013	Public health action	Characteristics of patients with diabetes screened for tuberculosis in a tertiary care hospital in South India	exclude	No comparison group	NA

Author	Year	Journal	Title	Decision	mainreason	Which review?
Kundu et. al	2013	International Medical Journal	TB/HIV co-infection profile and impact of RNTCP and NACP liaison in the setting of free home based antiretroviral therapy in a rural tertiary teaching hospital in India	exclude	No comparison group	NA
Kunwipakorn et. al	2019	Journal of the Medical Association of Thailand	Community active case finding for pulmonary tuberculosis	exclude	Fewer than 1000 people screened	NA
Kurtz et. al	2019	Int J Tuberc Lung Dis	Effect of neonatal bacille Calmette-Guerin on the tuberculin skin test reaction in the first 2 years of life	exclude	No comparison group	NA
Kushner et. al	2019	Open Forum Infectious Diseases	Use of interferon-gamma release assays (IGRAs) reduced latent tuberculosis infection (LTBI) diagnosis in refugee and immigrant children	exclude	No comparison group	NA
Kyaw et. al	2019	Trop Med Infect Dis	Outcomes of Community-Based Systematic Screening of Household Contacts of Patients with Multidrug-Resistant Tuberculosis in Myanmar	exclude	No comparison group	NA
LaCourse et. al	2016	Journal of acquired immune deficiency syndromes (1999)	Tuberculosis Case Finding in HIV-Infected Pregnant Women in Kenya Reveals Poor Performance of Symptom Screening and Rapid Diagnostic Tests	exclude	No comparison group	NA
Laghari et. al	2019	BMC Public Health	Contact screening and risk factors for TB among the household contact of children with active TB: a way to find source case and new TB cases	exclude	No comparison group	NA
Lassausaie et. al	2015	Epidemiology and infection	Tuberculosis in Laos, who is at risk: the mahouts or their elephants?	exclude	No comparison group	NA
Law et. al	2015	Tropical medicine & international health : TM & IH	The first national tuberculosis prevalence survey of Lao PDR (2010-2011)	exclude	No comparison group	NA
Lawn et. al	2011	PLoS medicine	Screening for HIV-associated tuberculosis and rifampicin resistance before antiretroviral therapy using the Xpert MTB/RIF assay: a prospective study	exclude	No comparison group	NA
Lawn et. al	2017	BMC medicine	Diagnostic accuracy, incremental yield and prognostic value of Determine TB-LAM for routine diagnostic testing for tuberculosis in HIV-infected patients requiring acute hospital admission in South Africa: a prospective cohort	exclude	No comparison group	NA
Lawn et. al	2015	BMC medicine	Rapid microbiological screening for tuberculosis in HIV-positive patients on the first day of acute hospital admission by systematic testing of urine samples using Xpert MTB/RIF: a prospective cohort in South Africa	exclude	No comparison group	NA

Author	Year	Journal	Title	Decision	mainreason	Which review?
Lawn et. al	2012	The international journal of tuberculosis and lung disease : the official journal of the International Union against Tuberculosis and Lung Disease	Diagnostic yield of tuberculosis using sputum induction in HIV-positive patients before antiretroviral therapy	exclude	No comparison group	NA
Lawn et. al	2012	Clinical infectious diseases : an official publication of the Infectious Diseases Society of America	Characteristics and early outcomes of patients with Xpert MTB/RIF-negative pulmonary tuberculosis diagnosed during screening before antiretroviral therapy	exclude	No comparison group	NA
Lawn et. al	2012	AIDS (London, England)	Clinical significance of lipoarabinomannan detection in urine using a low-cost point-of-care diagnostic assay for HIV-associated tuberculosis	exclude	No comparison group	NA
Lawn et. al	2013	The international journal of tuberculosis and lung disease : the official journal of the International Union against Tuberculosis and Lung Disease	Diagnostic and prognostic value of serum C-reactive protein for screening for HIV-associated tuberculosis	exclude	No comparison group	NA
Lawn et. al	2013	BMC medicine	HIV-associated tuberculosis: relationship between disease severity and the sensitivity of new sputum-based and urine-based diagnostic assays	exclude	No comparison group	NA
Lawn et. al	2012	The Lancet. Infectious diseases	Diagnostic accuracy of a low-cost, urine antigen, point-of-care screening assay for HIV-associated pulmonary tuberculosis before antiretroviral therapy: a descriptive study	exclude	No comparison group	NA
Lebina et. al	2016	Tuberculosis research and treatment	The Use of Xpert MTB/Rif for Active Case Finding among TB Contacts in North West Province, South Africa	exclude	No comparison group	NA
Ledda et. al	2019	Future Microbiol	Tuberculosis screening among healthcare workers in Sicily, Italy	exclude	No comparison group	NA
Lee M.S.-N. et. al	2008	International Journal of Tuberculosis and Lung Disease	Early and late tuberculosis risks among close contacts in Hong Kong	exclude	No comparison group	NA
Lee et. al	2017	The Korean journal of internal medicine	Comparing tuberculin skin test and interferon gamma release assay (T-SPOT.TB) to diagnose latent tuberculosis infection in household contacts	exclude	No comparison group	NA
Lee et. al	2019	BMC Infect Dis	Impact of metformin use among tuberculosis close contacts with diabetes mellitus in a nationwide cohort study	exclude	No comparison group	NA

Author	Year	Journal	Title	Decision	mainreason	Which review?
Lee et. al	2019	Tuberc Res Treat	The Presence of Cough and Tuberculosis: Active Case Finding Outcomes in the Philippines	exclude	No comparison group	NA
Lee et. al	2019	Int J Tuberc Lung Dis	Long-term performance of the IGRA to predict and prevent active tuberculosis development in HIV-infected patients	exclude	No comparison group	NA
Lee et. al	2018	Tuberculosis and respiratory diseases	Pre-immigration Screening for Tuberculosis in South Korea: A Comparison of Smear- and Culture-Based Protocols	exclude	No comparison group	NA
Lee et. al	2019	Tuberc Respir Dis (Seoul)	Pre-immigration Screening for Tuberculosis in South Korea: A Comparison of Smear- and Culture-Based Protocols	exclude	NA	NA
Lee et. al	2019	Tuberc Respir Dis (Seoul)	Active Case Finding in the Elderly Tuberculosis in South Korea	exclude	No relevant data / not an ACF intervention	NA
Lee et. al	2015	PloS one	A Clinical Algorithm to Identify HIV Patients at High Risk for Incident Active Tuberculosis: A Prospective 5-Year Cohort Study	exclude	No comparison group	NA
Legesse et. al	2013	International journal of mycobacteriology	Community-based prevalence of undiagnosed mycobacterial diseases in the Afar Region, north-east Ethiopia	exclude	No comparison group	NA
Leung et. al	2013	The European respiratory journal	Transmission of multidrug-resistant and extensively drug-resistant tuberculosis in a metropolitan city	exclude	No comparison group	NA
Lewis et. al	2013	PloS one	Eligibility for isoniazid preventive therapy in South African gold mines	exclude	No comparison group	NA
Li et. al	2019	Int J Tuberc Lung Dis	Prevalence of pulmonary tuberculosis in Tibet Autonomous Region, China, 2014	exclude	No comparison group	NA
Li et. al	2018	European Respiratory Journal	Occupational tuberculosis screening for healthcare workers in a UK centre	exclude	No comparison group	NA
Liang et. al	2015	BMC public health	USA's expanded overseas tuberculosis screening program: a retrospective study in China	exclude	No comparison group	NA
Liang et. al	2019	J Interferon Cytokine Res	Comparison of Three Cellular Immunoassays to Detect Tuberculosis Infection in 876 Healthy Recruits	exclude	Fewer than 1000 people screened	NA
Liaquat et. al	2015	Pakistan journal of medical sciences	Concomitant presence of culture-proven active pulmonary tuberculosis in patients with chronic obstructive pulmonary disease - A hospital based study	exclude	No comparison group	NA

Author	Year	Journal	Title	Decision	mainreason	Which review?
Lim et. al	2016	Canadian respiratory journal	Is Universal Screening Necessary? Incidence of Tuberculosis among Tibetan Refugees Arriving in Calgary, Alberta	exclude	No comparison group	NA
Lin et. al	2010	BMC public health	"Cough officer screening" improves detection of pulmonary tuberculosis in hospital in-patients	exclude	No comparison group	NA
Lin et. al	2015	Tropical medicine & international health : TM & IH	Screening of patients with diabetes mellitus for tuberculosis in community health settings in China	exclude	No comparison group	NA
Lin et. al	2012	Tropical medicine & international health : TM & IH	Screening patients with diabetes mellitus for tuberculosis in China	exclude	Healthcare based screening	NA
Lin et. al	2015	BMC public health	Screening for pulmonary tuberculosis in type 2 diabetes elderly: a cross-sectional study in a community hospital	exclude	No comparison group	NA
Little et. al	2018	BMC infectious diseases	Yield of household contact tracing for tuberculosis in rural South Africa	exclude	No comparison group	NA
Liu et. al	2019	Int J Infect Dis	Assessment of active tuberculosis findings in the eastern area of China: A 3-year sequential screening study	include	NA	Prevelance review
Liu et. al	2015	Annals of internal medicine	Effect of a culture-based screening algorithm on tuberculosis incidence in immigrants and refugees bound for the United States: a population-based cross-sectional study	exclude	NA	NA
Lo et. al	2016	Global health action	Tuberculosis among transhumant pastoralist and settled communities of south-eastern Mauritania	exclude	No comparison group	NA
LoBue P.A. et. al	2004	Chest	Screening of immigrants and refugees for pulmonary tuberculosis in San Diego County, California	exclude	No comparison group	NA
Lohmann et. al	2012	The international journal of tuberculosis and lung disease : the official journal of the International Union against Tuberculosis and Lung Disease	Grading of a positive sputum smear and the risk of Mycobacterium tuberculosis transmission	exclude	No comparison group	NA
Lopes et. al	2019	J Bras Pneumol	Diagnosis and treatment of latent tuberculosis infection in patients undergoing treatment with immunobiologic agents: a four-year experience in an endemic area	exclude	No comparison group	NA
Lopez-Varela et. al	2015	The Pediatric infectious disease journal	Incidence of Tuberculosis Among Young Children in Rural Mozambique	exclude	ACF in children only	NA
Lopez-Varela et. al	2015	The Pediatric infectious disease journal	Incidence of Tuberculosis Among Young Children in Rural Mozambique	exclude	No comparison group	NA

Author	Year	Journal	Title	Decision	mainreason	Which review?
Lopez-Varela et. al	2019	J Acquir Immune Defic Syndr	High Yield of Home-Based TB Diagnosis Among Newly Diagnosed Patients With HIV	exclude	No comparison group	NA
Loredo et. al	2014	BMC pulmonary medicine	Yield of close contact tracing using two different programmatic approaches from tuberculosis index cases: a retrospective quasi-experimental study	exclude	No comparison group	NA
Lorent et. al	2015	The international journal of tuberculosis and lung disease : the official journal of the International Union against Tuberculosis and Lung Disease	Is frontloaded sputum microscopy an option in active tuberculosis case finding?	exclude	No comparison group	NA
Lorent et. al	2014	PloS one	Community-based active tuberculosis case finding in poor urban settlements of Phnom Penh, Cambodia: a feasible and effective strategy	include	NA	CNR review
Lowther et. al	2011	Public health reports (Washington, D.C. : 1974)	Outbreak of tuberculosis among Guatemalan immigrants in rural Minnesota, 2008	exclude	No comparison group	NA
Lu et. al	2019	Global Health	Tuberculosis among migrant workers in Taiwan	exclude	No comparison group	NA
Luabeya et. al	2015	The Pediatric infectious disease journal	Risk of Disease After Isoniazid Preventive Therapy for Mycobacterium tuberculosis Exposure in Young HIV-uninfected Children	exclude	No comparison group	NA
Lupisan et. al	2019	Int J Infect Dis	Etiology and epidemiology of community-acquired pneumonia in adults requiring hospital admission: A prospective study in rural Central Philippines	exclude	No comparison group	NA
Ly et. al	2019	J Epidemiol Glob Health	Preliminary Feasibility Study of Questionnaire-based Active Pulmonary Tuberculosis Screening in Marseille Sheltered Homeless People, Winter 2018	exclude	No comparison group	NA
M and alakas et. al	2017	PloS one	BUTIMBA: Intensifying the Hunt for Child TB in Swaziland through Household Contact Tracing	exclude	No comparison group	NA
Mabuto et. al	2015	BMC public health	Tuberculosis active case finding: uptake and diagnostic yield among minibus drivers in urban South Africa	exclude	No comparison group	NA
Machekera et. al	2019	Public Health Action	A comparison of the yield and relative cost of active tuberculosis case-finding algorithms in Zimbabwe	exclude	No comparison group	NA
Maggard et. al	2015	Bulletin of the World Health Organization	Screening for tuberculosis and testing for human immunodeficiency virus in Zambian prisons	include	NA	CNR review

Author	Year	Journal	Title	Decision	mainreason	Which review?
Mahomed et. al	2013	Tuberculosis (Edinburgh, Scotland)	Screening for TB in high school adolescents in a high burden setting in South Africa	exclude	No comparison group	NA
Mahomed et. al	2013	PloS one	TB incidence in an adolescent cohort in South Africa	exclude	No comparison group	NA
Majumder et. al	2016	The international journal of tuberculosis and lung disease : the official journal of the International Union against Tuberculosis and Lung Disease	Screening for active tuberculosis in a diabetes mellitus clinic in Soweto, South Africa	exclude	No comparison group	NA
Makay et. al	2019	Annals of the Rheumatic Diseases	Latent tuberculosis infection in children with pediatric rheumatologic diseases treated with canakinumab	exclude	Healthcare based screening	NA
Malacarne et. al	2019	J Bras Pneumol	Performance of diagnostic tests for pulmonary tuberculosis in indigenous populations in Brazil: the contribution of Rapid Molecular Testing	exclude	No comparison group	NA
Malik et. al	2018	The international journal of tuberculosis and lung disease : the official journal of the International Union against Tuberculosis and Lung Disease	Improving childhood tuberculosis detection and treatment through facility-based screening in rural Pakistan	exclude	No comparison group	NA
Mallick G et. al	2017	Public Health Action	Enhanced tuberculosis case finding through advocacy and sensitisation meetings in prisons of Central India	include	NA	CNR review
Mama et. al	2018	The open microbiology journal	Prevalence of Pulmonary Tuberculosis and Associated Factors Among HIV Positive Patients Attending Antiretroviral Therapy Clinic at Arba Minch General Hospital, Southern Ethiopia	exclude	No comparison group	NA
Mamani et. al	2016	The international journal of tuberculosis and lung disease : the official journal of the International Union against Tuberculosis and Lung Disease	Prevalence and incidence rates of latent tuberculous infection in a large prison in Iran	exclude	No comparison group	NA
Mamani et. al	2013	Iranian Red Crescent medical journal	Latent and active tuberculosis: evaluation of injecting drug users	exclude	No comparison group	NA
Manalo F. et. al	1990	American Review of Respiratory Disease	Community-based short-course treatment of pulmonary tuberculosis in a developing nation: Initial report of an eight-month, largely intermittent regimen in a population with a high prevalence of drug resistance	exclude	No comparison group	NA

Author	Year	Journal	Title	Decision	mainreason	Which review?
Mani et. al	2019	Lung India : official organ of Indian Chest Society	Is it feasible to carry out active case finding for tuberculosis in community-based settings?	exclude	No comparison group	NA
Mao et. al	2014	Bulletin of the World Health Organization	Cross-sectional studies of tuberculosis prevalence in Cambodia between 2002 and 2011	exclude	No comparison group	NA
Maokola et. al	2019	Front Public Health	Performance of and Factors Associated With Tuberculosis Screening and Diagnosis Among People Living With HIV: Analysis of 2012-2016 Routine HIV Data in Tanzania	exclude	No comparison group	NA
Maokola et. al	2020	Front. Public Health	Performance of and Factors Associated With Tuberculosis Screening and Diagnosis Among People Living With HIV: Analysis of 2012–2016 Routine HIV Data in Tanzania	exclude	No comparison group	NA
Margolis et. al	2013	The international journal of tuberculosis and lung disease : the official journal of the International Union against Tuberculosis and Lung Disease	Prevalence of tuberculosis symptoms and latent tuberculous infection among prisoners in northeastern Malaysia	exclude	No comparison group	NA
Marks et. al	2019	N Engl J Med	Community-wide Screening for Tuberculosis in a High-Prevalence Setting	include	NA	Prevelance review
Martinez et. al	2018	American Journal of Respiratory and Critical Care Medicine	A prospective validation of a clinical algorithm to detect tuberculosis in child contacts	exclude	No comparison group	NA
Martinez et. al	2018	The Lancet. Respiratory medicine	Effectiveness of WHO's pragmatic screening algorithm for child contacts of tuberculosis cases in resource-constrained settings: a prospective cohort study in Uganda	exclude	No comparison group	NA
Martinez et. al	2018	South African medical journal = Suid-Afrikaanse tydskrif vir geneeskunde	Tuberculin conversion and tuberculosis disease in infants and young children from the Drakenstein Child Health Study: A call to action	exclude	No comparison group	NA
Martinez et. al	2018	South African medical journal = Suid-Afrikaanse tydskrif vir geneeskunde	Tuberculin conversion and tuberculosis disease in infants and young children from the Drakenstein Child Health Study: A call to action	exclude	ACF in children only	NA
Masood-Us-Syed et. al	2012	Pakistan Paediatric Journal	Screening of childhood tuberculosis with Pakistan pediatric association scoring chart system	exclude	No comparison group	NA
Masood et. al	2016	Pakistan Journal of Medical and Health Sciences	Prevalence of Tuberculosis Among Patients having Diabetes Mellitis -A Cross-Sectional Study	exclude	Healthcare based screening	NA

Author	Year	Journal	Title	Decision	mainreason	Which review?
Mastrolia et. al	2018	Travel medicine and infectious disease	Utility of tuberculin skin test and IGRA for tuberculosis screening in internationally adopted children: Retrospective analysis from a single center in Florence, Italy	exclude	No comparison group	NA
Mastrolia et. al	2019	Travel Medicine and Infectious Disease	Utility of tuberculin skin test and IGRA for tuberculosis screening in internationally adopted children: Retrospective analysis from a single center in Florence, Italy	exclude	No comparison group	NA
Masur et. al	2017	The American journal of tropical medicine and hygiene	Active Tuberculosis Case Finding in Haiti	exclude	No comparison group	NA
Masur et. al	2017	The American journal of tropical medicine and hygiene	Active Tuberculosis Case Finding in Haiti	exclude	No comparison group	NA
Maung et. al	2017	Infectious Diseases of Poverty	The contribution of a non-governmental organisation's Community Based Tuberculosis Care Programme to case finding in Myanmar: trend over time	exclude	No comparison group	NA
Mave et. al	2017	BMC infectious diseases	Tuberculosis screening among persons with diabetes mellitus in Pune, India	exclude	No comparison group	NA
Mazahir et. al	2017	Egyptian Pediatric Association Gazette	Burden of tuberculosis among household children of adult multi drug resistant patients and their response to first line anti tubercular drugs	exclude	No comparison group	NA
Mbatchou Ngahane et. al	2019	American Journal of Respiratory and Critical Care Medicine	Prevalence of tuberculosis and its factors among patients on maintenance dialysis in Douala, Cameroon	exclude	No comparison group	NA
Mbu et. al	2018	PloS one	Tuberculosis in people newly diagnosed with HIV at a large HIV care and treatment center in Northwest Cameroon: Burden, comparative screening and diagnostic yields, and patient outcomes	exclude	No comparison group	NA
McAllister et. al	2017	Public health action	Feasibility of two active case finding approaches for detection of tuberculosis in Bandung City, Indonesia	exclude	No comparison group	NA
McAllister et. al	2020	Transactions of the Royal Society of Tropical Medicine and Hygiene	High tuberculosis incidence among people living with diabetes in Indonesia	exclude	No comparison group	NA
McBryde et. al	2012	The Medical journal of Australia	Risk of active tuberculosis in immigrants: effects of age, region of origin and time since arrival in a low-exposure setting	exclude	No comparison group	NA

Author	Year	Journal	Title	Decision	mainreason	Which review?
Mehari et. al	2019	Can J Infect Dis Med Microbiol	Prevalence and Factors Associated with Multidrug-Resistant Tuberculosis (MDR-TB) among Presumptive MDR-TB Patients in Tigray Region, Northern Ethiopia	exclude	No comparison group	NA
Meier et. al	2020	Pneumologie	[Latent Tuberculosis Infection (LTBI) among Medical Personnel after Foreign Assignments]	exclude	No comparison group	NA
Meier et. al	2016	International journal of hygiene and environmental health	Tuberculosis in newly arrived asylum seekers: A prospective 12 month surveillance study at Friedland, Germany	exclude	No comparison group	NA
Meijer J. et. al	1971	Bulletin of the International Union against Tuberculosis	Identification of sources of infection [Identification des sources d'infection.]	exclude	NA	NA
Mekonnen et. al	2018	The international journal of tuberculosis and lung disease : the official journal of the International Union against Tuberculosis and Lung Disease	Prevalence of pulmonary tuberculosis among students in three eastern Ethiopian universities	exclude	NA	NA
Melsew et. al	2019	BMC Infect Dis	The role of super-spreading events in Mycobacterium tuberculosis transmission: evidence from contact tracing	exclude	No comparison group	NA
Memish et. al	1995	The Canadian journal of infectious diseases = Journal canadien des maladies infectieuses	Evaluation and follow-up of infectious tuberculosis at the University of Ottawa	exclude	No comparison group	NA
Menzato et. al	2018	Blood	Successful simultaneous screening of sickle cell disease, hiv and tuberculosis in rural guinea bissau, west africa through rapid tests and a standardized clinical questionnaire: An outreach program due to a public-private partnership	exclude	No comparison group	NA
Merid et. al	2019	Int J Infect Dis	Population-based screening for pulmonary tuberculosis utilizing community health workers in Ethiopia	exclude	No comparison group	NA
Merid et. al	2018	The international journal of tuberculosis and lung disease : the official journal of the International Union against Tuberculosis and Lung Disease	High utility of active tuberculosis case finding in an Ethiopian prison	exclude	No comparison group	NA
Middelkoop K. et. al	2011	Journal of Acquired Immune Deficiency Syndromes	Antiretroviral therapy and TB notification rates in a high HIV prevalence South African community	exclude	Healthcare based screening	NA
Middelkoop K. et. al	2010	American Journal of Respiratory and Critical Care Medicine	Antiretroviral program associated with reduction in untreated prevalent tuberculosis in a South African township	exclude	No comparison group	NA

Author	Year	Journal	Title	Decision	mainreason	Which review?
Mijiti et. al	2016	The Lancet. Global health	Prevalence of pulmonary tuberculosis in western China in 2010-11: a population-based, cross-sectional survey	exclude	No relevant data / not an ACF intervention	NA
Miller A.C. et. al	2010	International Journal of Tuberculosis and Lung Disease	Controlled trial of active tuberculosis case finding in a Brazilian favela	include	NA	CNR review
Miller et. al	2010	International journal of tuberculosis and lung disease	Controlled trial of active tuberculosis case finding in a Brazilian favela	include but duplicate	NA	NA
Mirembe et. al	2019	Transactions of the Royal Society of Tropical Medicine and Hygiene	Strengthening tuberculosis diagnosis and notification through tb surge: Experience of Uganda protestant medical bureau	exclude	Healthcare based screening	NA
Miyahara et. al	2019	BMC Infect Dis	Predicting the risk of pulmonary tuberculosis based on the neutrophil-to-lymphocyte ratio at TB screening in HIV-infected individuals	exclude	No comparison group	NA
Modi et. al	2016	PloS one	Performance of Clinical Screening Algorithms for Tuberculosis Intensified Case Finding among People Living with HIV in Western Kenya	exclude	No comparison group	NA
Moges et. al	2012	BMC infectious diseases	Prevalence of smear positive pulmonary tuberculosis among prisoners in North Gondar Zone Prison, northwest Ethiopia	exclude	No comparison group	NA
Moh et. al	2017	The international journal of tuberculosis and lung disease : the official journal of the International Union against Tuberculosis and Lung Disease	Screening for active tuberculosis before isoniazid preventive therapy among HIV-infected West African adults	exclude	No comparison group	NA
Mohammed et. al	2020	J Clin Tuberc Other Mycobact Dis	Burden of tuberculosis and challenges related to screening and diagnosis in Ethiopia	exclude	No comparison group	NA
Mohareb et. al	2017	Open Forum Infectious Diseases	Latent tuberculosis infection in a cohort of refugee patients resettling in New England	exclude	Fewer than 1000 people screened	NA
Monegal et. al	2007	FMC formacion medica continuada en atencion primaria	Educational intervention to promote the screening of tuberculosis in primary care: randomized clinical trial with assigned clusters	exclude	Healthcare based screening	NA
Moosazadeh et. al	2015	Iranian journal of medical sciences	The prevalence of latent tuberculosis infection and smear positive pulmonary tuberculosis in people with household close contact with tuberculosis in north of iran	exclude	No comparison group	NA
Mor et. al	2012	Respiratory care	Chest radiography validity in screening pulmonary tuberculosis in immigrants from a high-burden country	exclude	No comparison group	NA

Author	Year	Journal	Title	Decision	mainreason	Which review?
Mor et. al	2015	The Israel Medical Association journal : IMAJ	The yield of tuberculosis screening of undocumented migrants from the Horn of Africa based on chest radiography	exclude	No comparison group	NA
Mor et. al	2015	The Israel Medical Association journal : IMAJ	The yield of tuberculosis screening of undocumented migrants from the Horn of Africa based on chest radiography	exclude	No comparison group	NA
Morano et. al	2013	Journal of community health	Latent tuberculosis infection: screening and treatment in an urban setting	exclude	No comparison group	NA
Morasert et. al	2018	The international journal of tuberculosis and lung disease : the official journal of the International Union against Tuberculosis and Lung Disease	Prevalence and risk factors associated with tuberculosis disease in Suratthani Central Prison, Thailand	exclude	No comparison group	NA
Morishita et. al	2016	PLoS One	Increased Case Notification through Active Case Finding of Tuberculosis among Household and Neighbourhood Contacts in Cambodia	exclude	No relevant data / not an ACF intervention	NA
Morishita et. al	2017	PloS one	Bringing state-of-the-art diagnostics to vulnerable populations: The use of a mobile screening unit in active case finding for tuberculosis in Palawan, the Philippines	exclude	No comparison group	NA
Moucaut et. al	2013	Journal of occupational medicine and toxicology (London, England)	The effect of introducing IGRA to screen French healthcare workers for tuberculosis and potential conclusions for the work organisation	exclude	No comparison group	NA
Moyo et. al	2015	Public health action	Evaluation of tuberculin skin testing in tuberculosis contacts in Victoria, Australia, 2005-2013	exclude	No comparison group	NA
Moyo et. al	2012	The international journal of tuberculosis and lung disease : the official journal of the International Union against Tuberculosis and Lung Disease	Tuberculosis case finding for vaccine trials in young children in high-incidence settings: a randomised trial	exclude	ACF in children only	NA
Mtwangambate et. al	2014	Diabetic medicine : a journal of the British Diabetic Association	'Cough-triggered' tuberculosis screening among adults with diabetes in Tanzania	exclude	No comparison group	NA
Mueller-Hermelink et. al	2018	Euro surveillance : bulletin Europeen sur les maladies transmissibles = European communicable disease bulletin	Universal screening for latent and active tuberculosis (TB) in asylum seeking children, Bochum and Hamburg, Germany, September 2015 to November 2016	exclude	No comparison group	NA

Author	Year	Journal	Title	Decision	mainreason	Which review?
Mulder et. al	2011	The international journal of tuberculosis and lung disease : the official journal of the International Union against Tuberculosis and Lung Disease	Coverage and yield of tuberculosis contact investigations in the Netherlands	exclude	Contact tracing	NA
Munoz et. al	2018	Clinical infectious diseases : an official publication of the Infectious Diseases Society of America	QuantiFERON-TB Gold In-Tube as a Confirmatory Test for Tuberculin Skin Test in Tuberculosis Contact Tracing: A Noninferiority Clinical Trial	exclude	No comparison group	NA
Mupfumi et. al	2014	Open forum infectious diseases	Impact of Xpert MTB/RIF on Antiretroviral Therapy-Associated Tuberculosis and Mortality: A Pragmatic Randomized Controlled Trial	exclude	No comparison group	NA
Murray et. al	2019	Emerg Infect Dis	Prevalence of Tuberculosis in Children After Natural Disasters, Bohol, Philippines	exclude	No comparison group	NA
Muyoyeta et. al	2017	BMC infectious diseases	Digital CXR with computer aided diagnosis versus symptom screen to define presumptive tuberculosis among household contacts and impact on tuberculosis diagnosis	exclude	No comparison group	NA
Mwansa-Kambafwile et. al	2013	PloS one	Tuberculosis case finding: evaluation of a paper slip method to trace contacts	exclude	No comparison group	NA
Myint et. al	2017	Infectious diseases of poverty	Active case-finding for tuberculosis by mobile teams in Myanmar: yield and treatment outcomes	exclude	No comparison group	NA
Myint et. al	2019	Public Health Action	Additional active tuberculosis cases detected and costs incurred by a second household contact investigation	exclude	No comparison group	NA
Naidoo et. al	2014	Journal of acquired immune deficiency syndromes (1999)	High rates of tuberculosis in patients accessing HAART in rural South Africa	exclude	No comparison group	NA
Naidoo et. al	2014	Journal of acquired immune deficiency syndromes (1999)	High rates of tuberculosis in patients accessing HAART in rural South Africa	exclude	Healthcare based screening	NA
Nair et. al	2016	PloS one	Household Contact Screening and Yield of Tuberculosis Cases-A Clinic Based Study in Chennai, South India	exclude	No comparison group	NA
Narang et. al	2015	Journal of epidemiology and global health	Prevalence of pulmonary tuberculosis in Wardha district of Maharashtra, Central India	exclude	No comparison group	NA
Nasehi et. al	2017	Epidemiology and health	Prevalence of latent tuberculosis infection among tuberculosis laboratory workers in Iran	exclude	No comparison group	NA

Author	Year	Journal	Title	Decision	mainreason	Which review?
Nathavitharana et. al	2017	International Journal of Tuberculosis and Lung Disease	FAST implementation in Bangladesh: High frequency of unsuspected tuberculosis justifies challenges of scale-up	exclude	No comparison group	NA
Navarro et. al	2016	Jornal brasileiro de pneumologia : publicacao oficial da Sociedade Brasileira de Pneumologia e Tisilogia	Prevalence of latent Mycobacterium tuberculosis infection in prisoners	exclude	No comparison group	NA
Nduba et. al	2015	International journal of infectious diseases : IJID : official publication of the International Society for Infectious Diseases	Prevalence of tuberculosis in adolescents, western Kenya: implications for control programs	exclude	No comparison group	NA
Nduba et. al	2018	The Pediatric infectious disease journal	Incidence of Active Tuberculosis and Cohort Retention Among Adolescents in Western Kenya	exclude	No comparison group	NA
Nduba et. al	2018	The Pediatric infectious disease journal	Incidence of Active Tuberculosis and Cohort Retention Among Adolescents in Western Kenya	exclude	No comparison group	NA
Ndwiga et. al	2013	BMC health services research	Feasibility and effect of integrating tuberculosis screening and detection in postnatal care services: an operations research study	exclude	Healthcare based screening	NA
Nguyen et. al	2016	The international journal of tuberculosis and lung disease : the official journal of the International Union against Tuberculosis and Lung Disease	Yield of chest radiograph in tuberculosis screening for HIV-infected persons at a district-level HIV clinic	exclude	Healthcare based screening	NA
Nguyen et. al	2011	The international journal of tuberculosis and lung disease : the official journal of the International Union against Tuberculosis and Lung Disease	Improving the diagnosis of pulmonary tuberculosis in HIV-infected individuals in Ho Chi Minh City, Viet Nam	exclude	No comparison group	NA
Nguyen et. al	2012	Tuberculosis research and treatment	Performance of Clinical Algorithms for Smear-Negative Tuberculosis in HIV-Infected Persons in Ho Chi Minh City, Vietnam	exclude	No comparison group	NA
Ngwira et. al	2018	Clinical infectious diseases : an official publication of the Infectious Diseases Society of America	Screening for tuberculosis with Xpert MTB/RIF versus fluorescent microscopy among adults newly diagnosed with HIV in rural Malawi: a cluster randomized trial (CHEPETSA)	exclude	No comparison group	NA
Ngwira et. al	2019	Clin Infect Dis	Screening for Tuberculosis With Xpert MTB/RIF Assay Versus Fluorescent Microscopy Among Adults Newly Diagnosed With Human Immunodeficiency Virus in Rural Malawi: A Cluster Randomized Trial (Chepetsa)	exclude	Healthcare based screening	NA

Author	Year	Journal	Title	Decision	mainreason	Which review?
Nienhaus et. al	2013	Journal of occupational medicine and toxicology (London, England)	Screening for tuberculosis and the use of a borderline zone for the interpretation of the interferon-gamma release assay (IGRA) in Portuguese healthcare workers	exclude	No comparison group	NA
Nienhaus et. al	2014	Journal of occupational medicine and toxicology (London, England)	Tuberculosis screening at the Sainte-Anne Hospital in Paris - results of first and second IGRA	exclude	No comparison group	NA
Njau et. al	2010	East African journal of public health	Tuberculosis in HIV-infected Tanzanian children below 14 years	exclude	No comparison group	NA
Noeske et. al	2011	The international journal of tuberculosis and lung disease : the official journal of the International Union against Tuberculosis and Lung Disease	Controlling tuberculosis in prisons against confinement conditions: a lost case? Experience from Cameroon	exclude	No comparison group	NA
Nogueira et. al	2018	Revista de saude publica	Tuberculosis and latent infection in employees of different prison unit types	exclude	No comparison group	NA
Ntinginya et. al	2012	The international journal of tuberculosis and lung disease : the official journal of the International Union against Tuberculosis and Lung Disease	Performance of the Xpert(R) MTB/RIF assay in an active case-finding strategy: a pilot study from Tanzania	exclude	No comparison group	NA
Nuzzo et. al	2015	American journal of public health	Postarrival Tuberculosis Screening of High-Risk Immigrants at a Local Health Department	exclude	No comparison group	NA
O'Grady et. al	2012	Clinical infectious diseases : an official publication of the Infectious Diseases Society of America	Evaluation of the Xpert MTB/RIF assay at a tertiary care referral hospital in a setting where tuberculosis and HIV infection are highly endemic	exclude	No comparison group	NA
Ogbudebe et. al	2015	International journal of mycobacteriology	Reaching the underserved: Active tuberculosis case finding in urban slums in southeastern Nigeria	exclude	No comparison group	NA
Okada K. et. al	2012	International Journal of Tuberculosis and Lung Disease	Epidemiological impact of mass tuberculosis screening: A 2-year follow-up after a national prevalence survey	exclude	No comparison group	NA
Okelloh et. al	2019	Public Health Action	Lessons learned from community-based tuberculosis case-finding in western Kenya	exclude	NA	NA
Oliveira-Cortez et. al	2019	Am J Trop Med Hyg	Low Prevalence of Latent Tuberculosis Infection among Contacts of Smear-Positive Adults in Brazil	exclude	No comparison group	NA

Author	Year	Journal	Title	Decision	mainreason	Which review?
Oliwa et. al	2019	PLoS One	Diagnostic practices and estimated burden of tuberculosis among children admitted to 13 government hospitals in Kenya: An analysis of two years' routine clinical data	exclude	No comparison group	NA
Oloyede et. al	2013	East African medical journal	PREVALENCE, CO-PREVALENCE AND RISK FACTORS OF PULMONARY PARAGONIMIASIS AND PULMONARY TUBERCULOSIS IN NIGERIAN CHILDREN IN THE NIGER DELTA AREA	exclude	No comparison group	NA
Ongen et. al	2013	Tuberkuloz ve toraks	Pulmonary tuberculosis incidence in Turkish prisons: importance of screening and case finding strategies	exclude	No comparison group	NA
Oni et. al	2011	Thorax	High prevalence of subclinical tuberculosis in HIV-1-infected persons without advanced immunodeficiency: implications for TB screening	exclude	No comparison group	NA
Ormerod L.P. et. al	1993	Respiratory Medicine	Results of tuberculosis contact tracing: Blackburn 1982-1990	exclude	No comparison group	NA
Ortiz-Rico et. al	2015	Salud publica de Mexico	Conformance contrast testing between rates of pulmonary tuberculosis in Ecuadorian border areas	exclude	No comparison group	NA
Oshi et. al	2016	International journal of mycobacteriology	Does intensified case finding increase tuberculosis case notification among children in resource-poor settings? A report from Nigeria	exclude	ACF in children only	NA
Oshi et. al	2017	International journal of mycobacteriology	An evaluation of innovative community-based approaches and systematic tuberculosis screening to improve tuberculosis case detection in Ebonyi State, Nigeria	exclude	No relevant data / not an ACF intervention	NA
Ottmani S. et. al	2009	Eastern Mediterranean Health Journal	TB contact investigations: 12 years of experience in the National TB Programme, Morocco 1993-2004	exclude	No comparison group	NA
Owiti et. al	2019	BMC public health	Screening and testing for tuberculosis among the HIV-infected: outcomes from a large HIV programme in western Kenya	exclude	No comparison group	NA
Owokuhaisa et. al	2014	Advances in research	Prevalence of Pulmonary Tuberculosis among Prison Inmates at Mbarara Central Prison, South Western Uganda	exclude	No comparison group	NA
Pace-Asciak et. al	2013	The international journal of tuberculosis and lung disease : the official journal of the International Union against Tuberculosis and Lung Disease	Tuberculosis among undocumented boat migrants to Malta: implications for a migrant tuberculosis policy	exclude	No comparison group	NA

Author	Year	Journal	Title	Decision	mainreason	Which review?
Pacifici et. al	2010	Giornale Italiano di Medicina Tropicale	Screening for tuberculosis among asylum seekers: Experience from an immigration centre in Central Italy and literature review	exclude	No comparison group	NA
Padmapriyadarsini et. al	2016	The National medical journal of India	Effectiveness of symptom screening and incidence of tuberculosis among adults and children living with HIV infection in India	exclude	No comparison group	NA
Paiao et. al	2016	BMC infectious diseases	Impact of mass-screening on tuberculosis incidence in a prospective cohort of Brazilian prisoners	exclude	NA	NA
Pan et. al	2019	International journal of infectious diseases : IJID : official publication of the International Society for Infectious Diseases	Adolescent tuberculosis associated with tuberculosis exposure in classrooms and dorm rooms in Guangxi, China	exclude	No comparison group	NA
Parija et. al	2014	The international journal of tuberculosis and lung disease : the official journal of the International Union against Tuberculosis and Lung Disease	Impact of awareness drives and community-based active tuberculosis case finding in Odisha, India	include	NA	CNR review
Park et. al	2020	Sci Rep	Risk of active tuberculosis development in contacts exposed to infectious tuberculosis in congregate settings in Korea	exclude	No comparison group	NA
Pelissari et. al	2018	The international journal of tuberculosis and lung disease : the official journal of the International Union against Tuberculosis and Lung Disease	Prevalence and screening of active tuberculosis in a prison in the South of Brazil	exclude	No comparison group	NA
Perez-Porcuna et. al	2012	The Pediatric infectious disease journal	Evaluation of new strategies for the diagnosis of tuberculosis among pediatric contacts of tuberculosis patients	exclude	No comparison group	NA
Perry et. al	2012	Archives de pediatrie : organe officiel de la Societe francaise de pediatrie	[Neonatal exposure to active pulmonary tuberculosis in a maternity ward: screening and clinical course of a cohort of exposed infants]	exclude	No comparison group	NA
Pevzner et. al	2010	American journal of public health	Tuberculosis transmission and use of methamphetamines in Snohomish County, WA, 1991-2006	exclude	No comparison group	NA
Phanuphak et. al	2012	Journal of acquired immune deficiency syndromes (1999)	Using tuberculin skin test as an entry point to screen for latent and active tuberculosis in Thai people living with HIV	exclude	No comparison group	NA

Author	Year	Journal	Title	Decision	mainreason	Which review?
Philipsen et. al	2019	Int J Tuberc Lung Dis	Automated chest X-ray reading for tuberculosis in the Philippines to improve case detection: a cohort study	exclude	No comparison group	NA
Phipps et. al	2019	Journal of Pediatric Infectious Diseases	Screening Young Children for Latent Tuberculosis in England: Lessons Learned from the Field	exclude	ACF in children only	NA
Phuanukoonnon S. et. al	2010	Int J Tuberc Lung Dis	Burden of tuberculosis and health seeking behaviours of people with prolonged cough in rural png. 41st world conference on lung health of the international union against tuberculosis and lung disease, berlin, germany, 11-15 november 2010	exclude	No comparison group	NA
Phyo et. al	2019	Trop Med Infect Dis	Contact Investigation of Multidrug-Resistant Tuberculosis Patients: A Mixed-Methods Study from Myanmar	exclude	No comparison group	NA
Phyo et. al	2019	Int J Tuberc Lung Dis	High prevalence and incidence of tuberculosis in people living with the HIV in Mandalay, Myanmar, 2011-2017	exclude	No comparison group	NA
Pontarelli et. al	2019	Travel medicine and infectious disease	Screening for active and latent tuberculosis among asylum seekers in Italy: A retrospective cohort analysis	exclude	No comparison group	NA
Popovici et. al	2018	Epidemiology and infection	Cross-border outbreak of extensively drug-resistant tuberculosis linked to a university in Romania	exclude	No comparison group	NA
Pothukuchi et. al	2011	PloS one	Tuberculosis contact screening and isoniazid preventive therapy in a South Indian district: operational issues for programmatic consideration	exclude	No comparison group	NA
Pourakbari et. al	2019	Infectious Disorders - Drug Targets	Evaluation of the QuantiFERON®-TB gold in-tube assay and tuberculin skin test for the diagnosis of latent tuberculosis infection in an iranian referral hospital	exclude	No comparison group	NA
Powell et. al	2012	Public health reports (Washington, D.C. : 1974)	Passenger contact investigation associated with a transport driver with pulmonary tuberculosis	exclude	No comparison group	NA
Prasad BM et. al	2016	Indian J Tuberc	Lessons learnt from active tuberculosis case finding in an urban slum setting of Agra city, India.	exclude	No comparison group	NA
Prasad et. al	2016	Public health action	Experience of active tuberculosis case finding in nearly 5 million households in India	exclude	No comparison group	NA
Prasad et. al	2017	International journal of infectious diseases : IJID : official publication of the International Society for Infectious Diseases	Status of Tuberculosis services in Indian Prisons	exclude	No comparison group	NA

Author	Year	Journal	Title	Decision	mainreason	Which review?
Puryear et. al	2013	The international journal of tuberculosis and lung disease : the official journal of the International Union against Tuberculosis and Lung Disease	Yield of contact tracing from pediatric tuberculosis index cases in Gaborone, Botswana	exclude	No comparison group	NA
Putra et. al	2019	J Epidemiol Glob Health	The Implementation of Early Detection in Tuberculosis Contact Investigation to Improve Case Finding	exclude	No comparison group	NA
Qadeer et. al	2017	Journal of Clinical Tuberculosis and Other Mycobacterial Diseases	Yield of facility-based verbal screening amongst household contacts of patients with multi-drug resistant tuberculosis in Pakistan	exclude	No comparison group	NA
Qader et. al	2019	Int J Infect Dis	Prevalence of tuberculosis among mentally ill patients in conflict-stricken Afghanistan: A cross-sectional study	exclude	No comparison group	NA
Rafiei et. al	2019	Nephrology (Carlton)	Mycobacterium tuberculosis: Active disease and latent infection in a renal transplant cohort	exclude	No comparison group	NA
Ramos et. al	2013	International journal of mycobacteriology	Screening for tuberculosis in family and household contacts in a rural area in Ethiopia over a 20-month period	exclude	No comparison group	NA
Rangaka et. al	2012	Clinical infectious diseases : an official publication of the Infectious Diseases Society of America	Effect of antiretroviral therapy on the diagnostic accuracy of symptom screening for intensified tuberculosis case finding in a South African HIV clinic	exclude	No comparison group	NA
Ranganath et. al	2018	Indian Journal of Public Health Research and Development	Child contact screening and chemoprophylaxis against tuberculosis in South Indian districts-situation analysis	exclude	No comparison group	NA
Rao et. al	2015	The Indian journal of tuberculosis	Yield of pulmonary tuberculosis cases by symptoms: Findings from a community survey in Madhya Pradesh, central India	exclude	No comparison group	NA
Rao et. al	2015	The Indian journal of medical research	Pulmonary tuberculosis - a health problem amongst Saharia tribe in Madhya Pradesh	exclude	No comparison group	NA
Rao et. al	2019	BMC Infect Dis	Declining tuberculosis prevalence in Saharia, a particularly vulnerable tribal community in Central India: evidences for action	include	NA	Prevelance review
Rao et. al	2010	International journal of infectious diseases	Pulmonary tuberculosis: a public health problem amongst the Saharia, a primitive tribe of Madhya Pradesh, Central India	exclude	No comparison group	NA

Author	Year	Journal	Title	Decision	mainreason	Which review?
Ratnawati and Burhan et. al	2019	J. Nat. Sci. Biol. Med.	Comparison of tuberculin skin test and interferon-gamma release assay in the diagnosis of latent tuberculosis infection among Indonesian health-care workers	exclude	Fewer than 1000 people screened	NA
Ratovoson et. al	2014	PloS one	Increase in the number of tuberculosis cases treated following tuberculin skin testing in first-year schoolchildren in Madagascar	exclude	No comparison group	NA
Rauf et. al	2018	Open Public Health J.	Low body mass index and trends of tuberculosis infection: A cohort study of orphan children in Azad Jammu and Kashmir Pakistan	exclude	No comparison group	NA
Reddy et. al	2015	Public health action	Intensified tuberculosis case finding amongst vulnerable communities in southern India	include	NA	CNR review
Reepalu et. al	2016	PloS one	Factors Associated with Early Mortality in HIV-Positive Men and Women Investigated for Tuberculosis at Ethiopian Health Centers	exclude	No comparison group	NA
Reichler et. al	2018	Journal of Infectious Diseases	Risk and timing of tuberculosis among close contacts of persons with infectious tuberculosis	exclude	No comparison group	NA
Reid et. al	2014	The international journal of tuberculosis and lung disease : the official journal of the International Union against Tuberculosis and Lung Disease	Screening for tuberculosis in a diabetes clinic in Gaborone, Botswana	exclude	No comparison group	NA
Rekha Devi et. al	2013	Pathogens and global health	Active detection of tuberculosis and paragonimiasis in the remote areas in North-Eastern India using cough as a simple indicator	exclude	No comparison group	NA
Rekha et. al	2013	The international journal of tuberculosis and lung disease : the official journal of the International Union against Tuberculosis and Lung Disease	Improving screening and chemoprophylaxis among child contacts in India's RNTCP: a pilot study	exclude	No comparison group	NA
Rendleman N.J. et. al	1999	American Journal of Preventive Medicine	Mandated tuberculosis screening in a community of homeless people	include	NA	CNR review
Reviono et. al	2019	J Korean Med Sci	Good Agreement between an Interferon Gamma Release Assay and Tuberculin Skin Tests in Testing for Latent Tuberculosis Infection among HIV-Infected Patients in Indonesia	exclude	No comparison group	NA
Ringshausen et. al	2013	PloS one	Frequent detection of latent tuberculosis infection among aged underground hard coal miners in the absence of recent tuberculosis exposure	exclude	No comparison group	NA

Author	Year	Journal	Title	Decision	mainreason	Which review?
Ritter et. al	2012	The international journal of tuberculosis and lung disease : the official journal of the International Union against Tuberculosis and Lung Disease	Prevalence of positive tuberculosis skin tests during 5 years of screening in a Swiss remand prison	exclude	No comparison group	NA
Rivera et. al	2017	The international journal of tuberculosis and lung disease : the official journal of the International Union against Tuberculosis and Lung Disease	Diagnostic yield of active case finding for tuberculosis and HIV at the household level in slums in Haiti	exclude	No comparison group	NA
Rivera et. al	2019	Int J Tuberc Lung Dis	Diagnostic yield of active case finding for tuberculosis at human immunodeficiency virus testing in Haiti	exclude	No comparison group	NA
Ross J.D. et. al	1977	Update	Pulmonary tuberculosis in the common hostel population	exclude	NA	NA
Roy et. al	2016	Journal of Acquired Immune Deficiency Syndromes	Use of symptom screening and sputum microscopy testing for active tuberculosis case detection among HIV-infected patients in real-world clinical practice in Uganda	exclude	No comparison group	NA
Roy et. al	2016	Journal of acquired immune deficiency syndromes (1999)	Implementation and Operational Research: Use of Symptom Screening and Sputum Microscopy Testing for Active Tuberculosis Case Detection Among HIV-Infected Patients in Real-World Clinical Practice in Uganda	exclude	No comparison group	NA
Rozhana et. al	2019	Kuwait Medical Journal	Clinico-microbiological study on 100 HIV seropositive patients from bangladesh	exclude	No comparison group	NA
Sabri et. al	2019	PLoS One	Prevalence and risk factors for latent tuberculosis infection among healthcare workers in Morocco	exclude	No comparison group	NA
Sah et. al	2016	Public health action	Dotting the Three I's for collaborative TB-HIV activities: evaluation of a pilot programme in Kathmandu, Nepal	exclude	No comparison group	NA
Said et. al	2019	Pediatr Infect Dis J	Immunologic-based Diagnosis of Latent Tuberculosis Among Children Younger Than 5 Years of Age Exposed and Unexposed to Tuberculosis in Tanzania	exclude	No comparison group	NA
Salas-Coronas et. al	2018	The American journal of tropical medicine and hygiene	Newly Arrived African Migrants to Spain: Epidemiology and Burden of Disease	exclude	No comparison group	NA
Salazar-Austin et. al	2019	Clinical infectious diseases : an official publication of the Infectious Diseases Society of America	Improving TPT Uptake: A Cluster-Randomized Trial of Symptom-Based Versus Tuberculin Skin Test-Based Screening of Household Tuberculosis Contacts Less than 5 Years of Age	exclude	ACF in children only	NA

Author	Year	Journal	Title	Decision	mainreason	Which review?
Salazar-Austin et. al	2020	Clin Infect Dis	Improving Tuberculosis Preventive Therapy Uptake: A Cluster-randomized Trial of Symptom-based Versus Tuberculin Skin Test-based Screening of Household Tuberculosis Contacts Less Than 5 Years of Age	exclude	ACF in children only	NA
Saleh et. al	2019	International Journal of Cancer Management	Association of lung cancer and tuberculosis: A cross sectional study from northwest of Iran	exclude	No comparison group	NA
Salinas et. al	2015	Medicina clinica	[Tuberculosis screening program for undocumented immigrant teenagers using the QuantiFERON((R))-TB Gold In-Tube test]	exclude	No comparison group	NA
Samayoa et. al	2020	Open Forum Infect Dis	The Diagnostic Laboratory Hub: A New Health Care System Reveals the Incidence and Mortality of Tuberculosis, Histoplasmosis, and Cryptococcosis of PWH in Guatemala	exclude	No comparison group	NA
Sanaie et. al	2016	PloS one	An Evaluation of Passive and Active Approaches to Improve Tuberculosis Notifications in Afghanistan	include	NA	CNR review
Sanchez et. al	2012	Epidemiology and infection	Extensive Mycobacterium tuberculosis circulation in a highly endemic prison and the need for urgent environmental interventions	include	NA	Prevelance review
Sanchez et. al	2013	BMC public health	X ray screening at entry and systematic screening for the control of tuberculosis in a highly endemic prison	include but duplicate	NA	NA
Sander et. al	2019	J Clin Tuberc Other Mycobact Dis	Systematic screening for tuberculosis among hospital outpatients in Cameroon: The role of screening and testing algorithms to improve case detection	exclude	No comparison group	NA
Sandhu et. al	2020	Int J STD AIDS	Implementation of routine interferon-gamma release assay testing in a South London HIV cohort	exclude	No comparison group	NA
Sane Schepisi et. al	2013	BMC public health	Tuberculosis case finding based on symptom screening among immigrants, refugees and asylum seekers in Rome	exclude	No comparison group	NA
Santha T. et. al	2003	International Journal of Tuberculosis and Lung Disease	Are community surveys to detect tuberculosis in high prevalence areas useful? Results of a comparative study from Tiruvallur District, South India	exclude	No comparison group	NA
Santos et. al	2020	Clin Infect Dis	Yield, Efficiency and Costs of Mass Screening Algorithms for Tuberculosis in Brazilian Prisons	exclude	No comparison group	NA
Sarin et. al	2018	The Indian journal of tuberculosis	Prevalence of pulmonary tuberculosis among adults in selected slums of Delhi city	exclude	No comparison group	NA

Author	Year	Journal	Title	Decision	mainreason	Which review?
Saunders et. al	2014	The international journal of tuberculosis and lung disease : the official journal of the International Union against Tuberculosis and Lung Disease	Predictors of contact tracing completion and outcomes in tuberculosis: a 21-year retrospective cohort study	exclude	No comparison group	NA
Saunders et. al	2019	Lancet Infect Dis	Active and passive case-finding in tuberculosis-affected households in Peru: a 10-year prospective cohort study	exclude	Contact tracing	NA
Sawka et. al	2019	Respirology	Five-year impact of a targeted screening program for latent tuberculosis infection in a high-risk population	exclude	No comparison group	NA
Sawry et. al	2018	The international journal of tuberculosis and lung disease : the official journal of the International Union against Tuberculosis and Lung Disease	Evaluation of the intensified tuberculosis case finding guidelines for children living with HIV	exclude	No comparison group	NA
Sayyahfar et. al	2020	Transpl Infect Dis	Comparison of tuberculin skin test and interferon gamma release assay in pediatric candidates of heart transplantation and a 2-year follow-up	exclude	Healthcare based screening	NA
Schechner et. al	2015	The Journal of hospital infection	Preventing tuberculosis transmission at a maternity hospital by targeted screening radiography of migrants	exclude	No comparison group	NA
Schepisi et. al	2016	Infectious disease reports	Active Tuberculosis Case Finding Interventions Among Immigrants, Refugees and Asylum Seekers in Italy	exclude	No comparison group	NA
Schneeberger Geisler et. al	2010	The international journal of tuberculosis and lung disease : the official journal of the International Union against Tuberculosis and Lung Disease	Screening for tuberculosis in asylum seekers: comparison of chest radiography with an interview-based system	exclude	NA	NA
Scotto et. al	2019	East Mediterr Health J	Screening for infectious diseases in newly arrived asymptomatic immigrants in southern Italy	exclude	No comparison group	NA
Sek and i J.N. et. al	2009	International Journal of Tuberculosis and Lung Disease	Active case finding of undetected tuberculosis among chronic coughers in a slum setting in kampala, uganda	exclude	No comparison group	NA
Sek and i et. al	2014	The international journal of tuberculosis and lung disease : the official journal of the International Union against Tuberculosis and Lung Disease	Yield of undetected tuberculosis and human immunodeficiency virus coinfection from active case finding in urban Uganda	exclude	No comparison group	NA

Author	Year	Journal	Title	Decision	mainreason	Which review?
Sellami et. al	2019	Egypt. Rheumatol.	Screening for latent tuberculosis infection prior to biologic therapy in patients with chronic immune-mediated inflammatory diseases (IMID): Interferon-gamma release assay (IGRA) versus tuberculin skin test (TST)	exclude	Healthcare based screening	NA
Sema Baltazar et. al	2020	PLoS One	HIV prevalence and TB in migrant miners communities of origin in Gaza Province, Mozambique: The need for increasing awareness and knowledge	exclude	No comparison group	NA
Semitala et. al	2019	J Acquir Immune Defic Syndr	Brief Report: Yield and Efficiency of Intensified Tuberculosis Case-Finding Algorithms in 2 High-Risk HIV Subgroups in Uganda	exclude	Healthcare based screening	NA
Semunigus et. al	2016	Annals of clinical microbiology and antimicrobials	Smear positive pulmonary tuberculosis and associated factors among homeless individuals in Dessie and Debre Birhan towns, Northeast Ethiopia	exclude	No comparison group	NA
Semunigus et. al	2016	Annals of clinical microbiology and antimicrobials	Smear positive pulmonary tuberculosis and associated factors among homeless individuals in Dessie and Debre Birhan towns, Northeast Ethiopia	exclude	No comparison group	NA
Sengai et. al	2019	Public Health Action	Mobile targeted screening for tuberculosis in Zimbabwe: diagnosis, linkage to care and treatment outcomes	exclude	No comparison group	NA
Seri et. al	2017	PloS one	Prevalence of pulmonary tuberculosis among prison inmates: A cross-sectional survey at the Correctional and Detention Facility of Abidjan, Cote d'Ivoire	exclude	No comparison group	NA
Sethuraman et. al	2018	Open Forum Infectious Diseases	Community prevalence of bacteriologically confirmed pulmonary tuberculosis: A 7-year retrospective study	exclude	No comparison group	NA
Seyedalinaghi et. al	2018	Archives of Clinical Infectious Diseases	Comparing tuberculosis incidence in a prison with the society, tehran, iran	exclude	No comparison group	NA
Shah et. al	2020	Lung India	Comparison of tuberculin skin test and QuantiFERON-TB Gold In-Tube test in Bacillus Calmette-Guerin-vaccinated children	exclude	ACF in children only	NA
Shah et. al	2013	The international journal of tuberculosis and lung disease : the official journal of the International Union against Tuberculosis and Lung Disease	Active contact investigation and treatment support: an integrated approach in rural and urban Sindh, Pakistan	exclude	No comparison group	NA
Shahryar et. al	2012	Life Sci. J.	Screening tuberculosis in the Sistan region of Iran: A population-based study	exclude	No comparison group	NA

Author	Year	Journal	Title	Decision	mainreason	Which review?
Shapiro et. al	2018	AIDS (London, England)	C-reactive protein as a screening test for HIV-associated pulmonary tuberculosis prior to antiretroviral therapy in South Africa	exclude	Healthcare based screening	NA
Shapiro et. al	2012	American journal of respiratory and critical care medicine	Community-based targeted case finding for tuberculosis and HIV in household contacts of patients with tuberculosis in South Africa	exclude	No comparison group	NA
Shargie E.B. et. al	2006	Bulletin of the World Health Organization	Tuberculosis case-finding through a village outreach programme in a rural setting in southern Ethiopia: Community randomized trial	include	NA	CNR review
Shargie E.B. et. al	2006	International Journal of Tuberculosis and Lung Disease	Prevalence of smear-positive pulmonary tuberculosis in a rural district of Ethiopia	exclude	No comparison group	NA
Sharma et. al	2018	Indian Journal of Tuberculosis	Tuberculosis (TB) intervention model targeting mobile population of truckers in Delhi, India	exclude	No comparison group	NA
Sharma et. al	2019	Indian J Tuberc	Tuberculosis (TB) intervention model targeting mobile population of truckers in Delhi, India	exclude	No comparison group	NA
Sharma et. al	2015	The Indian journal of medical research	Prevalence of tuberculosis in Faridabad district, Haryana State, India	exclude	No comparison group	NA
Shayo et. al	2014	Tropical medicine & international health : TM & IH	Symptom-based screening tool in ruling out active tuberculosis among HIV-infected patients eligible for isoniazid preventive therapy in Tanzania	exclude	No comparison group	NA
Shenoi et. al	2013	Public health action	'Cough officer' nurses in a general medical clinic successfully detect drug-susceptible and -resistant tuberculosis	exclude	No comparison group	NA
Shenoi et. al	2017	Open forum infectious diseases	Integrated Tuberculosis/Human Immunodeficiency Virus Community-Based Case Finding in Rural South Africa: Implications for Tuberculosis Control Efforts	exclude	No comparison group	NA
Shetty P.V.D. et. al	2008	International Journal of Tuberculosis and Lung Disease	Cross-referral between voluntary HIV counselling and testing centres and TB services, Maharashtra, India, 2003-2004	exclude	No comparison group	NA
Shewade et. al	2019	J Epidemiol Glob Health	Impact of Advocacy, Communication, Social Mobilization and Active Case Finding on TB Notification in Jharkhand, India	include	NA	CNR review
Shewade et. al	2018	Global health action	Active case finding among marginalised and vulnerable populations reduces catastrophic costs due to tuberculosis diagnosis	exclude	No relevant data / not an ACF intervention	NA

Author	Year	Journal	Title	Decision	mainreason	Which review?
Shinohara et. al	2020	Journal of Allergy and Clinical Immunology	Sex-difference In Associations Between Skin Responses To Purified Protein Derivative And Family Size During Infancy in Japan	exclude	No comparison group	NA
Shivakumar et. al	2016	Open Forum Infectious Diseases	Tuberculosis (TB) infection prevalence, incidence and risk factors among child and adult household contacts of adult TB cases in India	exclude	No comparison group	NA
Shrestha et. al	2019	Tuberc Res Treat	Pulmonary Tuberculosis among Male Inmates in the Largest Prison of Eastern Nepal	exclude	No comparison group	NA
Shriraam et. al	2020	Indian J Tuberc	Active case finding for Tuberculosis among migrant brick kiln workers in South India	exclude	No comparison group	NA
Shrivastava et. al	2013	Journal of research in health sciences	Tuberculosis: active case finding survey in an urban area of India, in 2012	exclude	No comparison group	NA
Silva et. al	2019	Rev Soc Bras Med Trop	Prevalence of coinfections in women living with human immunodeficiency virus in Northeast Brazil	exclude	No comparison group	NA
Silva et. al	2014	PloS one	Active case finding of tuberculosis (TB) in an emergency room in a region with high prevalence of TB in Brazil	exclude	No comparison group	NA
Singh et. al	2013	PloS one	Incidence and prevalence of tuberculosis among household contacts of pulmonary tuberculosis patients in a peri-urban population of South Delhi, India	exclude	No comparison group	NA
Singla et. al	2011	The international journal of tuberculosis and lung disease : the official journal of the International Union against Tuberculosis and Lung Disease	Tuberculosis among household contacts of multidrug-resistant tuberculosis patients in Delhi, India	exclude	No comparison group	NA
Sireesha et. al	2018	Infectious disorders drug targets	Surreptitious TB infections with recently identified DM people: A cross-sectional study	exclude	No comparison group	NA
Sireesha et. al	2019	Infectious Disorders - Drug Targets	Surreptitious TB infections with recently identified DM people: A cross-sectional study	exclude	Healthcare based screening	NA
Sismanidis C. et. al	2008	Clinical Trials	Restricted randomization of ZAMSTAR: A 2 Ã 2 factorial cluster randomized trial	include but duplicate	NA	NA
So-Ngern et. al	2019	Annals of the Rheumatic Diseases	Tuberculin skin test for detection of tuberculosis in systemic sclerosis	exclude	No comparison group	NA
Soares et. al	2011	American Journal of Respiratory and Critical Care Medicine	Community-based respiratory symptom evaluation in a Brazilian favela	exclude	No comparison group	NA

Author	Year	Journal	Title	Decision	mainreason	Which review?
Soe et. al	2017	Infectious Diseases of Poverty	International non-governmental organizations' provision of community-based tuberculosis care for hard-to-reach populations in Myanmar, 2013–2014	exclude	No comparison group	NA
Solari et. al	2019	Rev Peru Med Exp Salud Publica	[Respiratory symptoms in people attended in health facilities of the Ministry of Health in Lima, Peru]	exclude	No comparison group	NA
Sollai et. al	2017	Medicine	Infectious diseases prevalence, vaccination coverage, and diagnostic challenges in a population of internationally adopted children referred to a Tertiary Care Children's Hospital from 2009 to 2015	exclude	No comparison group	NA
Sookaromdee et. al	2019	Neurology Asia	Tuberculosis screening among the bed ridden patients after stroke: A note from a study in a rural province in Thailand	exclude	No comparison group	NA
Sotelo et. al	2019	Journal of the International AIDS Society	National study: Prevalence of HIV, hepatitis B and C, syphilis and tuberculosis in people deprived of liberty in federal prisons in Argentina	exclude	No comparison group	NA
Sousa et. al	2018	European Respiratory Journal	Cost-effectiveness of two latent tuberculosis infection screening strategies	exclude	No comparison group	NA
Spruijt et. al	2019	PLoS One	Implementation of latent tuberculosis infection screening and treatment among newly arriving immigrants in the Netherlands: A mixed methods pilot evaluation	exclude	No comparison group	NA
Sridhar et. al	2014	The Pediatric infectious disease journal	Increased risk of Mycobacterium tuberculosis infection in household child contacts exposed to passive tobacco smoke	exclude	No comparison group	NA
Ssemmondo et. al	2016	Journal of acquired immune deficiency syndromes (1999)	Implementation and Operational Research: Population-Based Active Tuberculosis Case Finding During Large-Scale Mobile HIV Testing Campaigns in Rural Uganda	exclude	No comparison group	NA
Story A. et. al	2008	Int J Tuberc Lung Dis	Targeted mobile digital radiography to reduce diagnostic delay for tuberculosis among hard to reach groups 39th world conference on lung health of the international union against tuberculosis and lung disease	exclude	No comparison group	NA
Subramani et. al	2016	The international journal of tuberculosis and lung disease : the official journal of the International Union against Tuberculosis and Lung Disease	Trend in the incidence of smear-positive tuberculosis in a district in South India after DOTS implementation	include	NA	Prevelance review

Author	Year	Journal	Title	Decision	mainreason	Which review?
Suh et. al	2018	Open Forum Infectious Diseases	2013-2015 nationwide tuberculosis contact investigation in childcare centers and schools in Korea	exclude	No comparison group	NA
Sulis et. al	2016	The international journal of tuberculosis and lung disease : the official journal of the International Union against Tuberculosis and Lung Disease	Active tuberculosis case finding among pregnant women: a pilot project in Burkina Faso	exclude	No comparison group	NA
Sun et. al	2019	Br J Dermatol	Screening for hepatitis B virus and tuberculosis infection in patients with moderate-to-severe psoriasis recruiting for biological therapy in China	exclude	No comparison group	NA
Swindells et. al	2018	The international journal of tuberculosis and lung disease : the official journal of the International Union against Tuberculosis and Lung Disease	Resource utilization for multidrug-resistant tuberculosis household contact investigations (A5300/I2003)	exclude	No comparison group	NA
Swindells et. al	2013	The international journal of tuberculosis and lung disease : the official journal of the International Union against Tuberculosis and Lung Disease	Screening for pulmonary tuberculosis in HIV-infected individuals: AIDS Clinical Trials Group Protocol A5253	exclude	No comparison group	NA
Szkwarko et. al	2016	Public health action	Implementing intensified tuberculosis case-finding among street-connected youth and young adults in Kenya	exclude	No comparison group	NA
Szkwarko et. al	2018	Public health action	Implementation of an active, clinic-based child tuberculosis contact management strategy in western Kenya	exclude	No comparison group	NA
Tabuchi et. al	2011	BMC infectious diseases	Tuberculosis infection among homeless persons and caregivers in a high-tuberculosis-prevalence area in Japan: a cross-sectional study	exclude	No comparison group	NA
Tadesse et. al	2011	PloS one	Two-thirds of smear-positive tuberculosis cases in the community were undiagnosed in Northwest Ethiopia: population based cross-sectional study	exclude	No comparison group	NA
Tadesse et. al	2013	The international journal of tuberculosis and lung disease : the official journal of the International Union against Tuberculosis and Lung Disease	Incidence of smear-positive tuberculosis in Dabat, northern Ethiopia	exclude	No comparison group	NA

Author	Year	Journal	Title	Decision	mainreason	Which review?
Tadesse et. al	2016	PloS one	Uptake of Isoniazid Preventive Therapy among Under-Five Children: TB Contact Investigation as an Entry Point	exclude	No comparison group	NA
Tafuri et. al	2011	American journal of infection control	Tuberculosis screening in migrant reception centers: results of a 2009 Italian survey	exclude	No comparison group	NA
Tagarro et. al	2011	Enfermedades infecciosas y microbiologia clinica	[Tuberculosis outbreak in a primary school: description and reflections on the value of gastric juice in the management of micro-epidemics]	exclude	No comparison group	NA
Tahseen et. al	2018	The international journal of tuberculosis and lung disease : the official journal of the International Union against Tuberculosis and Lung Disease	Systematic case finding for tuberculosis in HIV-infected people who inject drugs: experience from Pakistan	exclude	No comparison group	NA
Targowski et. al	2016	International Review of Allergology and Clinical Immunology in Family Medicine	Assessment of annual risk of tuberculosis infection (ARTI) based on Styblo's formula as well as on tuberculin (TST) and gamma-interferon (IGRA) tests results	exclude	No comparison group	NA
Tasaka et. al	2020	Epidemiol Infect	A tuberculosis outbreak in a psychiatric hospital: Kanagawa, Japan, 2012	exclude	No comparison group	NA
Tefera et. al	2019	BMC Health Serv Res	Evaluation of facility and community-based active household tuberculosis contact investigation in Ethiopia: a cross-sectional study	exclude	Contact tracing	NA
Telisinghe et. al	2014	PloS one	High tuberculosis prevalence in a South African prison: the need for routine tuberculosis screening	exclude	No comparison group	NA
Tewes et. al	2020	BMC Public Health	Tuberculosis screening during the 2015 European refugee crisis	exclude	No comparison group	NA
Thanh et. al	2014	BMC public health	A household survey on screening practices of household contacts of smear positive tuberculosis patients in Vietnam	exclude	No comparison group	NA
Thapa B et. al	2017	Indian J Tuberc	Adding sputum collection and transportation services for early identification TB cases in hard-to-reach difficult terrain—Will it help?	exclude	NA	NA
Thee et. al	2019	PLoS One	Screening and treatment for tuberculosis in a cohort of unaccompanied minor refugees in Berlin, Germany	exclude	ACF in children only	NA
Thibeault et. al	2012	Aviation, space, and environmental medicine	A case of active tuberculosis in a cabin crew: the results of contact tracing	exclude	No comparison group	NA

Author	Year	Journal	Title	Decision	mainreason	Which review?
Thind et. al	2012	The international journal of tuberculosis and lung disease : the official journal of the International Union against Tuberculosis and Lung Disease	An evaluation of 'Ribolola': a household tuberculosis contact tracing programme in North West Province, South Africa	exclude	No comparison group	NA
Thu et. al	2020	Tropical Medicine and Infectious Disease	An innovative public–private mix model for improving tuberculosis care in Vietnam: How well are we doing?	exclude	Healthcare based screening	NA
Tibbetts et. al	2020	Emerg Infect Dis	Public Health Response to Tuberculosis Outbreak among Persons Experiencing Homelessness, Minneapolis, Minnesota, USA, 2017-2018	exclude	No comparison group	NA
Titiyos et. al	2015	BMC research notes	The yield of screening symptomatic contacts of multidrug-resistant tuberculosis cases at a tertiary hospital in Addis Ababa, Ethiopia	exclude	No comparison group	NA
Tong et. al	2019	Am J Trop Med Hyg	Epidemic Situation of Tuberculosis in Prisons in the Central Region of China	exclude	No comparison group	NA
Torres Costa et. al	2011	Journal of occupational medicine and toxicology (London, England)	Screening for tuberculosis and prediction of disease in Portuguese healthcare workers	exclude	No comparison group	NA
Townes et. al	2016	Open Forum Infectious Diseases	Resource-intensive contact investigation resulting from an unrecognized pulmonary tuberculosis case at a rheumatology clinic	exclude	Healthcare based screening	NA
Trachanatzi et. al	2019	Acta Paediatr	Evaluating a 24-year tuberculosis screening in first-grade elementary schoolers in a low-burden area	exclude	ACF in children only	NA
Triasih et. al	2015	Tropical medicine & international health : TM & IH	Risk of infection and disease with Mycobacterium tuberculosis among children identified through prospective community-based contact screening in Indonesia	exclude	No comparison group	NA
Triasih et. al	2015	Clinical infectious diseases : an official publication of the Infectious Diseases Society of America	A prospective evaluation of the symptom-based screening approach to the management of children who are contacts of tuberculosis cases	exclude	No comparison group	NA
Trinidad et. al	2016	Journal of Clinical Tuberculosis and Other Mycobacterial Diseases	Tuberculosis screening at a diabetes clinic in the Republic of the Marshall Islands	exclude	No comparison group	NA
Tsegaye Sahle et. al	2019	PLoS One	Bacteriologically-confirmed pulmonary tuberculosis in an Ethiopian prison: Prevalence from screening of entrant and resident prisoners	include	NA	Prevelance review

Author	Year	Journal	Title	Decision	mainreason	Which review?
Tsuyuzaki et. al	2020	J Infect Chemother	Role of CD8 T-cell in immune response to tuberculosis-specific antigen in QuantiFERON-TB Gold Plus	exclude	No comparison group	NA
Tufa et. al	2017	Open Forum Infectious Diseases	Detecting TB cases among household contacts of patients with pulmonary tb through active contact tracing in The Arsi Zone, Ethiopia	exclude	No comparison group	NA
Tupasi T.E. et. al	1999	International Journal of Tuberculosis and Lung Disease	The 1997 nationwide tuberculosis prevalence survey in the Philippines	exclude	No comparison group	NA
Turinawe et. al	2016	PloS one	Operating Characteristics of a Tuberculosis Screening Tool for People Living with HIV in Out-Patient HIV Care and Treatment Services, Rwanda	exclude	No comparison group	NA
Uppada et. al	2016	BMC public health	Incidence of tuberculosis among school-going adolescents in South India	exclude	ACF in children only	NA
Usemann et. al	2019	Int J Tuberc Lung Dis	Cost-effectiveness of tuberculosis screening for migrant children in a low-incidence country	exclude	No comparison group	NA
Usemann et. al	2018	European Respiratory Journal	Cost-Effectiveness of Tuberculosis Screening for Migrant Children in Low-Incidence countries	exclude	No comparison group	NA
Usman et. al	2017	Pakistan Journal of Medical and Health Sciences	Tuberculin and sputum smear positivity among doctors and paramedics of a tertiary care hospital	exclude	No comparison group	NA
Usman et. al	2019	Sexually Transmitted Infections	Diagnostic accuracy of XPERT MTB/RIF in detecting pulmonary tuberculosis among people living with HIV in Western Nigeria	exclude	Healthcare based screening	NA
Ustero et. al	2017	PloS one	School and household tuberculosis contact investigations in Swaziland: Active TB case finding in a high HIV/TB burden setting	exclude	No comparison group	NA
Uwinkindi et. al	2014	Journal of acquired immune deficiency syndromes (1999)	Scaling up intensified tuberculosis case finding in HIV clinics in Rwanda	exclude	Healthcare based screening	NA
Valenca et. al	2015	The international journal of tuberculosis and lung disease : the official journal of the International Union against Tuberculosis and Lung Disease	Prevalence of tuberculosis in prisons: risk factors and molecular epidemiology	exclude	No comparison group	NA
van der Westhuizen et. al	2018	Southern African Journal of Infectious Diseases	Evaluation of a screening chest X-ray programme for the detection of pulmonary tuberculosis in asymptomatic military members???	exclude	No comparison group	NA
van Hest et. al	2016	The European respiratory journal	Active tuberculosis case-finding among drug users and homeless persons: after the outbreak	include but duplicate	NA	NA

Author	Year	Journal	Title	Decision	mainreason	Which review?
Van Hoving et. al	2020	J Acquir Immune Defic Syndr	Point-of-Care Ultrasound Predictors for the Diagnosis of Tuberculosis in HIV-Positive Patients Presenting to an Emergency Center	exclude	Healthcare based screening	NA
Van't Hoog A.H. et. al	2011	American Journal of Respiratory and Critical Care Medicine	High prevalence of pulmonary tuberculosis and inadequate case finding in rural Western Kenya	exclude	No relevant data / not an ACF intervention	NA
van't Hoog et. al	2011	American journal of respiratory and critical care medicine	High prevalence of pulmonary tuberculosis and inadequate case finding in rural western Kenya	exclude	No comparison group	NA
Vanino et. al	2017	Clinical infectious diseases : an official publication of the Infectious Diseases Society of America	Systematic Tuberculosis Screening in Asylum Seekers in Italy	exclude	No comparison group	NA
Varghese et. al	2019	Lung India	Prevalence of LTBI in patients with RA and AS	exclude	No comparison group	NA
Velasco-Arnaiz et. al	2018	Pediatric Infectious Disease Journal	Performance of Tuberculin Skin Tests and Interferon-γ Release Assays in Children Younger Than 5 Years	exclude	No comparison group	NA
Velasquez et. al	2012	Revista panamericana de salud publica = Pan American journal of public health	Tuberculosis testing among populations with high HIV risk in Tijuana, Baja California, Mexico	exclude	No comparison group	NA
Vella et. al	2011	The international journal of tuberculosis and lung disease : the official journal of the International Union against Tuberculosis and Lung Disease	Household contact investigation of multidrug-resistant and extensively drug-resistant tuberculosis in a high HIV prevalence setting	exclude	No comparison group	NA
Verdier et. al	2012	Infectious disease reports	Risk factors for tuberculosis in contact investigations in Rotterdam, the Netherlands	exclude	No comparison group	NA
Verma et. al	2012	Journal of Nepal Health Research Council	Prevalence of pulmonary tuberculosis among HIV infected persons in Pokhara, Nepal	exclude	No comparison group	NA
Verso et. al	2019	Int J Environ Res Public Health	Latent Tuberculosis Infection among Healthcare Students and Postgraduates in a Mediterranean Italian Area: What Correlation with Work Exposure?	exclude	No comparison group	NA
Verver S. et. al	2001	International Journal of Tuberculosis and Lung Disease	Screening for pulmonary tuberculosis among immigrants: Estimated effect on severity of disease and duration of infectiousness	exclude	No comparison group	NA
Verver et. al	2017	BMC public health	Feasibility of district wide screening of health care workers for tuberculosis in Zambia	exclude	No comparison group	NA

Author	Year	Journal	Title	Decision	mainreason	Which review?
Vieira et. al	2010	Revista brasileira de epidemiologia = Brazilian journal of epidemiology	Prevalence of patients with respiratory symptoms through active case finding and diagnosis of pulmonary tuberculosis among prisoners and related predictors in a jail in the city of Carapicuiba, Brazil	exclude	No comparison group	NA
Vijayageetha et. al	2019	Glob Health Action	Tuberculosis screening among pregnant women attending a tertiary care hospital in Puducherry, South India: is it worth the effort?	exclude	No comparison group	NA
Villa et. al	2019	Transactions of the Royal Society of Tropical Medicine and Hygiene	Tuberculosis risk among asylum-seekers and yield of interventions in Milan, Italy	exclude	No comparison group	NA
Villa et. al	2019	Eur Respir J	Tuberculosis among asylum seekers in Milan, Italy: epidemiological analysis and evaluation of interventions	exclude	No comparison group	NA
Villa et. al	2019	COPD: Journal of Chronic Obstructive Pulmonary Disease	[182] tuberculosis and latent tuberculosis infection screening among asylum seekers in Milan, Italy	exclude	No comparison group	NA
Visser et. al	2019	Western Pac Surveill Response J	Screening for latent tuberculosis infection by an Aboriginal Community Controlled Health Service, New South Wales, Australia, 2015	exclude	No comparison group	NA
von Streit et. al	2019	PLoS One	Prevalence of latent tuberculosis in homeless persons: A single-centre cross-sectional study, Germany	exclude	No comparison group	NA
Vyas et. al	2019	International Journal of Tuberculosis and Lung Disease	Community-based active case-finding to reach the most vulnerable: Tuberculosis in tribal areas of India	include	NA	CNR review
Waako et. al	2013	BMC infectious diseases	Burden of tuberculosis disease among adolescents in a rural cohort in Eastern Uganda	exclude	No comparison group	NA
Wali et. al	2019	BMC Public Health	Prevalence of tuberculosis, HIV/AIDS, and hepatitis; in a prison of Balochistan: a cross-sectional survey	exclude	No comparison group	NA
Wang P.D. et. al	2000	Journal of Infection	Tuberculosis transmission in the family	exclude	No comparison group	NA
Wang et. al	2014	Therapeutics and clinical risk management	Frequency of tuberculosis among diabetic patients in the People's Republic of China	exclude	Healthcare based screening	NA
Wang et. al	2012	The Journal of infection	Interferon-gamma release assay and Rifampicin therapy for household contacts of tuberculosis	exclude	No comparison group	NA
Wang et. al	2010	Journal of immigrant and minority health	Lessons learned from two school tuberculosis investigations	exclude	ACF in children only	NA

Author	Year	Journal	Title	Decision	mainreason	Which review?
Wang et. al	2017	PloS one	ESMPE: A combined strategy for school tuberculosis prevention and control proposed by Dalian, China	exclude	No comparison group	NA
Wardhani et. al	2019	Indian Journal of Public Health Research and Development	Relationship between diabetes mellitus and tuberculosis in Indonesia	exclude	No comparison group	NA
Warria et. al	2020	Trop Med Int Health	Tuberculosis disease and infection among household contacts of bacteriologically confirmed and non-confirmed tuberculosis patients	exclude	No comparison group	NA
Warrington et. al	2018	Canadian journal of public health = Revue canadienne de sante publique	Prevalence of latent tuberculosis infection in Syrian refugees to Canada	exclude	No comparison group	NA
Wei et. al	2014	BMC infectious diseases	Changes in pulmonary tuberculosis prevalence: evidence from the 2010 population survey in a populous province of China	exclude	No comparison group	NA
Wei et. al	2015	Transactions of the Royal Society of Tropical Medicine and Hygiene	An intervention of active TB case finding among smokers attending routine primary care facilities in China: an exploratory study	exclude	Healthcare based screening	NA
Weinrich et. al	2017	European radiology	Yield of chest X-ray tuberculosis screening of immigrants during the European refugee crisis of 2015: a single-centre experience	exclude	No comparison group	NA
Whalen et. al	2011	PloS one	Secondary attack rate of tuberculosis in urban households in Kampala, Uganda	exclude	No comparison group	NA
Wigg et. al	2019	Transpl Infect Dis	High rates of indeterminate interferon-gamma release assays for the diagnosis of latent tuberculosis infection in liver transplantation candidates	exclude	No comparison group	NA
Williams G. et. al	2007	Best practice for the care of patients with tuberculosis: A guide for low-income countries	[No title available]	exclude	No relevant data / not an ACF intervention	NA
Williams et. al	2020	Arch Dis Child	Screening for infection in unaccompanied asylum-seeking children and young people	exclude	No comparison group	NA
Williams et. al	2019	Archives of Disease in Childhood	Infection screening in unaccompanied asylum-seeking children	exclude	No comparison group	NA
Williams et. al	2016	Journal of public health (Oxford, England)	The need to implement effective new entrant tuberculosis screening in children: evidence from school 'outbreak'	exclude	No comparison group	NA
Winetsky et. al	2014	PloS one	Prevalence, risk factors and social context of active pulmonary tuberculosis among prison inmates in Tajikistan	exclude	No comparison group	NA

Author	Year	Journal	Title	Decision	mainreason	Which review?
Winetsky et. al	2012	PLoS medicine	Screening and rapid molecular diagnosis of tuberculosis in prisons in Russia and Eastern Europe: a cost-effectiveness analysis	exclude	No relevant data / not an ACF intervention	NA
Wingfield et. al	2018	Thorax	High prevalence of TB disease in contacts of adults with extrapulmonary TB	exclude	No comparison group	NA
Woldesemayat et. al	2015	PloS one	Follow-up of chronic coughers improves tuberculosis case finding: results from a community-based cohort study in southern Ethiopia	exclude	No comparison group	NA
Wong et. al	2020	ERJ Open Res	Prevalence of latent tuberculosis among refugee children in Malaysia	exclude	No comparison group	NA
Wood R. et. al	2007	American Journal of Respiratory and Critical Care Medicine	Undiagnosed tuberculosis in a community with high HIV prevalence: Implications for tuberculosis control	exclude	Fewer than 1000 people screened	NA
Wu et. al	2019	BMC Infect Dis	Diagnostic value of the interferon-gamma release assay for tuberculosis infection in patients with Behcet's disease	exclude	Healthcare based screening	NA
Xu et. al	2019	PLoS One	An outbreak of tuberculosis in a middle school in Henan, China: Epidemiology and risk factors	exclude	No comparison group	NA
Yadav et. al	2010	The Indian journal of tuberculosis	Prevalence of pulmonary tuberculosis amongst the Baigas--a primitive tribe of Madhya Pradesh, Central India	exclude	NA	NA
Yagi T. et. al	2006	Kekkaku	Clinical review of patients with pulmonary tuberculosis who were detected by the screening of homeless persons admitted in the shelter facilities	exclude	No comparison group	NA
Yasseen et. al	2019	The international journal of tuberculosis and lung disease : the official journal of the International Union against Tuberculosis and Lung Disease	Paediatric tuberculosis among the foreign-born: utility of the Canadian TB immigration medical surveillance programme	exclude	No comparison group	NA
Yassin et. al	2019	Current Women's Health Reviews	Prevalence of latent tuberculosis (LTB) among pregnant women in a high burden setting in Sudan using interferon gamma (IFN- γ) releasing assay (IGRA)	exclude	No comparison group	NA
Yassin et. al	2013	PloS one	Innovative community-based approaches doubled tuberculosis case notification and improve treatment outcome in Southern Ethiopia	include but duplicate	NA	NA

Author	Year	Journal	Title	Decision	mainreason	Which review?
Yeon et. al	2018	Scientific reports	Prevalence and risk factors of latent tuberculosis among Korean healthcare workers using whole-blood interferon-γ release assay	exclude	No comparison group	NA
Yezli et. al	2017	The American journal of tropical medicine and hygiene	Undiagnosed Active Pulmonary Tuberculosis among Pilgrims during the 2015 Hajj Mass Gathering: A Prospective Cross-sectional Study	exclude	No comparison group	NA
Yezli et. al	2017	The American journal of tropical medicine and hygiene	Undiagnosed Active Pulmonary Tuberculosis among Pilgrims during the 2015 Hajj Mass Gathering: A Prospective Cross-sectional Study	exclude	No comparison group	NA
Yimer S. et. al	2009	International Journal of Tuberculosis and Lung Disease	Evaluating an active case-fi nding strategy to identify smear-positive tuberculosis in rural Ethiopia	exclude	No comparison group	NA
Yoon et. al	2019	Am J Respir Crit Care Med	Yield and Efficiency of Novel Intensified Tuberculosis Case-Finding Algorithms for People Living with HIV	exclude	No comparison group	NA
Yoon et. al	2017	The Lancet. Infectious diseases	Point-of-care C-reactive protein-based tuberculosis screening for people living with HIV: a diagnostic accuracy study	exclude	No comparison group	NA
Yoon et. al	2019	Tuberculosis and Respiratory Diseases	The infectivity of pulmonary tuberculosis in Korean army units: Evidence from outbreak investigations	exclude	No comparison group	NA
You et. al	2019	Epidemiol Infect	A tuberculosis school outbreak in China, 2018: reaching an often overlooked adolescent population	exclude	No comparison group	NA
Young et. al	2016	MMWR. Morbidity and mortality weekly report	Tuberculosis Contact Investigations--United States, 2003-2012	exclude	No comparison group	NA
Yuen et. al	2019	Int J Tuberc Lung Dis	Optimizing the efficiency of tuberculosis active case-finding in health facilities and communities	exclude	NA	NA
Yuen et. al	2019	PLoS One	Tuberculosis household accompaniment to improve the contact management cascade: A prospective cohort study	exclude	Fewer than 1000 people screened	NA
Zaeh et. al	2013	Journal of Investigative Medicine	Improving tuberculosis screening and isoniazid preventative therapy in an HIV clinic in Addis Ababa, Ethiopia	exclude	Healthcare based screening	NA
Zaman et. al	2012	Epidemiology and infection	Prevalence of smear-positive tuberculosis in persons aged >/= 15 years in Bangladesh: results from a national survey, 2007-2009	exclude	No comparison group	NA
Zarnuzi and Wahyono et. al	2019	Indian Journal of Public Health Research and Development	Body mass index and lung tuberculosis in Indonesia: A cross-sectional in Indonesia	exclude	No comparison group	NA

Author	Year	Journal	Title	Decision	mainreason	Which review?
Zawedde-Muyanja et. al	2018	The international journal of tuberculosis and lung disease : the official journal of the International Union against Tuberculosis and Lung Disease	Decentralisation of child tuberculosis services increases case finding and uptake of preventive therapy in Uganda	exclude	Healthcare based screening	NA
Zenhausern et. al	2019	S Afr Med J	Tuberculosis transmission in a hospitalised neonate: Need for optimised tuberculosis screening of pregnant and postpartum women	exclude	Fewer than 1000 people screened	NA
Zhang et. al	2019	PLoS One	Findings from a pilot project to assess the feasibility of active tuberculosis case finding among seniors in rural Sichuan Province, China, 2017	exclude	No comparison group	NA
Zhang et. al	2019	Infectious diseases of poverty	Prevalence and risk factors of active pulmonary tuberculosis among elderly people in China: a population based cross-sectional study	exclude	No comparison group	NA
Zhang et. al	2020	Emerg Infect Dis	High Prevalence of and Risk Factors for Latent Tuberculosis Infection among Prisoners, Tianjin, China	exclude	No comparison group	NA
Zhang et. al	2019	Int J Tuberc Lung Dis	Serial T-SPOT.TB in household contacts of tuberculosis patients: a 6-year observational study in China	exclude	No comparison group	NA
Zhang et. al	2011	Tropical medicine & international health : TM & IH	Evaluation of active tuberculosis case finding through symptom screening and sputum microscopy of close contacts in Shandong, China	exclude	No comparison group	NA
Zhang et. al	2011	Tropical medicine & international health : TM & IH	Evaluation of active tuberculosis case finding through symptom screening and sputum microscopy of close contacts in Shandong, China	exclude	No comparison group	NA
Zhang et. al	2015	The international journal of tuberculosis and lung disease : the official journal of the International Union against Tuberculosis and Lung Disease	Integrating tuberculosis screening into annual health examinations for the rural elderly improves case detection	exclude	No comparison group	NA
Zhang et. al	2010	Chinese medical journal	Diagnosis of pulmonary tuberculosis among asymptomatic HIV+ patients in Guangxi, China	exclude	No comparison group	NA
Zhang et. al	2010	Chinese medical journal	Diagnosis of pulmonary tuberculosis among asymptomatic HIV+ patients in Guangxi, China	exclude	No comparison group	NA
Zhou et. al	2020	Radiology of Infectious Diseases	Outbreak of pulmonary tuberculosis in lodging high school, should X-ray be replaced by CT?	exclude	No comparison group	NA
Zimba et. al	2019	Pan Afr Med J	The effect of sputum quality and volume on the yield of bacteriologically-confirmed TB by Xpert MTB/RIF and smear	exclude	No comparison group	NA

Author	Year	Journal	Title	Decision	mainreason	Which review?
Zishiri et. al	2015	Open forum infectious diseases	Implementing a large-scale systematic tuberculosis screening program in correctional facilities in South Africa	exclude	No comparison group	NA
NA et. al	2011	The international journal of tuberculosis and lung disease : the official journal of the International Union against Tuberculosis and Lung Disease	Risk of tuberculosis among contacts of isoniazid-resistant and isoniazid-susceptible cases	exclude	No comparison group	NA
NA et. al	2013	Tropical medicine & international health : TM & IH	Screening of patients with diabetes mellitus for tuberculosis in India	exclude	No comparison group	NA
NA et. al	2019	Lancet	Causes of severe pneumonia requiring hospital admission in children without HIV infection from Africa and Asia: the PERCH multi-country case-control study	exclude	Healthcare based screening	NA
