# Supplementary material for: Do community-based active case-finding interventions have indirect impacts on wider TB case detection and determinants of subsequent TB testing behaviour? A systematic review
Source: PLOS Glob Public Health. 2021 Dec 8;1(12):e0000088. doi: 10.1371/journal.pgph.0000088 (PMC10021508; doi:10.1371/journal.pgph.0000088)
Supplement: S2 Table — (PDF) [file pgph.0000088.s005.pdf]

Appendix 4: List of TB ACF studies identified with suitable study design and included in search for additional KAP or qualitative manuscripts

Author	Year	Title	Decision	Main reason	Additional KAP/qualitative search
Adane et al (1)	2019	Tuberculosis case detection by trained inmate peer educators in a resource-limited prison setting in Ethiopia: a cluster-randomised trial	Proxy behavioural outcomes review		• 5 results<br>• 4 excluded on abstract<br>• Adane et al 2017 (2) excluded on full text as no data on impact of ACF
Aye et al (3)	2018	Evaluation of a tuberculosis active case finding project in peri-urban areas, Myanmar: 2014-2016	Routine CNR outcomes review		• 116 results<br>• All excluded on abstract
Ayles et al (4)	2013	Effect of household and community interventions on the burden of tuberculosis in southern Africa: the ZAMSTAR community-randomised trial	Exclude	No CNR data split routine : ACF	• 79 results<br>• 78 excluded on abstract<br>• Bond et al 2010 (5) excluded on full text as no data on impact of ACF
Cegielski et al (6)	2013	Eliminating tuberculosis one neighborhood at a time	Routine CNR outcomes review		• 12 results<br>• All excluded on abstract
Chatterjee et al (7)	2014	Incidence of Active Pulmonary Tuberculosis in Patients with Coincident Filarial and/or Intestinal Helminth Infections Followed Longitudinally in South India	Exclude	No CNR data split routine : ACF	• 80 results<br>• All excluded on abstract
Chen et al (8)	2019	Role of community-based active case finding in screening tuberculosis in Yunnan province of China	Exclude	No CNR data split routine : ACF	• 16 results<br>• All excluded on abstract
Churchyard et al (9)	2011	Twelve-monthly versus six-monthly radiological screening for active case-finding of tuberculosis: A randomised controlled trial	Exclude	TB screening not voluntary	• 127 results<br>• All excluded on abstract
Corbett et al (10)	2010	Comparison of two active case-finding strategies for community-based diagnosis of symptomatic smear-positive tuberculosis and control of infectious tuberculosis in Harare, Zimbabwe (DETECTB): A cluster-randomised trial	Routine CNR outcomes review		• 16 results<br>• All excluded on abstract
Dakito et al (11)<br><br>+ Yassin et al (12)	2017<br><br>2013	Health extension workers improve tuberculosis case finding and treatment outcome in Ethiopia: a large-scale implementation study.<br><br>Innovative community-based approaches doubled tuberculosis case notification and improve treatment outcome in Southern Ethiopia	Routine CNR outcomes review		• 32 results<br>• 31 excluded on abstract<br>• Tulloch et al 2015 (13) included
Dakito et al (14)	2009	Health extension workers improve tuberculosis case detection and treatment success in southern Ethiopia: A community randomized trial	Exclude	No CNR data split routine : ACF	• 49 results<br>• All excluded on abstract
Degner et al (15)	2016	Comparison of Digital Chest Radiography to Purified Protein Derivative for Screening of Tuberculosis in Newly Admitted Inmates	Exclude	No CNR data split routine : ACF	• 1 result<br>• Excluded on abstract
Delva et al (16)	2016	Active Tuberculosis Case Finding in Port-au-Prince, Haiti: Experiences, Results, and Implications for Tuberculosis Control Programs	Exclude	No CNR data split routine : ACF	• 1 result<br>• Excluded on abstract

de Vries et al (17)<br><br>+ van Hest et al (18)	2007<br><br>2016	Impact of mobile radiographic screening on tuberculosis among drug users and homeless persons<br><br>Active tuberculosis case-finding among drug users and homeless persons: after the outbreak	Exclude	No CNR data split routine : ACF	• 82 results<br>• All excluded on abstract
Fatima et al (19)	2014	Success of active tuberculosis case detection among high-risk groups in urban slums in Pakistan	Routine CNR outcomes review		• 34 results<br>• All excluded on abstract
Fatima et al (20)	2016	Extending 'Contact Tracing' into the Community within a 50-Metre Radius of an Index Tuberculosis Patient Using Xpert MTB/RIF in Urban, Pakistan: Did It Increase Case Detection?	Routine CNR outcomes review		• 29 results<br>• All excluded on abstract
Ford et al (21)	2019	Fifth year of a public-private partnership to improve the case detection of tuberculosis in India: A role model for future action?	Routine CNR outcomes review		• 3 results<br>• All excluded on abstract
John et al (22)	2015	Tuberculosis among nomads in Adamawa, Nigeria: outcomes from two years of active case finding	Exclude	Mobile population	• 5 results<br>• All excluded on abstract
Kan et al (23)	2012	Mobilising elementary and secondary school students for tuberculosis case finding in Anhui, China	Routine CNR outcomes review		• 30 results<br>• All excluded on abstract
Karamagi et al (24)	2018	Improving TB case notification in northern Uganda: evidence of a quality improvement-guided active case finding intervention	Exclude	No CNR data split routine : ACF	• 3 results<br>• All excluded on abstract
Kolappan et al (25)	2013	Trends in the prevalence of pulmonary tuberculosis over a period of seven and half years in a rural community in south India with DOTS	Exclude	No CNR data split routine : ACF	• 22 results<br>• All excluded on abstract
Liu et al (26)	2019	Assessment of active tuberculosis findings in the eastern area of China: A 3-year sequential screening study	Exclude	No CNR data split routine : ACF	• 81 results<br>• All excluded on abstract
Lorent et al (27)	2014	Community-based active tuberculosis case finding in poor urban settlements of Phnom Penh, Cambodia: a feasible and effective strategy	Routine CNR outcomes review		• 10 results<br>• 9 excluded on abstract<br>• 1 Lorent et al 2015 (28) included
Maggard et al (29)	2014	Screening for tuberculosis and testing for human immunodeficiency virus in Zambian prisons	Exclude	No CNR data split routine : ACF	• 16 results<br>• All excluded on abstract
Mallick et al (30)	2017	Enhanced tuberculosis case finding through advocacy and sensitisation meetings in prisons of Central India	Exclude	No CNR data split routine : ACF	• 88 results<br>• 86 excluded on abstract<br>• Thapa et al 2015 (31) & 2016 (32) excluded on further review due to misalignment of KAP surveys and ACF populations or timing
Marks et al (33)	2019	Community-wide Screening for Tuberculosis in a High-Prevalence Setting	Exclude	No CNR data split routine : ACF	• 21 results<br>• All excluded on abstract
Miller et al (34)	2009	Controlled trial of active tuberculosis case finding in a Brazilian favela	Routine CNR outcomes review		• 9 results<br>• All excluded on abstract
Parija et al (35)	2014	Impact of awareness drives and community-based active tuberculosis case finding in Odisha, India.	Routine CNR outcomes review		• 12 results<br>• All excluded on abstract

Rao et al (36)	2019	Declining tuberculosis prevalence in Saharia, a particularly vulnerable tribal community in Central India: evidences for action	Exclude	No CNR data split routine : ACF	• 36 results<br>• All excluded on abstract
Reddy et al (37)	2015	Intensified tuberculosis case finding amongst vulnerable communities in southern India	Exclude	No CNR data split routine : ACF	• 19 results<br>• 17 excluded on abstract<br>• Thapa et al 2015 (31) & 2016 (32) excluded on further review due to misalignment of KAP surveys and ACF populations or timing
Rendleman (38)	1999	Mandated tuberculosis screening in a community of homeless people	Exclude	No CNR data split routine : ACF	• 1 result<br>• Excluded on abstract
Sanaie et al (39)	2016	An Evaluation of Passive and Active Approaches to Improve Tuberculosis Notifications in Afghanistan	Exclude	No CNR data split routine : ACF	• 1 result<br>• Excluded on abstract
Sanchez et al (40)	2013	X ray screening at entry and systematic screening for the control of tuberculosis in a highly endemic prison	Exclude	No CNR data split routine : ACF	• 13 results<br>• All excluded on abstract
Shargie et al (41)	2006	Tuberculosis case-finding through a village outreach programme in a rural setting in southern Ethiopia: Community randomized trial	Exclude	No CNR data split routine : ACF	• 31 results<br>• All excluded on abstract
Shewade et al (42)	2019	Impact of Advocacy, Communication, Social Mobilization and Active Case Finding on TB Notification in Jharkhand, India	Exclude	No CNR data split routine : ACF	• 9 results<br>• 7 excluded on abstract<br>• Thapa et al 2015 (31) & 2016 (32) excluded on further review due to misalignment of KAP surveys and ACF populations or timing
Tsegaye Sahle et al (43)	2019	Bacteriologically-confirmed pulmonary tuberculosis in an Ethiopian prison: Prevalence from screening of entrant and resident prisoners	Exclude	No CNR data split routine : ACF	• 1 result<br>• Excluded on abstract
Vyas et al (44)	2018	Community-based active case-finding to reach the most vulnerable: tuberculosis in tribal areas of India	Routine CNR outcomes review		• 31 results<br>• All excluded on abstract

Reference list

1. Adane K, Spigt M, Winkens B, Dinant G-J. Tuberculosis case detection by trained inmate peer educators in a resource-limited prison setting in Ethiopia: a cluster-randomised trial. The Lancet Global Health. 2019;7(4):e482-e91.
2. Adane K, Spigt M, Johanna L, Noortje D, Abera SF, Dinant GJ. Tuberculosis knowledge, attitudes, and practices among northern Ethiopian prisoners: Implications for TB control efforts. PLoS One. 2017;12(3):e0174692.
3. Aye S, Majumdar SS, Oo MM, Tripathy JP, Satyanarayana S, Kyaw NTT, et al. Evaluation of a tuberculosis active case finding project in peri-urban areas, Myanmar: 2014-2016. Int J Infect Dis. 2018;70:93-100.
4. Ayles H, Muyoyeta M, Du Toit E, Schaap A, Floyd S, Simwinga M, et al. Effect of household and community interventions on the burden of tuberculosis in southern Africa: the ZAMSTAR community-randomised trial. Lancet. 2013;382(9899):1183-94.

5. Bond V, Chilikwela L, Simwinga M, Reade Z, Ayles H, Godfrey-Faussett P, et al. Children's role in enhanced case finding in Zambia. Int J Tuberc Lung Dis. 2010;14(10):1280-7.
6. Cegielski JP, Griffith DE, McGaha PK, Wolfgang M, Robinson CB, Clark PA, et al. Eliminating tuberculosis one neighborhood at a time. American journal of public health. 2013;103(7):1292-300.
7. Chatterjee S, Kolappan C, Subramani R, Gopi PG, Chandrasekaran V, Fay MP, et al. Incidence of active pulmonary tuberculosis in patients with coincident filarial and/or intestinal helminth infections followed longitudinally in South India. PLoS One. 2014;9(4):e94603.
8. Chen JO, Qiu YB, Rueda ZV, Hou JL, Lu KY, Chen LP, et al. Role of community-based active case finding in screening tuberculosis in Yunnan province of China. Infect Dis Poverty. 2019;8(1):92.
9. Churchyard GJ, Fielding K, Roux S, Corbett EL, Chaisson RE, De Cock KM, et al. Twelve-monthly versus six-monthly radiological screening for active case-finding of tuberculosis: a randomised controlled trial. Thorax. 2011;66(2):134-9.
10. Corbett EL, Bandason T, Duong T, Dauya E, Makamure B, Churchyard GJ, et al. Comparison of two active case-finding strategies for community-based diagnosis of symptomatic smear-positive tuberculosis and control of infectious tuberculosis in Harare, Zimbabwe (DETECTB): A cluster-randomised trial. The Lancet. 2010;376(9748):1244-53.
11. Datiko DG, Yassin MA, Theobald SJ, Blok L, Suvanand S, Creswell J, et al. Health extension workers improve tuberculosis case finding and treatment outcome in Ethiopia: a large-scale implementation study. BMJ Glob Health. 2017;2(4):e000390.
12. Yassin MA, Datiko DG, Tulloch O, Markos P, Aschalew M, Shargie EB, et al. Innovative community-based approaches doubled tuberculosis case notification and improve treatment outcome in Southern Ethiopia. PLoS One. 2013;8(5):e63174.
13. Tulloch O, Theobald S, Morishita F, Datiko DG, Asnake G, Tesema T, et al. Patient and community experiences of tuberculosis diagnosis and care within a community-based intervention in Ethiopia: a qualitative study. BMC Public Health. 2015;15(1):187.
14. Datiko DG, Lindtjørn B. Health extension workers improve tuberculosis case detection and treatment success in southern Ethiopia: a community randomized trial. PLoS One. 2009;4(5):e5443.
15. Degner NR, Joshua A, Padilla R, Vo HH, Vilke GM. Comparison of Digital Chest Radiography to Purified Protein Derivative for Screening of Tuberculosis in Newly Admitted Inmates. J Correct Health Care. 2016;22(4):322-30.
16. Delva GJ, Francois I, Claassen CW, Dorestan D, Bastien B, Medina-Moreno S, et al. Active Tuberculosis Case Finding in Port-au-Prince, Haiti: Experiences, Results, and Implications for Tuberculosis Control Programs. Tuberc Res Treat. 2016;2016:8020745.
17. de Vries G, van Hest RA, Richardus JH. Impact of mobile radiographic screening on tuberculosis among drug users and homeless persons. Am J Respir Crit Care Med. 2007;176(2):201-7.
18. van Hest R, de Vries G. Active tuberculosis case-finding among drug users and homeless persons: after the outbreak. Eur Respir J. 2016;48(1):269-71.
19. Fatima R, Qadeer E, Enarson DA, Creswell J, Stevens R, Hinderaker SG, et al. Success of active tuberculosis case detection among high-risk groups in urban slums in Pakistan. International Journal of Tuberculosis and Lung Disease. 2014;18(9):1099-104.
20. Fatima R, Qadeer E, Yaqoob A, Haq MU, Majumdar SS, Shewade HD, et al. Extending 'Contact Tracing' into the Community within a 50-Metre Radius of an Index Tuberculosis Patient Using Xpert MTB/RIF in Urban, Pakistan: Did It Increase Case Detection? PLoS One. 2016;11(11):e0165813.

21. Ford D, Datta B, Prakash AK, Tripathy JP, Goyal P, Singh S, et al. Fifth year of a public-private partnership to improve the case detection of tuberculosis in India: A role model for future action? Indian J Tuberc. 2019;66(4):480-6.
22. John S, Gidado M, Dahiru T, Fanning A, Codlin AJ, Creswell J. Tuberculosis among nomads in Adamawa, Nigeria: outcomes from two years of active case finding. Int J Tuberc Lung Dis. 2015;19(4):463-8.
23. Kan XH, Zhang LX, Yang JA, Zhang J, Chiang CY. Mobilising elementary and secondary school students for tuberculosis case finding in Anhui, China. Public Health Action. 2012;2(4):152-6.
24. Karamagi E, Sensalire S, Muhire M, Kisamba H, Byabagambi J, Rahimzai M, et al. Improving TB case notification in northern Uganda: evidence of a quality improvement-guided active case finding intervention. BMC Health Serv Res. 2018;18(1):954.
25. Kolappan C, Subramani R, Radhakrishna S, Santha T, Wares F, Baskaran D, et al. Trends in the prevalence of pulmonary tuberculosis over a period of seven and half years in a rural community in south India with DOTS. Indian J Tuberc. 2013;60(3):168-76.
26. Liu K, Peng Y, Zhou Q, Cheng J, Yu H, Tang L, et al. Assessment of active tuberculosis findings in the eastern area of China: A 3-year sequential screening study. Int J Infect Dis. 2019;88:34-40.
27. Lorent N, Choun K, Thai S, Kim T, Huy S, Pe R, et al. Community-based active tuberculosis case finding in poor urban settlements of Phnom Penh, Cambodia: a feasible and effective strategy. PLoS One. 2014;9(3):e92754.
28. Lorent N, Choun K, Malhotra S, Koeut P, Thai S, Khun KE, et al. Challenges from Tuberculosis Diagnosis to Care in Community-Based Active Case Finding among the Urban Poor in Cambodia: A Mixed-Methods Study. PLOS ONE. 2015;10(7):e0130179.
29. Maggard KR, Hatwiinda S, Harris JB, Phiri W, Kruuner A, Kaunda K, et al. Screening for tuberculosis and testing for human immunodeficiency virus in Zambian prisons. Bull World Health Organ. 2015;93(2):93-101.
30. Mallick G, Shewade HD, Agrawal TK, Kumar AMV, Chadha SS. Enhanced tuberculosis case finding through advocacy and sensitisation meetings in prisons of Central India. Public health action. 2017;7(1):67-70.
31. Thapa B, Chadha SS, Das A, Mohanty S, Tonsing J. High and equitable tuberculosis awareness coverage in the community-driven Axshya TB control project in India. Public health action. 2015;5(1):70-3.
32. Thapa B, Prasad BM, Chadha SS, Tonsing J. Serial survey shows community intervention may contribute to increase in knowledge of Tuberculosis in 30 districts of India. BMC public health. 2016;16(1):1155-.
33. Marks GB, Nguyen NV, Nguyen PTB, Nguyen TA, Nguyen HB, Tran KH, et al. Community-wide Screening for Tuberculosis in a High-Prevalence Setting. N Engl J Med. 2019;381(14):1347-57.
34. Miller AC, Golub JE, Cavalcante SC, Durovni B, Moulton LH, Fonseca Z, et al. Controlled trial of active tuberculosis case finding in a Brazilian favela. The international journal of tuberculosis and lung disease : the official journal of the International Union against Tuberculosis and Lung Disease. 2010;14(6):720-6.
35. Parija D, Patra TK, Kumar AMV, Swain BK, Satyanarayana S, Sreenivas A, et al. Impact of awareness drives and community-based active tuberculosis case finding in Odisha, India. International Journal of Tuberculosis and Lung Disease. 2014;18(9):1105-7.
36. Rao VG, Bhat J, Yadav R, Sharma RK, Muniyandi M. Declining tuberculosis prevalence in Saharia, a particularly vulnerable tribal community in Central India: evidences for action. BMC Infect Dis. 2019;19(1):180.

37. Reddy KK, Ananthakrishnan R, Jacob AG, Das M, Isaakidis P, Kumar AM. Intensified tuberculosis case finding amongst vulnerable communities in southern India. Public Health Action. 2015;5(4):246-8.
38. Rendleman NJ. Mandated tuberculosis screening in a community of homeless people. Am J Prev Med. 1999;17(2):108-13.
39. Sanaie A, Mergenthaler C, Nasrat A, Seddiq MK, Mahmoodi SD, Stevens RH, et al. An Evaluation of Passive and Active Approaches to Improve Tuberculosis Notifications in Afghanistan. PLoS One. 2016;11(10):e0163813.
40. Sanchez A, Massari V, Gerhardt G, Espinola AB, Siriwardana M, Camacho LA, et al. X ray screening at entry and systematic screening for the control of tuberculosis in a highly endemic prison. BMC Public Health. 2013;13:983.
41. Shargie EB, Mørkve O, Lindtjørn B. Tuberculosis case-finding through a village outreach programme in a rural setting in southern Ethiopia: community randomized trial. Bull World Health Organ. 2006;84(2):112-9.
42. Shewade HD, Gupta V, Ghule VH, Nayak S, Satyanarayana S, Dayal R, et al. Impact of Advocacy, Communication, Social Mobilization and Active Case Finding on TB Notification in Jharkhand, India. J Epidemiol Glob Health. 2019;9(4):233-42.
43. Tsegaye Sahle E, Blumenthal J, Jain S, Sun S, Young J, Manyazewal T, et al. Bacteriologically-confirmed pulmonary tuberculosis in an Ethiopian prison: Prevalence from screening of entrant and resident prisoners. PLoS One. 2019;14(12):e0226160.
44. Vyas A, Creswell J, Codlin AJ, Stevens R, Rao VG, Kumar B, et al. Community-based active case-finding to reach the most vulnerable: tuberculosis in tribal areas of India. Int J Tuberc Lung Dis. 2019;23(6):750-5.
