# Supplementary material for: Do community-based active case-finding interventions have indirect impacts on wider TB case detection and determinants of subsequent TB testing behaviour? A systematic review
Source: PLOS Glob Public Health. 2021 Dec 8;1(12):e0000088. doi: 10.1371/journal.pgph.0000088 (PMC10021508; doi:10.1371/journal.pgph.0000088)
Supplement: S3 Table — (PDF) [file pgph.0000088.s006.pdf]

Appendix 5 Data extracted from and characteristics of included studies with routine case-notification outcomes

Study characteristics						Population size		Pre period					After (intervention) period				
Study	Country	Population	Distance to healthcare	Cost of healthcare	Access to healthcare	ACF population	Control population	Months	ACF pop cases	ACF pop BC cases	Control pop cases	Control pop BC cases	Months	ACF pop routine cases	ACF pop routine BC cases	Control pop cases	Control pop BC cases
**Cluster-randomised trial**																	
Miller 2010	Brazil	Urban slums	Ready access to local health services. Mean distance bus line to clinic 180 - 250m	TB diganosis and treatment provided free of charge	Standard	24,177	34,410	-	-	-	-	-	9.6	81	81	101	101
**Controlled before-after studies**																	
Aye 2018	Myanmar	Urban slums (& "neighbourhood contacts")	Not stated. Township health centres	Public-private mix. Treatment provided free of charge	Standard	1,696,972	1,700,000	36	7,229	-	12,189	-	36	6,443	-	9,962	-
Cegielski 2013	USA	General population - urban	Not stated. Urban USA so likely not far	Not stated. Most healthcare private in USA	Restricted	3153	155,000	120	15	-	113	-	120	0	-	75	-
Datiko 2017 / Yassin 2013	Ethiopia	Remote rural	Transport facilities limited and relatively expensive, making travel to health facilities challenging	Is of benefit if diagnosis and treatment at low cost to patient'	Restricted	3500000	1,200,000	12	3,968	2,534	2,497	949	54	15,058	5,765	5,483	2,551
Kan 2012	China	General population - rural	Not stated. Township health centres and village doctors	Free services at county dispensary for TB	Restricted	15,443,456	29,256,544	15	-	1,966	-	4,565	15	-	5,014	-	14,353
Parija 2014	India	General population - rural	Not stated. Each village has a CHW	Free TB treatment and diganosis	Restricted	6,090,000	6,060,000	3	-	967	-	364	3	-	831	-	367
Vyas 2019	India	Rural: Indigenous groups	Long distances and no public transport	Not stated. Healthcare often private in India	Restricted	1,000,000	1,000,000	12	1,440	907	1,524	839	12	1,694	711	1,787	793
**Before-after studies**																	
Corbett 2010	Zimbabwe	General population - urban	Lived within 2km of primary care clinic	Not stated. Basic healthcare usually free in Zimbabwe	Standard	110,432	-	6	-	154	-	-	35	-	670	-	-
Fatima 2016	Pakistan	Urban slums "neighbourhood contacts"	Not stated. Urban so unlikely far to BMU	Not stated. Screening and treatment free through NTP in Pakistan	Standard	18,000,000	-	24	100,384	28,159	-	-	24	104,785	26,978	-	-
Fatima 2014	Pakistan	Urban slums perceived high risk or hard to reach	Access to primary health clincs poor although many private clinics'	Screening and treatment free of charge through NTP, but this intervention using private GPs who charge	Hard to reac	6,045,105	-	18	10,374	8,933	-	-	18	11,023	8,275	-	-
Ford 2019	India	Remote rural	Limited access to CXR facilities (part of diagnostic algorthim)	Not stated. Intervention uses public-private mix and healthcare often private in India	Restricted	100,000	-	12	6,599	3,111	-	-	12	6,715	-	-	-
Lorent 2014	Cambodia	Urban slums - perceived high risk or hard to reach	High prevalence and/or restricted access to TB services. Treatment delay due to travel distance and inconveient opening times	Treatment delay due to perceived cost of treatment.	Hard to reac	1,156,466	-	15	4,073	1,610	-	-	15	3,778	1,338	-	-
