# Supplementary material for: Do community-based active case-finding interventions have indirect impacts on wider TB case detection and determinants of subsequent TB testing behaviour? A systematic review
Source: PLOS Glob Public Health. 2021 Dec 8;1(12):e0000088. doi: 10.1371/journal.pgph.0000088 (PMC10021508; doi:10.1371/journal.pgph.0000088)
Supplement: Attachment — Submitted filename: 2021-10_PLoSGH_response-to-reviewers.pdf [file pgph.0000088.s007.pdf]

LONDON SCHOOL of HYGIENE & TROPICAL MEDICINE

Helena Feasey
Research Fellow

London School of Hygiene & Tropical Medicine
Keppel Street, London WC1E 7HT
And Malawi Liverpool Wellcome Research Programme
PO Box 30096 Chichiri, Blantyre 3, Malawi

PLoS Global Health

4th October 2021

Dear Dr Stefan Kohler

Re: PGPH-D-21-00221. Do community-based active case-finding interventions affect subsequent tuberculosis testing and healthcare-seeking behaviour? A systematic review

I write on behalf of my co-authors to thank the reviewers for their careful review, and appreciate the opportunity to respond with a revised submission. Please see a point-by-point response below.

We look forward to your response.

Yours sincerely,

Helena R A Feasey MA MSc (On behalf of the authors)

Editor Comments:

Complementary to the reviewers' comments, I would be interested to better understand how the submitted review and its search strategy relate the following recently published review, which seems to use the same search strategy: https://doi.org/10.1016/s2468-2667(21)00033-5. If the same or a similar search strategy was used, why could the same/a similar search strategy be used to systematically review literature on different research questions? What might be strengths and limitations of the chosen approach?

Thank you for highlighting the other recent systematic review by this group. We did use the same search strategy as the Burke et al review as the starting point for this second review. Both were presented at the WHO meeting as separate PICO questions. Our reason for not including it in Burke Lancet PH were:-

- The PICO questions were different
- The way that TB case notification data were analysed is different (see below)
- In this review we include additional publications identified by systematic search for any qualitative publications from authors included in the Burke review (thus retaining the focus on data originating from studies that have both a case-finding (ACF) intervention and data with a comparison arm).

The reviews were performed in tandem with the same personnel involved in both reviews in order to inform the WHO Technical Working Group's discussions on updating the WHO guidelines on case-finding for TB.

The Burke analysis looked for a direct population-level epidemiological impact, using data on publications that described interventions plus had a comparison group using a. undiagnosed TB (prevalence), b. annual risk of TB infection and c. TB case-notifications (CNRs). The analysis of TB-CNRs did not distinguish mode of detection for the CNRs – just comparison of ACF period against the respective control group.

The focus of the current review was whether or not there was any evidence for an indirect and potentially behavioural effects, using both quantitative and qualitative review. So the questions and analysis are distinct and could not all be reasonably fitted into a single paper.

We consider a unified initial search strategy to be justifiable for the following reasons: the key requirements for a large enough ACF intervention plus a comparison group allowing us to comment on epidemiological effect is a common need for both reviews from the perspective of the analysis of CNRs. The same exclusion and inclusion criteria were therefore employed to identify studies with a suitable design that reported any one of the four types of outcomes (36 studies identified). The studies included in this review are then those that reported on the relevant outcomes (routine case notifications and proxy behavioural outcomes).

We appreciate that we may have missed some relevant qualitative studies that do not fit into our quantitative remit or were published before 2010, but this limitation is now clearly described.

Reviewers' comments:

Reviewer #1: Thank you for having given me the opportunity to review this manuscript. I enjoyed the read!

My main comments concern the search strategy and the conceptual framework that have been used:

- The main search strategy has been copied and updated from Kranzer and colleague's review from 2013. However, the Kranzer review aimed at different research questions (related to number of cases detected, early detection, treatment outcomes, incidence and prevalence), while this review focuses on testing and healthcare seeking. I wonder if the title and research questions of this review may have to be revised to motivate the use of the given search strategy. Moreover, the research questions should be clearly stated.

- The authors mention the COM-B framework and include it in Fig 1. Yet, the motivation for using this framework remains unclear, as well as how exactly it has been applied. I suggest the authors include more information about this in the text and revise Fig 1 for clarity, e.g., clearly highlighting the original elements of the COM-B framework.

We thank the reviewer for their insightful comments, which are extremely helpful, and have helped strengthen the manuscript.

As noted, the Kranzer review focused on cases detected and other TB outcomes. As such it provides an excellent foundation for this accessory review, with the search strategy providing the exact quantitative data needed for our review of both “direct” and “indirect” impacts on TB case notifications. This review was initially conducted as part of a group of reviews (including the recent Burke et al publication) to inform the WHO TB Guidelines, which had a unified search strategy and a starting set of over 20,000 abstracts (one of the major barriers to making sense of the TB case-finding literature).

The research questions in the Kranzer review did not include TB testing or proxy behavioural outcomes for TB and so it is possible that studies previous to 2010 reporting these could have been missed. However, we think this is unlikely since: -

- For our initial search we were looking for only *comparative* quantitative proxy measures of behaviour -- which would then always be a subset of the papers identified in the same search as Burke et al (secondary outcomes for ACF interventions for which primary outcomes are TB case notifications and or prevalence/incidence).
- If a study did not have comparator groups for the primary outcomes it is very unlikely to have had a comparator group from which secondary proxy behavioural outcomes are reported. However, we have added text to emphasise this as a limitation of the study within the discussion section:

“In addition, the Kranzer et al review used for articles published between 1980 to 2010 did not focus on proxy behavioural outcomes so studies reporting on these could have been missed for this period, but as these outcomes are likely to always be secondary to core outcomes of TB notifications and epidemiology (which were included) the likelihood is low.”

We have also updated the title and articulated research questions for this review to reflect the primary focus on indirect impacts of ACF on TB case notifications with the proxy behavioural outcomes as supplementary quantitative outcomes that may give insight into the mechanism leading to changes in the case-notifications. The new title is *“Do community-based active case-finding interventions have indirect impacts on wider TB case detection and determinants of subsequent TB testing behaviour? A systematic review”.*

We have also included additional information about the COM-B model and reasons for applying it, as well as updating Figure 1 to make the COM-B elements and role of case notifications more prominent.

"… ACF can influence subsequent TB testing behaviour through the three elements of the COM-B behavioural theory (capacity, opportunity and motivation) and potentially increase TB case notifications in health facilities through indirect effects (Figure 1). COM-B is a comprehensive model developed from a review of 19 existing behaviourial theories (5) that has been widely applied in assessing and developing public health interventions (5-8) including those for Tuberculosis diagnosis and prevention(9-11)."

Reviewer #2: I think this is an interesting systematic review, and it has been rigorously conducted. However, I had to read it a few times to figure out exactly what was being reviewed in a conceptual sense. I think that the framing of the research question in the introduction and methods needs some revision to make this clear. I also think that some context needs to be provided in multiple places about what the main outcome of routine CNR ratios actually means - that is, what it is potentially telling us, and how a CNR ratio > 1 or <1 should be interpreted.

We thank the reviewer for their helpful comments and the opportunity to improve the paper by addressing them.

In response we have re-framed the research question in the title, introduction and methods to give a clearer conceptual outline. The new title is "Do community-based active case-finding interventions have indirect impacts on wider TB case detection and determinants of subsequent TB testing behaviour? A systematic review" – this places the primary focus on the indirect impacts on case detection, with determinants of behaviour also included as a possible explanation for these indirect effects.

The main aim of our study was to investigate the impact of ACF beyond directly-diagnosed patients. The last sentence of the introduction now reads "*We… aimed to systematically review the evidence of indirect effects of ACF on routine facility-based TB case-notifications and quantitative proxy behavioural outcomes, such as KAP, that could inform the mechanisms underlying any effect on subsequent TB testing behaviour*."

We have also included explanations about the meaning of the routine CNR ratio outcome throughout the manuscript. The following has been added to the introduction: *"Routine CNR ratios with a comparison group (excluding those directly identified through the ACF) >1 would be an indication of indirect impact."*

And in the discussion:

"In this review a routine CNR ratio >1 gives an indication of an indirect effect of ACF on routine case notifications. This was seen in the Miller 2010 RCT (1.14, CI:0.94-1.40) and four of the other studies for all form TB notifications: Aye 2018 (1.09, CI:1.02-1.16), Fatima 2016 (1.04, CI:1.03-1.05), Fatima 2014 (1.06, CI:1.03-1.09), and Ford 2019 (1.02, no CI) but not in any of the bacteriologically-confirmed TB reports. This suggests any indirect impact was unlikely to be due to improved diagnostics implemented through the ACF since this would be expected to be seen primarily in bacteriologically-confirmed rates, but instead may be due to increased TB testing rates and changes in TB testing behaviour."

".... It should be noted that a CNR ratio of ≤1 in this review does not preclude an indirect impact of the ACF on case notifications as this could still occur but be masked by the "substitution" effect, especially when the CNR ratio is 1 or only slightly below (as in Vyas 2019 (1.00, CI:0.95-1.05), Datiko 2017 (0.96, CI:0.88-1.05) and Lorent 2014 (0.93, CI:0.89-0.97) for all forms TB, and Fatima 2016 (0.96, CI:0.94-0.97) and Fatima 2014 (0.93, CI:0.90-0.95) for bacteriologically-confirmed TB). When the CNR ratio is substantially smaller (e.g. Datiko 2017 (0.47, CI:0.41-0.53) and Corbett 2010 (0.75, CI:0.63-0.89) for bacteriologically-confirmed TB) this suggests there is no indirect impact."

Major comments

1) The frame of measuring behavioural outcomes of ACF does not seem to fit the systematic review that was performed. In my opinion, not all of the outcomes measured reflect behaviour, and the search strategy is not comprehensive for studies of behaviour. Regarding the first point, changes in routine case notifications, the primary outcome considered, might not reflect changes in behaviour of the population being screened (see comment 2 for more detail). Moreover, it is hard for me to see additionality and substitution as mainly behavioural outcomes since they have as much to do with the presence or absence of the intervention and how it is implemented as the populations' knowledge, attitudes, or behaviour. Regarding the second point, the inclusion criteria of the initial search automatically narrow the studies to those with study designs for quantitative evaluation. This automatically excludes qualitative research, which is often best suited to understanding behaviour; qualitative research about the impact of an intervention on participants' behaviour would not be done as a pre-post, parallel group comparison, or RCT design. The search for qualitative studies was only done around studies that reported quantitative outcomes, so the search overall is not comprehensive for studies of behaviour. I am not suggesting that the authors re-do their systematic review. Rather, I suggest they re-consider the articulation of their frame in the introduction and methods. To me, the systematic review covers quantitative measures of the indirect impact of ACF that could potentially impact case detection separately from the primary ACF intervention, including (but not limited to) the effects on behaviour. The discussion actually focuses more on the concept of "indirect effects" as opposed to "behavioural outcomes," so the issue is really the framing in the introduction and methods.

Thank you for raising this. We have now re-framed the review to focus on the indirect effects of ACF on TB case detection with changes in rates of TB testing and TB testing behaviour as a possible explanation for this. As now highlighted in the introduction, this review originally came about as we observed a strong indirect effect of an ACF intervention in Blantyre and so aimed to understand if this had happened elsewhere and if so whether there was evidence that this was due to changes in testing rates and TB testing behaviour. We have also added text to outline that the initial review is specifically of quantitative measures with qualitative evidence of behaviour only included within the search of secondary manuscripts. New text added to the introduction and methods to reflect this include:

"ACF interventions are designed to directly identify people living with undiagnosed TB in the community but may also have an indirect impact on wider TB case detection as was seen in a 2011-14 ACF intervention in Blantyre, Malawi where routine case-notifications increased substantially over the intervention period (5). Routine CNR ratios with a comparison group (excluding those directly identified through the ACF) >1 would be an indication of indirect impact. This indirect impact could be due to enhanced diagnostics introduced through the intervention or an impact on subsequent community TB testing rates and behaviour. Enhanced diagnostics could increase routine case notifications through improved test sensitivity, although this is likely to be limited to bacteriologically-confirmed TB and there may be a concurrent drop in clinically-diagnosed TB, but potentially health workers could also offer more TB tests if aware of the enhanced diagnostic capacity, leading to higher testing rates. Higher TB testing rates could be due to changes in health worker or community behaviour."

2) I think that the distinction between overall case notifications (direct impact of ACF) and impact on routine case notifications (indirect impact) needs to be made clearer either in the introduction or earlier in the methods, and the rationale should be given for considering changes in routine case notifications to be a proxy for behavioural outcomes. It is not clear why routine case notifications excluding the direct yield of the ACF intervention are a proxy behavioural outcome. Unlike KAP instruments, which are meant to measure behaviour, changes in routine case notifications may not in fact reflect changes in behaviour in the population being screened for TB. For example, a positive impact could reflect health system strengthening as a result of an ACF intervention. For example, an ACF intervention may rely on Xpert testing provided in the existing laboratory infrastructure, and to accommodate the increase testing burden, the procurement system for cartridges is strengthened, and

procedures for sputum storage and processing are improved. This improvement in the Xpert infrastructure could conceivably end up increasing routine case notifications if it leads to an increase in Xpert testing (or a shift from smear microscopy to Xpert), even if it is not actually changing the behaviour of people who are seeking care for TB symptoms. Alternatively, if the framing is revised to shift away from a focus on behavioural outcomes, the introduction/methods still needs some rationale for why changes in routine case notifications excluding direct ACF diagnoses are an outcome of interest. The bulk of the results focus on these CNRs, so it is very important for the reader to understand why they are important.

Thank you. As suggested, we have re-framed the introduction to focus on changes in routine case-notification rates as a potential indirect effect of an ACF intervention. Changes in TB testing behaviour are a possible explanation for changes in routine CNRs and we therefore also aimed to identify and review quantitative proxy behavioural outcomes that could be directly attributed to ACF through the use of a comparison group. We also specifically address the potential impact of health system strengthening and recorded whether this was part of the included studies. This is reflected throughout the manuscript and changes include:

"Only Datiko 2017 and Lorent 2014 reported improving routinely available TB diagnostics as part of the intervention."

"… In this review a routine CNR ratio >1 gives an indication of an indirect effect of ACF on routine case notifications. This was seen in the Miller 2010 RCT (1.14, CI:0.94-1.40) and four of the other studies for all form TB notifications: Aye 2018 (1.09, CI:1.02-1.16), Fatima 2016 (1.04, CI:1.03-1.05), Fatima 2014 (1.06, CI:1.03-1.09), and Ford 2019 (1.02, no CI) but not in any of the bacteriologically-confirmed TB reports. This suggests the indirect impact was unlikely to be due to improved diagnostics implemented through the ACF since this would be expected to be seen primarily in bacteriologically-confirmed rates, but instead may be due to increased TB testing rates and changes in TB testing behaviour. In addition, an indirect effect was not observed in the only two studies which did report improved diagnostics (Datiko 2017 & Lorent 2014)."

3) Since CNR ratios are the main outcome, the methods should explicitly describe how they were extracted and the assumptions made in the calculations. The current description "To establish routinely diagnosed case notification rates, person-years of follow-up and notified TB cases diagnosed only through routine screening activities were extracted or calculated from available data using simple arithmetic" is vague. What is the denominator for the case notification rate? The target population or the number of people screened? If target population, and if the size of the target population is not reported separately for the pre-intervention and intervention periods, is the assumption made that the size of the underlying population size does not change (so the ratio of CNR is equivalent to the ration of notifications)?

Thank you for highlighting this. We have added more detailed explanations of how the CNR ratios were calculated in the methods section as below:

"Person-years were calculated for the target populations for which case-notifications were reported. For before-after studies if the size of the population was not reported separately for the pre- and post-intervention periods it was assumed the size of the population did not change."

Regarding the numerator for routine CNR, I assume that the authors took the reported total case notifications and subtracted the diagnoses made directly by the ACF intervention. If not, then the calculation needs to be described. If so, then it seems that the assumption is being made that ACF diagnoses are a subset of the total notifications. This assumption is often made in calculation of additionality although it is not completely true, as ACF interventions often diagnose people from outside the target population (e.g. a person visiting from another district may be diagnosed by the ACF intervention but their notification is actually tied to their home district and thus not counted in the notifications being used for the evaluation).

The calculation is as the reviewer has suggested and we have added text to make this explicit in the methods section: *"None of the studies presented case-notification ratios for routine diagnosis; we calculated these through subtracting the available ACF-specific case notifications from the overall notification data."*

This is indeed an assumption, and so we have included this as a limitation in the discussion: *"We also assumed that ACF diagnoses are a subset of the total notifications but an ACF diagnosis could then become a notification in another population for example through population movement, although this is not reported by any of the studies."*

4) In the methods, it seems that access to healthcare is being determined by authors. It is unclear how they make this decision since the definitions are based on features of the health system and population that are not always reported. For instance, the average distance that patients travel to a health facility is not a standard feature reported in many "study setting" descriptions. If there are assumptions being made, these need to be stated.

Thank you. The access to healthcare classifications were made based on what was reported in the relevant studies and to clarify this we have updated this section in the methods to: *"We classified studies according to level of healthcare access within the target population based on distance to and cost of care, as indicated by the reported context or assumed from knowledge of the local health system (Appendix 5)."* The information and any assumptions made have been included in a new appendix which also contains the extracted data for the CNR ratio calculations.

5) Inclusion and inclusion criteria used in various phases of the search are not clear. What were the inclusion criteria for the abstract/title review versus the main text review?

Which step of the PRISMA flow diagram reflects the title/abstract versus the full text review? By convention, I would guess that the first box that simply says "articles not eligible for inclusion" reflects the title/abstract review, but neither the methods nor the flow diagram explain what was being selected for at this phase.

The same inclusion and exclusion criteria were used for the abstract/title review and the main text review. If studies were not excluded based on the abstract/title review they were part of the main text review. We have updated the methods to reflect this: *"Studies identified through the updated search were title and abstract double screened for eligibility … and then the full text of those not excluded and all studies from the Kranzer and colleagues review were reviewed by two of HRAF, RMB and MN."*

The PRISMA flow diagram has also been updated to show that the first box on exclusion refers the title/abstract review.

Similarly, the inclusion criteria for the second phase of the search are not clear. Line 224-225 "qualitative or quantitative data on the impact of ACF on TB health seeking behaviour" is vague. Since two papers were excluded for not reporting KAP results in a specific way, the authors presumably had specific criteria for what they included here.

We have also added additional text to the methods to give further explanation on the inclusion criteria for the second phase of the search. This now reads: *"To be included, the study had to be part of the ACF intervention study identified through the main literature search and include qualitative or quantitative data on the impact of the ACF itself on community TB health seeking behaviour (KAP, TB testing behaviours, pathways to care, TB stigma or social norms). Studies not specifically demonstrating the impact of the ACF on these factors in the ACF target population were excluded, e.g. if the KAP measures were for a different population."*

6) The narration of the included studies/articles appears inconsistent between the figure and different parts of the results.

- The PRISMA diagram shows 13 articles reporting routine TB case notification rate analyses and 1 article reporting behavioural outcomes.

- Table 1 shows 12 articles reporting TB case notification rates and 1 article reporting KAP outcomes.

- The first paragraph of the results say 12 articles reported case notification data, 1 from the initial search reported behavioural outcomes, and then from the second phase of the search, "three studies were eligible for inclusion as KAP/qualitative manuscripts."

- The results section "KAP and qualitative studies" starting at line 379 says "Of the four publications identified, two were excluded from further analysis."

Please make all of the reporting consistent. There seems to be a discrepancy about whether there were 12 or 13 articles identified in the first phase of the search and 2, 3, or 4 identified during the second phase of the search.

Thank you. We have ensured that any discrepancies have been corrected. There were 12 studies identified reporting routine TB case-notifications but 13 articles as indicated in the PRISMA diagram. Two articles (Yassin 2013 and Datiko 2017) were about different components of the same study. Table 1 has been updated to include the Yassin 2013 study with the Datiko 2017 study to make this clear. The first paragraph of the results states '12 studies' which also reflects this.

The discrepancies on the secondary KAP/qualitative manuscripts have been removed to ensure that the final number included is always 2. This reflects that 2 of the 4 manuscripts initially included reported a KAP study nominally linked to a multistate intervention in India (Project Axshya – see Burke Lancet PH), which were subsequently removed as timing and catchment populations of the KAP studies did not relate clearly to the ACF interventions. This is touched on in the results section (P21 – under subheading "Linked KAP and qualitative studies" *"the KAP surveys were not aligned to the populations or timing of the ACF interventions."*

Moreover, since one expects the first paragraph of the results to summarize the identification of studies, it would make sense to report the total number of included studies identified by the second search in this paragraph rather than only the number "eligible" (presumably this means after the abstract review?) and then wait until much later in the results to report the number actually included.

Th first results paragraph has been updated to include all of this information at the start of the results section: *"Only one out of the 36 manuscripts reported any proxy behavioural outcomes (25), but the additional search identified 1121 manuscripts, of which four articles were eligible for inclusion as KAP/qualitative manuscripts after full text review, but two of these were excluded from full analysis following identification of additional documentation (Appendix 4)."* In addition, Appendix 4 lists all the studies included in the secondary full text review and where excluded the reasons for doing so.

7) Since the two searches are part of a single systematic review, then I believe that the additional qualitative/behavioural identified through the second phase of the search should included in Table 1.

We have added the two additional qualitative/KAP studies identified through the second phase of the search to Table 1 as an additional column indicating which of the other outcome studies they relate to.

Ideally the second phase of the search and what it yielded would also be part of the flow diagram, although I realize this would make the diagram complex (although there is a lot of blank space right now, so if the boxes were more efficiently positioned, the additional search process might fit).

We have also updated the PRISMA diagram to include the second phase of the search.

8) In Figure 3, the horizontal axis has to be labeled. It is not clear what periods reflect pre-implementation, implementation, and post-implementation phases.

We have added labels to the horizontal axis on Figure 3 to indicate the pre-intervention, intervention, 60 days post intervention and more than 60 days post intervention periods.

9) There needs to be some consistency in how different periods are referred to. Pre-post studies typically include a period prior to implementation of an ACF intervention, and a period during which an ACF intervention period is being implemented; evaluating a period after the intervention has stopped being implemented is also possible, but not as common. The paper uses various terms to refer to different periods, and I am not always sure what is being referred to. In line 333, does "post-intervention" refer to the period during the intervention or after the intervention has been completed? Similarly, in lines 432 and 438, does "following community ACF" (lines 432, 438) mean during the implementation of a community ACF program, or after the program has ended? Figure 4 uses the terms "baseline" and "endline" while figure 5 uses "before" and "After."

Thank you for highlighting this. As mentioned for all of the studies the 'post' period for all the studies (except Miller et al) is limited to the time during which the ACF intervention is being implemented. We have added additional text to make this clear: " For all studies (except Miller 2010) the "after" or outcome notifications period was the period during the intervention and did not extend beyond."

In addition, we have updated line 333 (now line 384), line 432 (now line 494) and line 438 (now line 501) to refer to "during" instead of "post" intervention rates.

The labels on Figure 4 have also been updated to "before" and "after" to match figure 5 as well as reflect description of these as 'before-after' studies.

10) Based on the data availability statement, all data are available in the manuscript and appendices. However, the quantitative data extracted from the individual articles does not appear anywhere in the manuscript. There is no way to tell the sizes of these studies or the numbers of cases detected. There is no way to "back into" these numbers from the CNR ratios reported.

Apologies for this oversight. We have added a new appendix – Appendix 5 which includes the extracted data from which the CNR ratios were calculated.

11) I think that paragraphs 2 and 3 of the discussion are critical because they help the reader to interpret what the CNRs reported in the results mean. However, I find these paragraphs difficult to understand. What is the impact that one expects to see if ACF has a positive impact on health-seeking behaviour? What impact might strengthening of diagnostic services have? There are a lot of references back to specific tables and figures, which is awkward in the discussion. Rather than referring back to specific tables and figure in the results, I think that these paragraphs should try to articulate the big picture of what one expects to see and then offer an interpretation of what was observed.

Thank you. We have added to and updated the discussion to articulate the impact expected if ACF has a positive indirect impact on case notifications and what impact strengthening diagnostic services may have. We have also included specific results within the text to reduce the need to refer back to the tables and figures. Some of the updated discussion now reads as:

"In this review a routine CNR ratio >1 gives an indication of an indirect effect of ACF on routine case notifications. This was seen in the Miller 2010 RCT (1.14, CI:0.94-1.40) and four of the other studies for all form TB notifications: Aye 2018 (1.09, CI:1.02-1.16), Fatima 2016 (1.04, CI:1.03-1.05), Fatima 2014 (1.06, CI:1.03-1.09), and Ford 2019 (1.02, no CI) but not in any of the bacteriologically-confirmed TB reports. This suggests the indirect impact was unlikely to be due to improved diagnostics implemented through the ACF since this would be expected to be seen primarily in bacteriologically-confirmed rates, but instead may be due to increased TB testing rates and changes in TB testing behaviour. The limited evidence available suggests that there may be a difference in impact between the two forms of TB."

"… It should be noted that a CNR ratio of ≤1 in this review does not preclude an indirect impact of the ACF on case notifications as this could still occur but be masked by the "substitution" effect, especially when the CNR ratio is 1 or only slightly below (as in Vyas 2019 (1.00, CI:0.95-1.05), Datiko 2017 (0.96, CI:0.88-1.05) and Lorent 2014 (0.93, CI:0.89-0.97) for all forms TB, and Fatima 2016 (0.96, CI:0.94-0.97) and Fatima 2014 (0.93, CI:0.90-0.95) for bacteriologically-confirmed TB). When the CNR ratio is substantially smaller (e.g. Datiko 2017 (0.47, CI:0.41-0.53) and Corbett 2010 (0.75, CI:0.63-0.89) for bacteriologically-confirmed TB) this suggests there is no indirect impact."

12) The discussion would benefit from a discussion of how the authors believe that studies should evaluate indirect impacts on behaviour. I am not convinced that routine CNR ratios are the most meaningful measure of this, but I would be open to an argument to convince me. How would one ideally measure impact of an ACF program on care-seeking behaviour, stigma, or norms? Pre-post testing? Longitudinal testing on the same individuals (i.e. the same individuals are evaluated before and after the program is implemented in their community) or cross-sectional testing in on population samples before and after implementation? What kinds of instruments should be used? What is the role of qualitative or mixed-methods research?

We have expanded the discussion of how best to measure the indirect impacts of an ACF intervention and which outcome measures would be most appropriate. The additional text includes:

"Disappointingly, we found no studies reporting TB testing rates which would have allowed us to distinguish whether increases in routine TB case notifications were likely due to an increase in testing or enhanced sensitivity of improved diagnostics with a constant testing rate."

"…Our main recommendations are to strengthen the evidence regarding ACF and indirect effects on subsequent TB notifications and testing behaviour. Qualitative and quantitative assessment of the indirect effects of ACF should be conducted prospectively. Testing rates would be a better outcome measure than case notifications to establish indirect impact on TB testing behaviour but these are not routinely collected. Case notifications, and TB testing where available, from both ACF and routine diagnostic services should be reported separately, ideally including pre-ACF, during-ACF and post-ACF periods, evaluated against a comparator population. The inclusion of a comparator is critical, as this is what allows attribution of impact to the ACF intervention itself. To better understand the mechanisms through which ACF potentially impacts TB testing behaviour, relevant outcomes including TB KAP, test initiator (patient or health worker), stigma and norms should be investigated and reported, ideally through repeated cross-sectional sampling before and after implementation. Accompanying qualitative research would provide the rich detail needed to understand how the ACF intervention creates these indirect impacts on subsequent TB case detection."

Minor comment

In reviewing prior research that has established the direct impact of ACF, it is worth mentioning the Cochrane review that encompassed this topic in addition to the more recent review by Burke et al and the older review by Kranzer et al (Mhimbira et al. Interventions to increase tuberculosis case detection at primary healthcare or community-level services, 2017).

Thank you. We have added named references to all these previous reviews on the direct impact of ACF in the introduction*: "Previous systematic reviews by Kranzer et al (2013)(13), Mhimbira et al (2017)(14) and Burke et al (2021) (15) have shown that ACF interventions can initially increase TB case notifications, but not invariably."*
