# Supplementary material for: Do community-based active case-finding interventions have indirect impacts on wider TB case detection and determinants of subsequent TB testing behaviour? A systematic review
Source: PLOS Glob Public Health. 2021 Dec 8;1(12):e0000088. doi: 10.1371/journal.pgph.0000088 (PMC10021508; doi:10.1371/journal.pgph.0000088)
Supplement: Attachment — Submitted filename: 2021-10-27_PLoSGH_response-to-reviewers2.docx [file pgph.0000088.s008.docx]

PLoS Global Health

27^th^ October 2021

Dear Dr Stefan Kohler

Re: PGPH-D-21-00221R1 Do community-based active case-finding interventions have indirect impacts on wider TB case detection and determinants of subsequent TB testing behaviour? A systematic review

I write on behalf of my co-authors to thank the reviewers for their updated review, and appreciate the opportunity to respond with a revised submission. Please see a point-by-point response below.

We look forward to your response.

Yours sincerely,

Helena R A Feasey MA MSc (On behalf of the authors)

Reviewers' comments:

Thank you to the readers for their work on this revision. I think that the new frame of focusing on indirect effects of active case-finding works well. The discussion is very good and helps the reader to understand the interpretation of the CNR ratios. The inclusion of Appendix 5 makes the data and results transparent. However, there are still a few points that I find confusing.

1) The abstract is a little confusing, particularly with the new frame of the paper. The methods section of the abstract mostly focuses on the behavioral outcomes but the results are all about the CNR ratios. Because the abstract does not really make clear what the indirect effects of interest are, it is a little confusing to follow. Perhaps the authors could make it clearer by spending more of the word count explaining how routine CNR ratios are a routine measure of indirect effects and less on the behavioral outcomes for which no results were actually found.

Thank you for highlighting this. We have updated the abstract to ensure the measurement of indirect effects are given higher priority than behavioural outcomes throughout the abstract sections. The individual behaviourial outcomes have been removed from the methods and wording has been re-ordered to ensure indirect effects are the focus, with behaviour as one possible contributor. Updated text includes:

“We investigated the impact of ACF beyond directly diagnosed patients for TB, using routine case-notification rate (CNR) ratios as a measure of indirect effect….

… ACF interventions with routine CNR ratios >1 suggest an indirect effect on wider TB case-detection, potentially due to impact on subsequent TB testing behaviour through follow-up after a negative ACF test or increased TB knowledge.”

2) Based on the flow diagram, I still do not think the inclusion/exclusion criteria are sufficiently explained in the methods. One has to piece together what the authors were looking for by looking at the flow diagram and the results, which should not be the case. The inclusion criteria section of the methods say that studies had to evaluate an ACF intervention via a comparison between exposed/unexposed populations, report either routinely diagnosed case notifications, have adults, and screen at least 1000 people. A few points about this are confusing to me.
   a. The authors actually calculated the “routine case notification rates” (which they define as excluding the ACF cases) by subtracting the direct yield of the ACF intervention from total case notifications, so the papers were not actually reporting what the authors define as routine case notification rates. (This calculation, as I mentioned in my previous review, makes certain assumptions about underlying populations, so reporting ACF direct yield and overall case notifications is not the same as reporting case notifications that are not attributable to ACF). I assume that the actual inclusion criterion was having specific types of information that would allow the calculation of CNR (e.g. direct yield and overall case notifications reported for a matching time period, perhaps with some sort of assurance that they reflect the same underlying population); it would be good for the authors to describe these.

Thank you. We have now outlined that (as you suggest) studies were included that either reported routine case notification rates or both overall case notifications and direct yield from ACF enabling us to calculate presumed routine case notification rates (lines 219-221).

“Routinely-diagnosed TB notification outcomes could either be directly reported or calculated if both direct ACF yield and overall case notifications were reported for the same period and relevant population.”

This assumption is included in the limitations of the study

“We also assumed that ACF diagnoses are a subset of the total notifications but an ACF diagnosis could then become a notification in another population for example through population movement, although this is not reported by any of the studies.”

b. Contact investigations were excluded, but this information is only given in a previous section about the definition of ACF (section starting at line 165) as opposed to in the inclusion/exclusion criteria (section starting at line 213). I believe that sending health care workers into the community to actively screen contacts is generally included under the ACF umbrella. If the authors believe that community-based contact screening is not ACF, then the current statement in the ACF definition should be revised to justify this. If the authors believe that community-based contact screening is ACF, but just not a type relevant to this review, then this explanation and justification should be moved to the exclusion criteria section, not the definition of ACF.

Thank you for the opportunity to clarify this. We certainly agree that contact screening is a form of ACF and is referred to as such by the WHO, but it was not included within this review which focused on ACF for broader community populations. To reflect this, as suggested, we have moved the explanation and justification to the exclusion criteria section, instead of the definition of ACF.

c. I am still not clear about how the authors applied the same inclusion/exclusion criteria at both the title/abstract and full text review stage. In my experience conducting systematic reviews, the inclusion/exclusion criteria at the title/abstract stage are less detailed/stringent because papers often do not report a lot of detail in the abstract. Did the authors exclude any articles that did not explicitly report the items on their inclusion criteria list in the abstract? In particular, it seems unlikely to me that one can accurately assess from an abstract whether a paper will report both ACF direct yield and case notifications in a way that allows proper calculation of CNRs. Since >95% of the articles were excluded at the title/abstract stage and no specific exclusion criteria are shown in the flow diagram, assessing the quality of the review requires understanding what the authors did to exclude these articles.

Thank you. We have updated the search strategy section to fully outline the eligibility criteria used in the abstract and title review:

“Studies identified through the updated search were title and abstract double screened for initial eligibility (original research, where ACF had taken place, written in English, French or Spanish) by FN, AES and LHC.”
